# Economic gains from hypothetical improvements in the psychosocial work environment: A cohort study of 71 207 workers in Denmark

**DOI:** 10.5271/sjweh.4244

**Published:** 2025-11-01

**Authors:** Brian Krogh Graversen, Kristian Schultz Hansen, Reiner Rugulies, Jeppe Karl Sørensen, Ann Dyreborg Larsen

**Affiliations:** 1 National Research Centre for the Working Environment, Copenhagen, Denmark.; 2 Section of Epidemiology, Department of Public Health, University of Copenhagen, Denmark.

**Keywords:** costs of illness, healthcare use, occupational health, parametric g-formula, sickness absence, simulation study

## Abstract

**Objectives:**

There is increasing interest in the economic effects of improving working conditions, however, evidence is sparse. This study aims to estimate the economic effects of hypothetical improvements in the psychosocial work environment (PSWE) experienced by Danish workers.

**Methods:**

We included 71 207 workers, reporting information on their psychosocial working conditions in the “Work Environment and Health in Denmark” survey and linked these workers to population-based register data. We used the parametric g-formula method to estimate the economic effects of hypothetical improvements of the general PSWE, in terms of costs related to sickness absence and healthcare use. We further examined which PSWE factors contributed most to the economic effects.

**Results:**

A hypothetical improvement of the PSWE – from the least to the most desirable situation – resulted in an annual gain of €1685 [95% confidence interval (CI) €1234–2135] per worker. When analyzing an improvement from the observed to the most desirable situation, the gain became weaker (€305, 95% CI €134–476). Gains were largely driven by reductions in sickness absence and were larger for women than men and for public sector workers than private sector workers. The PSWE factors with the largest contribution were eliminations of threats of violence and improvements in quality of leadership and social support from colleagues (least to most desirable) and improvements in social support from colleagues, influence at work and quality of leadership (observed to most desirable), respectively.

**Conclusions:**

Hypothetical improvements in the PSWE resulted in substantial economic gains, mostly driven by savings related to sickness absence.

Exposure to adverse psychosocial working conditions can be hazardous for workers’ physical and mental health (1, 2). Despite the potential risks, however, many workers face such exposure (3). Encouragingly, a recent overview review suggests that organizational-level interventions aimed at improving psychosocial working conditions can be effective for improving workers’ health and well-being (4).

In occupational and public health research, there is increasing interest not only in whether psychosocial workplace interventions protect and improve workers’ health but also whether such interventions generate economic gains, both at the company level (eg, by reducing absenteeism and increasing productivity) and the societal level (eg, by reducing public healthcare use and disability pensioning) (5–9). However, as pointed out in recent editorials, research on the economic effects of workplace interventions is still in its infancy, with few studies of acceptable quality (5, 6).

In the present article, we first estimate how the societal costs of sickness absence (SA) and healthcare use of workers are associated with exposure to various psychosocial working conditions. Subsequently, we estimate the reduction in costs of SA and healthcare use from simulated hypothetical improvements of these working conditions.

The estimation of the effects of hypothetical work environment improvements on specific outcomes is a relatively new approach in occupational and public health research. The approach originates from causal inference debates and the use of the parametric g-formula that allows simulating hypothetical interventions using observational data (10, 11). Our article is particularly inspired by two recent studies of Mathisen et al who used the parametric g-formula to estimate the effects of hypothetical improvements in the psychosocial work environment (PSWE) on job turnover (12) and SA rates (13) among Danish hospital workers. Our study differs, though, from Mathisen et al's studies in that it estimated the economic effects of the hypothetical interventions and was not restricted to hospital workers but rather used data from a nationally representative survey of Danish workers.

More specifically, the present study aimed to estimate: (i) the economic effects of hypothetical improvements of the general PSWE experienced by Danish workers, in terms of changes in costs of SA and healthcare use, and (ii) the economic effects of hypothetical improvements of specific PSWE factors, ie, to identify those PSWE factors whose improvement offers the greatest potential economic gains.

## Methods

### Study population

The study population consisted of 18–64-year-old workers invited to participate in one or more of the four waves of the national survey “Work Environment and Health in Denmark” (WEHD) conducted in 2012, 2014, 2016 and 2018 (14–16). The workers were selected by a mix of random sampling from the general population of workers and stratified sampling from selected workplaces. Respondents from the general worker population in the 2012 and 2016 waves were invited to participate in subsequent waves (ie, in 2014, 2016 and 2018 for respondents from 2012 and in 2018 for respondents from 2016). By design, workers can be respondents in one to four waves.

**Figure 1:**
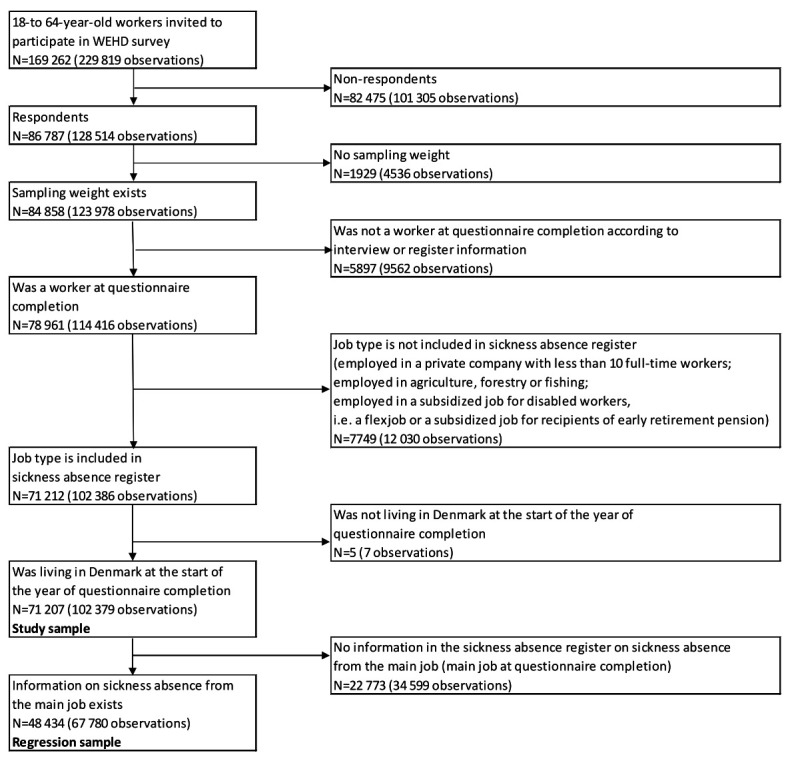
Flow-chart.

The WEHD data provides information about respondents’ work (including work environment characteristics), health and health behaviors. We linked this information with detailed register information on employment periods, wage payments, job characteristics, workplace characteristics, sociodemographic characteristics, SA and healthcare use. There were 86 787 workers responding in one or more survey waves (figure 1). On average, each of these workers responded in 1.5 waves, yielding 128 514 observations. Across the four waves, the response rate was 55.9%.

For each survey wave, Statistics Denmark have calculated sample weights for the respondents to make the sample of respondents representative of all workers in Denmark meeting the standard requirements to be included in the WEHD survey: (i) liable to pay taxes in Denmark, (ii) registered with an address in Denmark, (iii) ≥35 monthly working hours and (iv) earning ≥€402 (3000 Danish kroner) per month. In some cases, respondents from 2012 and 2016, who were invited in subsequent waves, did not meet these requirements. Statistics Denmark disregarded observations not meeting the requirements when calculating the sample weights and thus did not calculate sample weights for such observations.

We excluded responses with missing sample weights and responses from individuals who were not wage earners at questionnaire completion. Furthermore, we excluded responses from workers employed in private companies with <10 full-time workers, workers employed in agriculture, forestry or fishing, and workers employed in a subsidized job for disabled workers. Such workers are not included in the SA register and are therefore uninformative about the association between PSWE factors and SA.

Finally, we excluded responses from individuals not living in Denmark at the start of the year of questionnaire completion. We ended up with a study sample containing 71 207 workers and 102 379 observations.

For part of our analyses (when estimating regression models), we used a smaller sample excluding responses from workers with missing information on SA from their main job (defined as the job with most working hours at questionnaire completion). This sample, which we will refer to as the “regression sample”, included 48 434 individuals and 67 780 observations. The main reason for missing SA information is that only a selected sample of private companies with 10–249 full-time workers have to report information on their workers’ SA to the SA register. The probability for a company of such size to be included in the sample having to report SA increases with the number of full-time workers in the company. All public workplaces and private companies with ≥250 full-time workers report SA information on their workers.

### Psychosocial work environment factors

We measured 17 PSWE factors, including 7 multiple item scales and 10 single item scales. The 17 PSWE factors cover key concepts from the Copenhagen Psychosocial Questionnaire II (COPSOQ II) (17), and belong to 6 domains: (i) Demands at work (measured by the factors quantitative demands, work pace and emotional demands); (ii) Work organization and job contents (measured by influence at work); (iii) Interpersonal relations and leadership (role clarity, quality of leadership, social support from colleagues, recognition from colleagues); (iv) Work-individual interface (job insecurity); (v) Values at the workplace (justice, social inclusiveness); and (vi) Offensive behaviors (conflicts and quarrels, bullying, witnessing bullying, physical violence, threats of violence, sexual harassment). The six domains, the 17 PSWE factors, and the 35 items measuring these factors are listed in the supplementary material (www.sjweh.fi/article/4244, table S1). In this table, we also list the Cronbach’s alphas for the 7 multiple item scales.

The 35 items were partly derived from the COPSOQ II and partly developed specifically for the WEHD questionnaire, in collaboration with researchers and stakeholders, including employer organizations, labor unions, and the Danish Working Environment Authority.

We collapsed items into scales by converting the individual item 5-point Likert values to scores of 0–100 (ie, 0, 25, 50, 75 or 100), with higher scores indicating a more desirable PSWE, and then averaging the scores of items included in a given scale. If some of the item scores were missing, averaging was made over the non-missing items only, and if more than half of the scores of the included items in a scale were missing, the scale value was set to missing.

For the first 11 scales belonging to domains (i)–(v), we categorized non-missing scale values into three groups by the 25^th^ and 75^th^ sample-weighted percentiles (or as close as possible) in the study sample, indicating “least desirable”, “medium” and “most desirable” working conditions, respectively. We categorized observations with missing scale values into a fourth group named “unknown”. As a special case, for the quality of leadership scale, we split observations with missing scale values into two groups: “no superior” (missing scale value because worker had no superior) and “unknown” (missing scale value because too few questions included in the scale were answered).

For the last 6 scales belonging to domain (vi) and describing various types of offensive behavior, we categorized scale values into three groups: “least desirable” (had experienced the given offensive behavior; scale value 0–75), “most desirable” (had not experienced the given offensive behavior; scale value 100) and “unknown” (missing scale value).

### Outcome variables

When information on SA from a worker’s main job was available in the SA register, we measured the worker’s hours of SA in the main job within a one-year window starting at the date of questionnaire completion. If the job ended (or information on SA in the job was no longer available) before the end of the one-year window, SA was only measured as long as the job (or SA information) lasted. Applying the human capital approach, we calculated the societal costs (ie, the reduction in society’s production value) of a worker’s SA as hours of SA times the worker’s productivity per working hour, measured by the hourly wage rate (18).

Based on information from healthcare registers, we measured the costs of workers’ healthcare use over the same period as we measured hours of SA, ie, a period of up to one year. We computed four healthcare cost measures for each worker: costs of prescription drug use, costs of primary healthcare use (includes, among other things, services delivered by general practitioners and specialist doctors), costs of non-psychiatric hospital treatment and total healthcare costs (defined as the sum of the three preceding healthcare cost measures).

Costs of prescription drug use included costs of prescription drugs collected during the outcome measurement period. Costs of primary healthcare use included costs of primary healthcare services invoiced by healthcare providers during the outcome measurement period (visit dates were not available in the data). Costs of outpatient hospital treatment included costs of outpatient treatment received during the outcome measurement period. Costs of inpatient hospital treatment included costs of inpatient treatment received during the outcome measurement period. If an inpatient hospital treatment episode started before or ended after the outcome measurement period, we included a proportion of the treatment episode costs corresponding to the proportion of the treatment episode that overlapped with the outcome measurement period.

In Denmark, healthcare is predominantly publicly provided and financed and our data included information on publicly financed or subsidized healthcare services only (19, 20). Costs of prescription drug use included public subsidies and individuals’ out-of-pocket co-payments. Costs of primary healthcare use and costs of hospital treatment only included publicly financed costs. All costs are in 2023 euros.

### Covariates

As covariates, we included (i) sociodemographic characteristics (sex, age, education, immigrant status, family type and interaction of age of the youngest child in the family with worker’s sex), (ii) job characteristics (work type, sector, industry, occupation, seniority, actual weekly working hours, time of day working, commuting time, number of full-time workers at the local workplace and number of full-time workers in the overall company), (iii) physical work environment characteristics (exposure to various physical working conditions and physical strenuousness of work), (iv) health status characteristics (body mass index and variables describing current or previous treatment for diseases) and (v) health behaviors (smoking behavior, alcohol consumption and exercise behavior). All these covariates were measured at or before questionnaire completion.

We also included a survey wave indicator and a variable measuring length of the period over which we measured hours of SA and costs of healthcare use (since our outcome measures increased with measurement period length).

Supplementary Appendix 2 provides more details on the covariates.

### Analytical framework

All analyses were performed using sample weights to make the results representative for the source population from which our study sample was drawn (the WEHD source population excluding workers in private companies with <10 full-time workers, workers in agriculture, forestry or fishing, and workers in subsidized jobs for disabled workers).

Like Mathisen et al (12, 13), we applied a simplified version of the parametric g-formula without time-varying variables (21, 22) to estimate changes in costs of SA and healthcare use from hypothetical improvements of the PSWE. The parametric g-formula method uses regression models to estimate potential population-level outcome effects from a hypothetical intervention by comparing simulated outcomes for a given population in two scenarios: without and with the intervention.

We analyzed two specific types of hypothetical interventions. The first intervention type changed the PSWE from a situation where all workers experienced the least desirable PSWE to a situation where all workers experienced the most desirable PSWE (measured by either one specific PSWE factor or all PSWE factors). This intervention resembles a randomized controlled trial comparing an all-exposed group with an all-unexposed group (23).

The second intervention type changed the PSWE from a situation where the workers experienced their observed PSWE to a situation where all workers experienced the most desirable PSWE (again measured by either one or all PSWE factors). This intervention is equivalent to an intervention removing a harmful exposure from a real-world setting (23).

We used hurdle models to generate outcome predictions for the scenarios that we compared. Hurdle models are appropriate because our outcome variables (costs of SA and healthcare use) have many zero observations and a right-skewed distribution for the observations with positive value. Supplementary Appendix 3 provides details on the computational algorithm.

As previous studies have reported gender differences in the association between PSWE factors and SA, all analyses were performed for men and women combined and separately (24, 25). Furthermore, we made separate analyses for workers in the public sector, private companies, and five different industries (the five industries with most observations in our regression sample).

For all calculations, we used SAS 9.4 and Stata 18.

### Sensitivity analyses

SA history strongly predicts future SA and may be associated with workers’ perception of their working conditions (26–28). In sensitivity analyses, we therefore repeated the main analyses while adding self-reported days of SA within the last year as a covariate. The sensitivity analyses were performed using observations from the 2014 to 2018 waves only, since they were the waves for which self-reported information on days of SA within the last year was available. It was not possible to use information on workers’ SA history from the SA register due to the way SA information is collected (not all workplaces report information on their workers’ SA, and the workplaces reporting change from year to year).

Among the observations in the regression sample, 2740 (4.0%) had missing values for the PSWE factors, and 8335 (12.3%) had missing values for the PSWE factors or the covariates. To avoid losing statistical power, we chose not to exclude observations with missing values on the explanatory variables. Instead, for each explanatory variable with missing data, that were all categorical, we created an extra category to hold the cases with missing data. Such a missing-group method can produce biased regression estimates (29). As a sensitivity check, we performed analyses excluding observations with missing values on PSWE factors, and complete-case analyses excluding all observations with missing values on explanatory variables.

## Results

Supplementary tables S2–S7 provide descriptive statistics on the explanatory variables, ie, the PSWE factors and the covariates.

Table 1 shows that in the source population the average annual SA per worker was 52 hours and the average annual costs of SA and healthcare use per worker were €1706 and €1539, respectively. Costs were higher for women than men. The average annual SA costs per worker was €2053 for women and €1367 for men, and the average annual costs of overall healthcare use per worker was €1780 for women and €1308 for men. Low-wage workers experienced more SA than high-wage workers. Therefore, the average annual SA costs per worker was lower than the product of the average annual hours of SA per worker and the average hourly wage rate.

**Table 1:** Means of outcome variables in the source population ^a^

	All	Men	Women
Annual hours of sickness absence ^b^	52.0	38.6	66.1
Hourly wage rate (euros)	35.4	38.6	32.1
Annual costs of sickness absence (euros) ^b^	1706	1367	2053
Annual costs of prescription drug use (euros) ^b^	161	157	177
Annual costs of primary health care use (euros) ^b^	279	216	341
Annual costs of hospital treatment (euros) ^b^	1056	849	1245
Total annual health care costs (euros) ^b^	1539 ^c^	1308 ^c^	1780 ^c^

^a^ Excludes workers in private companies with <10 full-time workers, workers in agriculture, forestry or fishing, and workers in subsidized jobs for disabled workers. Hourly wage rates and all costs are in 2023 euros. ^b^ Calculated from predictions of a hurdle model including the full set of explanatory variables listed in supplementary appendix 2. ^c^ The sum of the means of annual costs of prescription drug use, primary healthcare use and hospital treatment does not exactly add up to the mean of total annual health care costs because the different mean values were calculated from non-linear models.

### Economic gains from hypothetical improvements of the general psychosocial work environment

**Figure 2:**
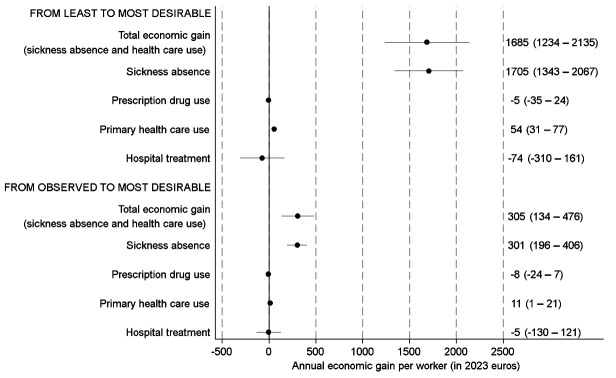
Estimates with 95% confidence intervals for annual economic gains per worker from two hypothetical improvements of the general psychosocial work environment. Parametric g-formula analyses with adjustment for sociodemographic characteristics, job characteristics, physical work environment characteristics, health status and health behaviors.

Figure 2 presents the estimated annual economic gains per worker from hypothetical interventions changing all PSWE factors from least to most desirable (upper part of figure) and from observed to most desirable (lower part of figure).

When we estimated the effects of a hypothetical improvement that changed the general PSWE from the least to the most desirable, we found an annual economic gain of €1685 (95% CI €1234–2135) per worker. When we estimated the effects of a hypothetical improvement that changed the general PSWE from the observed to the most desirable, the economic gain was €305 (95% CI €134–476).

In both analyses, the economic gain was overwhelmingly driven by savings in SA. Savings in healthcare use contributed only marginally (or not at all) to economic gain (figure 2).

Supplementary figure S1 shows the estimates stratified by sex. Hypothetical PSWE improvements were associated with economic gain among both women and men. However, the annual economic gain per worker was considerably higher among women than among men, both for improvements from the least to the most desirable [€2321 (women) and €943 (men), respectively] and from the observed to the most desirable (€505 (women) and €128 (men), respectively).

Supplementary table S8 provides separate estimates for different sectors and industries. The estimated economic gains from hypothetical PSWE improvements (from least to most desirable and from observed to most desirable) were larger for workers in the public sector than workers in private companies. Partly due to a smaller number of observations in the industry-specific analyses, differences between the estimated gains in the five selected industries were generally statistically insignificant.

### Economic gains from hypothetical improvements of specific psychosocial work environment factors

**Figure 3:**
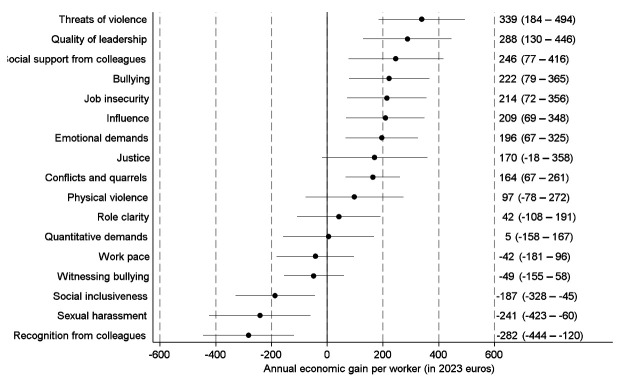
Estimates with 95% confidence intervals for annual economic gains per worker from hypothetical improvements (least to most desirable) of specific psychosocial work environment factors. Parametric g-formula analyses with adjustment for sociodemographic characteristics, job characteristics, physical work environment characteristics, health status and health behaviors.

**Figure 4:**
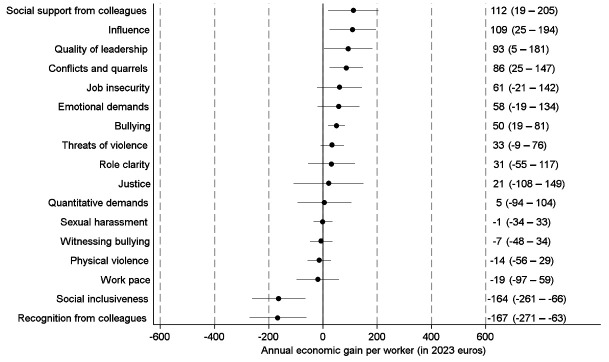
Estimates with 95% confidence intervals for annual economic gains per worker from hypothetical improvements (observed to most desirable) of specific psychosocial work environment factors. Parametric g-formula analyses with adjustment for sociodemographic characteristics, job characteristics, physical work environment characteristics, health status and health behaviors.

Figure 3 shows the economic effects of hypothetical improvements of specific PSWE factors from the least to the most desirable. We observed the strongest economic gains for elimination of threats of violence and for improvements of quality of leadership and social support from colleagues. When we analyzed the economic effects of improvements from the observed to the most desirable situation, we observed the strongest economic gains for improvements in social support, influence at work and quality of leadership (figure 4).

Improvements in recognition from colleagues and in social inclusiveness were associated with economic losses both for improvements from least to most desirable and improvements from observed to most desirable. In addition, elimination of sexual harassment was associated with an economic loss in the analyses of improvements from least to most desirable.

Supplementary Appendix 4 provides separate estimates for SA and healthcare use and for women and men (figures S2–S17), and supplementary Appendix 5 shows separate estimates for the public sector, private companies, and selected industries (figures S18–S61).

### Sensitivity analyses

When including self-reported days of SA within the last year in the set of covariates, the economic gains from hypothetically improving the general PSWE became weaker. The estimated annual economic gains per worker from improving the general PSWE from the least to the most desirable and from the observed to the most desirable were reduced to €1193 and €201, respectively (supplementary figure S62). Furthermore, with the inclusion of days of SA within the last year, we no longer found economic losses from improvements in recognition from colleagues and from improvements from least to most desirable in social inclusiveness (supplementary figures S63–S64).

Excluding observations with missing values on explanatory variables from the analyses did not change the results substantially (supplementary table S9 and figures S76–S87).

## Discussion

In this large prospective cohort study, using data from a nationally representative survey of Danish workers, we estimated economic effects of hypothetical improvements of the general PSWE and of specific PSWE factors.

We found substantial economic gains from hypothetically improving the general PSWE. The estimated annual economic gains per worker from improving the general PSWE from the least to the most desirable and from the observed to the most desirable were €1685 and €305, respectively. The gains were largely driven by savings in costs related to SA, and they were higher for women than men and for public sector compared to private sector workers.

When analyzing the economic effects of hypothetical improvements of specific PSWE factors, we found the largest economic gains for improvements from least to most desirable for elimination of threats of violence and improvements in quality of leadership and social support from colleagues. For improvements from observed to most desirable, we found the largest economic gains for improvements in social support from colleagues, influence at work and quality of leadership.

In contrast, elimination of sexual harassment was associated with an economic loss in the analyses of improvements from least to most desirable. Improvements in recognition from colleagues and social inclusiveness were also associated with economic losses in the main analyses, but these associations largely disappeared in our sensitivity analyses.

### Comparison with previous studies

To our knowledge, this is the first study applying the parametric g-formula to estimate economic effects of hypothetical improvements in the general PSWE and in several different specific PSWE factors, making direct comparisons of our results with previous findings difficult.

A limited number of cost-of-illness studies have estimated the costs associated with different PSWE risks, such as job strain, bullying and work-related violence (30–32). The estimated costs per worker of specific psychosocial risks vary a lot across studies due to, among other things, differences in methodologies and statistical techniques, differences in included types of costs and differences between different countries’ labor market structures and welfare systems.

Our estimates of the potential costs savings from hypothetical improvements of the observed PSWE are on the low end when compared to previous Danish studies on the effects of PSWE risks (16, 33–35).

For example, a study investigating the association between work-related psychosocial and physical risks and SA reported that such risks explained 28% of Danish workers’ SA (33). Assuming the average annual costs of SA per worker were €1706 (table 1), a simple calculation implies that the combined costs of psychosocial and physical risks were around €478 (0.28×€1706) per worker. The study did not provide estimates of the separate effects of the psychosocial and physical risks, but both types of risks contributed to SA. Therefore, the contribution of psychosocial risks to annual SA costs per worker were substantially smaller than €478 and closer to our estimate of the economic gain from improving the general PSWE from the observed to the most desirable (€305).

Another Danish study, which included costs of SA, disability pensioning, early death and healthcare use, estimated the annual costs of job strain (high quantitative demands and low influence at work) to be around €180–1080 per person in the labor force (with costs converted to 2023 euros) (34).

Furthermore, two Danish studies have estimated the costs of work-related stress, which to a large extent may be attributed to poor psychosocial working conditions. Pedersen et al (16) reported that the annual costs of work absenteeism (SA and unemployment) associated with work-related stress was €950 per worker, of which €620 related to SA. The Economic Council of the Labor Movement estimated the annual costs of work-related stress (coming from reductions in working hours and increases in SA) to be €3200 per worker (35).

The larger potential economic gains from hypothetical improvements of the general PSWE for women than men are in line with previous research showing that women have more SA (36, 37).

That improvements in social inclusiveness and recognition from colleagues and the elimination of sexual harassment were associated with economic losses was surprising. With regard to inclusiveness and recognition, one could speculate that SA is more socially acceptable in workplaces with a high degree of social inclusiveness and collegial recognition and that this association may explain the unexpected result.

It seems, however, implausible that elimination of sexual harassment would be associated with economic loss. Previous studies on the relationship between various forms of sexual harassment and long-term SA reported that sexual harassment was associated with an increased risk of long-term SA (38, 39). One explanation for our results regarding sexual harassment could be that the empirical model that we used was not sufficiently rich to capture the true relationship between the PSWE factors and the outcome variables. The estimated effect of sexual harassment could be biased in the direction of finding that sexual harassment decreases the risk of SA and healthcare use if the effect from simultaneously improving several PSWE factors is smaller than the sum of the effects from separate improvements in each of these factors. This is because, in our sample, workers exposed to sexual harassment were significantly more likely to experience several other unfavorable PSWE aspects than those not exposed to sexual harassment.

In our study, the ordering of PSWE factors by the potential reductions in costs of SA achievable from hypothetical improvements of these factors were different from the ordering in Mathisen et al (13) of similar PSWE factors by the potential reductions in SA. For example, we found improvements in quality of leadership to be one of the PSWE factors with the largest potential to reduce costs of SA (supplementary figures S2–S3), whereas Mathisen et al found improvements in this factor to be among the PSWE factors with the lowest potential to reduce SA. In contrast, improvements in influence at work were among the PSWE improvements with the most favorable effects in both our study and in Mathisen et al. A detailed comparison of our results with Mathisen et al is difficult. Among other things, we estimated the economic effects of hypothetical PSWE improvements whereas Mathisen et al estimated the effects on hours of SA. Our study population is a sample of all workers, whereas Mathisen et al restricted their sample to public hospital workers. Finally, we examined working conditions that only partly overlapped with the working conditions examined by Mathisen et al.

### Strengths and limitations

The study has some major advantages: we analyzed a large nationwide cohort of more than 70 000 Danish workers from different industries, which increases statistical power and external validity. We had access to detailed self-reported data on a broad range of psychosocial working conditions.

We applied the novel parametric g-formula, allowing us to estimate the potential economic gains associated with hypothetical improvements in the general PSWE and across 17 PSWE factors, while controlling for a wide array of potential confounders. Moreover, by pairwise comparison of hypothetical PSWE scenarios — the least to the most desirable, and the observed to the most desirable — we provided a nuanced understanding of the potential economic effects.

The study also has limitations. When estimating the economic gains from hypothetical improvements of the PSWE, we restricted attention to costs of workers’ SA and healthcare use, which we could measure in registers. However, we expect a poor PSWE to have other costs. The PSWE can affect productivity at the workplace not only through SA, but also through workers’ work motivation, worker turnover and presenteeism (working while sick) (12, 18). Previous research indicates that productivity losses due to presenteeism might likely be larger than those due to SA (30). Additionally, an adverse PSWE can impose costs on workers in the form of reduced quality of life and reduced ability to fulfil domestic and leisure roles (18). Furthermore, exposure to a poor PSWE can be associated with higher risk of unemployment and early labor market exit (40–42). By excluding relevant costs of a poor PSWE, we are likely to underestimate the cost reductions from PSWE improvements.

The response rate across the four WEHD waves was 55.9%, which can be considered satisfactory compared to other large-scale surveys conducted in random samples of a national workforce in the Nordic countries (43). We have previously analyzed in detail the demographic characteristics of the non-responders in the 2012 WEHD wave. Non-responders were more often men, of younger age, and with lower educational attainment (14). This selective non-response may have introduced selection bias into our analyses. To address this potential bias, we adjusted our analyses for a wide range of covariates – including sex, age, and education – and used appropriate sample weights.

Residual confounding is an inherent concern in observational studies, and we therefore adjusted for a wide range of possible confounders. However, adjusting for many confounders might also introduce over-adjustment, potentially accounting for mediators and leading to more conservative estimates of the economic gains from improving the PSWE.

Several prior studies have linked various adverse psychosocial working conditions to SA. Given the interrelated nature of these conditions, interventions targeting specific aspects of the PSWE may indirectly affect other PSWE aspects, potentially broadening the impact on SA and economic effects. Moreover, it should be noted that the estimated potential economic gains presented are from hypothetical scenarios where PSWE factors would be improved to the most favorable level. These estimates represent upper bounds on the potential economic gains achievable through PSWE improvements, in contrast to what may be realistic in real-life settings where often smaller PSWE improvements are observed (4). Finally, some PSWE improvements might inadvertently reduce productivity. For example, reducing work pace or quantitative demands might lower worker output. Thus, PSWE improvements do not always result in economic gains, especially when considering the direct costs of implementing these changes.

### Implications for real interventions

The estimated economic gains from hypothetical interventions presented in this study are economic gains from a societal perspective that does not consider the direct interventions costs. These economic gains can be used as an input to obtain well-informed estimates of the net economic societal gains of actual workplace interventions (for given intervention costs) or estimates of the maximum intervention costs that can be spent if interventions should not result in net economic societal losses.

For example, assume that a nationwide intervention aiming to improve quality of leadership is being considered for implementation. We estimated an annual economic gain of €93 per worker from improving quality of leadership from observed to most desirable. Therefore, if the intervention has maximum efficiency, actually improving quality of leadership to most desirable for all workers, the annual net economic gain per worker from the intervention would be €93 minus the intervention costs per worker. If the intervention only brings 10% of workers with “least desirable” or “medium” quality of leadership to “most desirable” quality of leadership, the annual net economic gain per worker would be €9.3 minus the intervention costs per worker.

If estimates of other economic gains from the intervention than reductions in costs of SA and healthcare use exist (eg, reduced turnover or presenteeism costs), these economic gains could of course be included when estimating the net economic gains.

In cases, where the aim is to estimate the net economic gains of an intervention for a specific group of workers, our estimates should be adjusted to account for the degree to which the workers are exposed to unfavorable PSWE aspects and the costs of SA and healthcare use before the intervention (measuring the potential for cost reductions). The larger the exposure and initial costs of SA and healthcare use, the larger the expected net economic gains from the intervention.

Finally, it is worth mentioning that the economic gains from a workplace intervention is smaller from an employer’s perspective than from the societal perspective. This is so because the reductions in the costs of healthcare use and part of the reductions in societal costs of SA (equal to the gains in production value) does not fall into the hands of the employer. Employers will generally have to pay higher salaries when SA decreases, because they pay reduced salaries while workers are sick or because they receive partial reimbursement for salaries paid to workers with >30 days of SA. Consequently, an employer’s economic gain from reductions in SA is the increase in production value minus the increase in salaries net of reimbursements. A policy implication is that public subsidies may sometimes be relevant to increase the incentives to implement interventions providing net economic societal gains.

### Concluding remarks

In summary, we found that interventions improving the PSWE could potentially lead to economic gains, in particular improvements for female workers and workers in public sector workplaces. When considering individual PSWE factors, interventions towards improving the work environment with regard to threats of violence, quality of leadership, social support from colleagues and influence at work held the largest economic potential.

## Supplementary material

Supplementary material
