# Supplementary material for: Economic gains from hypothetical improvements in the psychosocial work environment: A cohort study of 71 207 workers in Denmark
Source: Scand J Work Environ Health. 2025 Oct 30;51(6):472–82. doi: 10.5271/sjweh.4244 (PMC12590404; doi:10.5271/sjweh.4244)
Supplement: Supplementary material [file SJWEH-51-472-S001.pdf]

Economic gains from hypothetical improvements in the psychosocial work environment: A cohort study of 71 207 workers in Denmark[1]

by Brian Krogh Graversen, PhD,[2] Kristian Schultz Hansen, PhD, Reiner Rugulies, PhD, Jeppe Karl Sørensen, PhD, Ann Dyreborg Larsen, PhD

1. Supplementary material
2. Correspondence to: Brian Krogh Graversen, PhD, National Research Centre for the Working Environment, Lersø Parkallé 105, DK-2100 Copenhagen, Denmark. [E-mail: bkg@nfa.dk]

Contents

Appendix 1. The psychosocial work environment scales **2**

- Table S1

Appendix 2. Descriptive statistics for the explanatory variables **3**

- Tables S2–S7

Appendix 3. The algorithm for estimating economic effects of hypothetical improvements of the psychosocial work environment **15**

Appendix 4. Estimates stratified by sex and cost types **17**

- Figures S1–S17

Appendix 5. Estimates for different sectors and industries **26**

- Table S8
- Figures S18–S61

Appendix 6. Sensitivity analyses regarding sickness absence history **50**

- Figures S62–S75

Appendix 7. Sensitivity analyses regarding observations with missing values **58**

- Table S9
- Figures S76–S87

References **65**

Appendix 1. The psychosocial work environment scales

Table S1. Psychosocial work environment scales and included questionnaire items

Domain	Scale	Question/item
Demands at work	1. Quantitative demands (CA=0.72)	1.1 How often do you feel that you have enough time for your work tasks?<br>1.2 How often do you have deadlines that are difficult to meet?<br>1.3 How often do you get unexpected work assignments that put you under time pressure?<br>1.4 How often are you available for work outside normal working hours?<br>1.5 How often is it necessary to work overtime?
	2. Work pace	2.1 How often is it necessary to maintain a high work pace?
	3. Emotional demands	3.1 How often are you emotionally affected by your work?
Work organization and job contents	4. Influence (CA=0.73)	4.1 How often do you have an influence on how you solve your work tasks?<br>4.2 How often do you have influence over when you solve your work tasks?
Interpersonal relations and leadership	5. Role clarity (CA=0.69)	5.1 How often do you get the information you need to do your job?<br>5.2 How often do you get the guidance and instruction you need to do your job?<br>5.3 How often do you know exactly what your work tasks are?<br>5.4 How often are opposing demands made on you in your work?
	6. Quality of leadership (CA=0.89)	6.1 How often does your immediate manager explain the company's goals to you so that you understand what they mean for your tasks?<br>6.2 How often do you have sufficient authority in relation to the responsibilities you have in your work?<br>6.3 How often does your immediate manager take the time to get involved in your professional development?<br>6.4 How often does your immediate manager involve you in the organization of your work?<br>6.5 How often does your immediate manager give you the necessary feedback for your work?<br>6.6 How often is your work recognized and appreciated by management?<br>6.7 How often do you get the help and support you need from your immediate manager?<br>6.8 How often can you trust the information that comes from management?
	7. Social support from colleagues (CA=0.77)	7.1 How often do you and your colleagues help each other to achieve the best possible result?<br>7.2 How often do you and your colleagues collaborate when problems requiring solutions arise?
	8. Recognition from colleagues	8.1 How often do you and your colleagues acknowledge each other at work?
Work-individual interface	9. Job insecurity (CA=0.64)	9.1 To what extent are you worried about becoming unemployed?[a]<br>9.2 To what extent are you concerned that you will be transferred to another work assignment against your will?[a]
Values at the workplace	10. Justice (CA=0.72)	10.1 How often are all workers affected by a given decision consulted?<br>10.2 How often are all workers treated fairly in the workplace?
	11. Social inclusiveness	11.1 How often are workers with less physical strength (e.g. elderly or sick) taken into account at your workplace?
Offensive behavior	12. Conflicts and quarrels	12.1 Within the last 12 months, have you had arguments or conflicts with someone at your workplace?[b]
	13. Bullying	13.1 Within the last 12 months, have you been exposed to bullying at your workplace?[b]
	14. Witnessing bullying	14.1 Within the last 12 months, have you witnessed someone at your workplace being bullied?[b]
	15. Physical violence	15.1 Within the last 12 months, have you been exposed to physical violence at your workplace?[b]
	16. Threats of violence	16.1 Within the last 12 months, have you been exposed to threats of violence at your workplace?[b]
	17. Sexual harassment	17.1 Within the last 12 months, have you been exposed to sexual harassment at your workplace?[b]

Notes: CA is Cronbach's alpha. Unless otherwise stated, item response options were: Always; Often; Sometimes; Seldom; Never/hardly ever.

[a] Item response options were: To a very large extent; To a large extent; Somewhat; To a small extent; To a very small extent.

[b] Item response options were: Yes, daily; Yes, weekly; Yes, monthly; Yes, a few times; No.

Appendix 2. Descriptive statistics for the explanatory variables

To provide an overview of the explanatory variables and their descriptive statistics, we divided our explanatory variables into six groups: A, B, C, D, E and F.

Variable group A includes the 17 variables describing different aspects of the psychosocial work environment, a categorical variable for survey wave and a fourth order polynomial of logarithm of the length of the period over which we measured hours of sickness absence and costs of health care use (length is less than or equal to one year). We included the last mentioned measurement period length because our outcome measures naturally increase with this length.

Variable group B includes variables describing various sociodemographic factors: an indicator for sex, a fourth order polynomial of age, an indicator for immigrant status (immigrant or not), a categorical variable for family type, an interaction of a categorical variable for age of the youngest child in the family with worker's sex and a categorical variable for highest educational attainment.

Variable group C includes variables providing information on job characteristics: categorical variables for work type, sector, industry, type of occupation, seniority at the workplace, actual weekly working hours, time of day working, commuting time to and from work, number of full-time workers at the local workplace and number of full-time workers in the overall company.

Variable group D includes variables describing aspects of the physical work environment: categorical variables for exposure to various physical work conditions and physical strenuousness of the work.

Variable group E includes variables characterizing workers' health status: a categorical variable for body mass index and 11 separate categorical variables describing whether workers were in treatment at questionnaire completion or had been in treatment during the previous year for depression, asthma, diabetes, atherosclerosis or myocardial infarction (blood clot in the heart), ischemic stroke (blood clot in the brain), cancer, hearing loss, eczema, back disease, migraine or another long-term illness.

Variable group F includes variables giving information on some health behaviors: categorical variables for smoking behavior, alcohol consumption and the extent and type of exercise.

Tables S2 to S7 provide descriptive statistics for the variables in each of the groups A to F.

Table S2. Descriptive statistics for the explanatory variables included in variable group A (psychosocial work environment factors)

		Source population[a]	Study sample, N=71 207 (102 379 obs.)	Regression sample, N=48 434 (67 780 obs.)		
		(1)	(2)	(3)	(2)-(1)	(3)-(1)
		% of pop.	% of N	% of N	%pt.	%pt.
Quantitative demands	Least desirable	24.1	23.7	23.6	-0.3	-0.5
	Medium	50.2	51.3	51.9	1.1	1.7
	Most desirable	23.2	23.0	22.8	-0.2	-0.4
	Unknown	2.6	2.0	1.8	-0.6	-0.8
Work pace	Least desirable	21.5	20.6	21.3	-0.8	-0.2
	Medium	45.8	46.9	47.7	1.1	1.9
	Most desirable	30.1	30.4	29.1	0.4	-0.9
	Unknown	2.6	2.0	1.8	-0.6	-0.8
Emotional demands	Least desirable	24.0	24.6	26.4	0.6	2.4
	Medium	38.1	39.7	41.7	1.6	3.6
	Most desirable	36.0	34.2	30.5	-1.8	-5.4
	Unknown	2.0	1.5	1.3	-0.4	-0.6
Influence	Least desirable	25.3	24.4	25.3	-0.9	0.0
	Medium	47.8	48.8	49.8	1.0	2.0
	Most desirable	25.1	25.4	23.7	0.3	-1.4
	Unknown	1.8	1.4	1.2	-0.4	-0.6
Role clarity	Least desirable	24.1	24.0	23.8	0.0	-0.2
	Medium	46.9	47.0	47.9	0.1	1.1
	Most desirable	27.2	27.6	27.0	0.3	-0.3
	Unknown	1.9	1.5	1.3	-0.4	-0.6
Quality of leadership	Least desirable	23.9	24.2	23.6	0.3	-0.3
	Medium	48.0	48.2	49.3	0.2	1.3
	Most desirable	25.9	25.6	25.8	-0.3	-0.1
	No superior	1.3	1.4	0.9	0.0	-0.5
	Unknown	0.8	0.6	0.5	-0.2	-0.3
Social support from colleagues	Least desirable	20.1	20.3	20.1	0.2	-0.1
	Medium	53.4	54.6	55.6	1.2	2.3
	Most desirable	24.8	23.8	23.2	-1.0	-1.6
	Unknown	1.7	1.3	1.1	-0.4	-0.6
Recognition from colleagues	Least desirable	21.4	21.5	21.6	0.1	0.2
	Medium	52.4	53.4	54.4	1.0	2.0
	Most desirable	24.4	23.6	22.8	-0.8	-1.6
	Unknown	1.9	1.5	1.3	-0.4	-0.6
Job insecurity	Least desirable	20.0	19.7	20.7	-0.3	0.7
	Medium	55.4	54.9	54.9	-0.5	-0.5
	Most desirable	24.3	25.2	24.2	0.9	-0.1
	Unknown	0.3	0.2	0.2	-0.1	-0.1
Justice	Least desirable	19.5	19.9	19.9	0.4	0.5
	Medium	63.8	64.5	65.7	0.7	1.9
	Most desirable	15.0	14.3	13.2	-0.7	-1.8
	Unknown	1.7	1.4	1.2	-0.4	-0.6
Social inclusiveness	Least desirable	33.5	34.2	34.1	0.8	0.6
	Medium	40.5	41.3	42.5	0.8	2.1
	Most desirable	24.1	23.0	22.1	-1.2	-2.0
	Unknown	1.9	1.5	1.3	-0.4	-0.6
Conflicts and quarrels	Least desirable	54.2	54.8	54.7	0.6	0.4
	Most desirable	42.7	42.7	43.1	0.0	0.4
	Unknown	3.1	2.5	2.3	-0.6	-0.8
Bullying	Least desirable	11.1	11.2	11.3	0.1	0.2
	Most desirable	85.9	86.3	86.5	0.5	0.7
	Unknown	3.0	2.4	2.2	-0.6	-0.8

Witnessing bullying	Least desirable	27.5	28.2	28.8	0.8	1.4
	Most desirable	69.2	69.1	68.7	-0.2	-0.6
	Unknown	3.3	2.7	2.5	-0.6	-0.8
Physical violence	Least desirable	6.2	6.5	8.5	0.4	2.3
	Most desirable	90.7	91.0	89.2	0.3	-1.5
	Unknown	3.1	2.5	2.3	-0.6	-0.9
Threats of violence	Least desirable	9.0	9.4	11.7	0.4	2.7
	Most desirable	87.9	88.1	86.1	0.2	-1.8
	Unknown	3.1	2.5	2.3	-0.6	-0.9
Sexual harassment	Least desirable	3.3	3.0	3.3	-0.3	0.0
	Most desirable	93.6	94.5	94.4	1.0	0.8
	Unknown	3.2	2.5	2.3	-0.7	-0.9
Survey wave	2012	25.0	20.2	20.4	-4.8	-4.6
	2014	25.0	22.3	22.7	-2.7	-2.4
	2016	25.0	26.0	25.1	1.0	0.1
	2018	25.0	31.5	31.9	6.5	6.9
Duration of outcome measurement period[b]	0–0.19 year	-	-	4.2	-	-
	0.2–0.39 year	-	-	3.1	-	-
	0.4–0.59 year	-	-	5.7	-	-
	0.6–0.79 year	-	-	11.4	-	-
	0.8–0.99 year	-	-	3.9	-	-
	1 year	-	-	71.7	-	-

Notes: The variable *Duration of outcome measurement period* was constructed using information from administrative registers. All other variables were constructed using information from the WEHD survey.

[a] We obtained the figures for the source population by applying the sample weights associated with observations in the study sample.

[b] The variable *Duration of outcome measurement period* is missing for workers for whom information on sickness absence from their main job (the job with most working hours at questionnaire completion) is missing. The value of the variable equals one year if, one year after questionnaire completion, a worker was still employed in the main job identified at questionnaire completion. Otherwise, the variable equals the number of days from questionnaire completion until the job ended or information on sickness absence from the job was no longer available. Notice that *Duration of outcome measurement period* was included in the regression models as a fourth order polynomial and not as a categorical variable.

Table S3. Descriptive statistics for the explanatory variables included in variable group B (sociodemographic factors)

		Source population[a]	Study sample, N=71 207 (102 379 obs.)	Regression sample, N=48 434 (67 780 obs.)		
		(1)	(2)	(3)	(2)-(1)	(3)-(1)
		% of pop.	% of N	% of N	%pt.	%pt.
Sex	Man	50.7	45.7	39.4	-5.0	-11.3
	Woman	49.3	54.3	60.6	5.0	11.3
Age[b]	18–24 years	8.4	3.9	2.8	-4.5	-5.7
	25–34 years	19.3	12.0	11.5	-7.3	-7.8
	35–44 years	25.8	22.9	23.0	-2.9	-2.8
	45–54 years	27.9	32.9	33.2	5.0	5.3
	55+ years	18.6	28.3	29.6	9.7	11.0
Immigrant	No	92.9	94.1	94.3	1.3	1.4
	Yes	7.1	5.9	5.7	-1.3	-1.4
Family type	Single	27.5	23.6	22.8	-4.0	-4.7
	Married	54.0	60.5	61.7	6.5	7.7
	Cohabitating	18.5	15.9	15.5	-2.5	-3.0
Youngest child[c]	No children below 18 years	58.0	59.8	59.0	1.8	1.1
	0–2 years	9.4	7.1	7.2	-2.3	-2.2
	3–5 years	7.3	6.2	6.4	-1.1	-0.9
	6–17 years	25.3	26.9	27.4	1.6	2.1
Highest completed education	Primary education	12.5	11.4	9.8	-1.2	-2.7
	Upper secondary school	8.7	6.3	5.5	-2.5	-3.3
	Vocational education	34.6	35.1	32.2	0.5	-2.4
	Short further education	6.6	6.6	6.1	0.0	-0.5
	Long further education	20.6	23.9	28.3	3.2	7.7
	Bachelor's degree	2.3	1.8	1.5	-0.5	-0.8
	Master's degree	12.7	13.0	14.4	0.3	1.7
	PhD degree	1.4	1.4	1.8	0.1	0.5
	Unknown	0.6	0.6	0.5	0.0	-0.2

Notes: All variables in the table were constructed using information from administrative registers.

[a] We obtained the figures for the source population by applying the sample weights associated with observations in the study sample.

[b] Age was included in the regression models as a fourth order polynomial and not as a categorical variable.

[c] The regression models included an interaction of the categorical variable for age of the youngest child in the family with worker's sex.

Table S4. Descriptive statistics for the explanatory variables included in variable group C (job characteristics)

		Source population[a] (1)	Study sample, N=71 207 (102 379 obs.) (2)	Regression sample, N=48 434 (67 780 obs.) (3)	(2)-(1)	(3)-(1)
		% of pop.	% of N	% of N	%pt.	%pt.
Work type	Ordinary work	80.3	82.9	85.1	2.6	4.8
	Work with personnel responsibility	13.1	13.8	12.5	0.6	-0.6
	Student work, 8 hours or more per week	3.6	1.8	1.2	-1.8	-2.4
	Other	2.9	1.5	1.2	-1.4	-1.8
Sector	State	9.3	10.3	13.6	1.0	4.2
	Regions	6.3	7.1	9.9	0.7	3.6
	Municipalities	23.6	26.4	36.8	2.8	13.3
	Public companies	3.1	3.4	3.9	0.3	0.8
	Private companies	54.8	49.7	34.6	-5.1	-20.2
	Other	2.8	3.2	1.1	0.3	-1.7
Industry	Manufacturing; mining and quarrying	13.8	14.6	11.8	0.9	-2.0
	Electricity, gas and water supply; waste collection	1.0	1.3	1.2	0.3	0.2
	Construction	4.4	4.2	1.8	-0.2	-2.5
	Retail and wholesale	12.9	9.9	6.2	-3.0	-6.7
	Transport	5.0	4.5	3.7	-0.5	-1.3
	Hotels and restaurants	1.9	1.3	0.7	-0.5	-1.2
	Information and communication	3.7	3.6	2.5	-0.1	-1.2
	Finance, insurance, real estate	4.8	4.8	5.4	0.0	0.6
	Knowledge service	5.6	5.1	3.7	-0.4	-1.8
	Travel agencies and cleaning	4.6	3.9	3.5	-0.7	-1.2
	Public administration, defense and police	6.9	7.4	10.3	0.5	3.4
	Education	10.3	12.1	15.1	1.8	4.8
	Human health activities	7.0	8.0	10.9	1.0	3.8
	Social institutions[b]	14.9	16.0	20.9	1.1	6.1
	Culture and other services	3.4	3.4	2.3	0.0	-1.1
Type of occupation[c]	Armed forces occupations	0.9	0.7	1.0	-0.3	0.0
	Managers	4.8	5.0	4.4	0.3	-0.4
	Professionals	30.3	34.8	42.0	4.5	11.7
	Technicians and associate professionals	12.7	13.6	12.9	0.9	0.2
	Clerical support workers	9.1	9.1	8.7	0.1	-0.4
	Services and sales workers	17.5	14.8	16.5	-2.7	-0.9
	Craft and related trades workers	7.7	6.9	3.9	-0.8	-3.8
	Plant and machine operators, and assemblers	5.7	5.7	4.4	0.0	-1.3
	Elementary occupations	7.8	6.7	5.8	-1.1	-2.0
	Unknown	3.6	2.7	0.5	-0.9	-3.2
Seniority at workplace	Less than 3 months	3.0	2.2	1.8	-0.8	-1.2
	3—12 months	11.4	8.6	7.5	-2.9	-4.0
	1—3 years	18.8	15.1	13.9	-3.7	-4.9
	3—5 years	13.7	12.5	12.0	-1.2	-1.8
	5—10 years	19.9	20.4	20.6	0.5	0.7

	10+ years	32.9	41.0	44.2	8.1	11.2
	Unknown	0.2	0.2	0.2	-0.1	-0.1
Actual weekly working hours	0–29.9	7.4	5.8	5.1	-1.7	-2.4
	30–36.9	14.5	15.6	18.2	1.1	3.7
	37	35.9	36.7	36.9	0.8	1.0
	37.1–40.9	20.7	20.8	20.3	0.2	-0.3
	41+	20.5	20.2	18.7	-0.3	-1.8
	Unknown	1.1	0.9	0.9	-0.2	-0.3
Time of day working	Permanent day work	80.9	83.0	82.0	2.1	1.0
	Permanent evening work	3.4	3.0	3.3	-0.5	-0.2
	Permanent night work	1.4	1.3	1.4	-0.1	0.0
	Changing working hours with night work	6.5	5.8	6.2	-0.7	-0.3
	Changing working hours without night work	7.1	6.5	6.8	-0.6	-0.4
	Unknown	0.6	0.4	0.4	-0.2	-0.2
Commuting time to and from work	Less than 30 minutes	49.3	49.4	49.3	0.1	0.0
	30–60 minutes	36.2	36.1	36.3	0.0	0.1
	1–2 hours	11.6	11.7	11.8	0.1	0.2
	More than 2 hours	2.4	2.4	2.3	-0.1	-0.1
	Unknown	0.5	0.5	0.4	-0.1	-0.2
Full-time workers at workplace	0–9.9	6.3	5.4	4.2	-0.9	-2.2
	10–34.9	25.3	24.4	16.3	-0.9	-8.9
	35–99.9	24.9	26.5	26.0	1.6	1.1
	100–249.9	16.3	17.4	19.3	1.0	2.9
	250–499.9	8.8	8.7	10.9	0.0	2.1
	500+	18.5	17.6	23.4	-0.9	4.9
Full-time workers in overall company or institution	0–9.9	0.5	0.5	0.1	0.0	-0.5
	10–249.9	36.8	35.2	14.6	-1.6	-22.2
	250+	62.7	64.3	85.4	1.6	22.7

Notes: The variables *Work type*, *Seniority at workplace, Actual weekly working hours*, *Time of day working* and *Commuting time to and from work* were constructed using information from the WEHD survey. All other variables in the table were constructed using information from administrative registers.

[a] We obtained the figures for the source population by applying the sample weights associated with observations in the study sample.

[b] Social institutions include institutions providing residential care activities and social work activities without accommodation (e.g. nursing homes and day care institutions).

[c] Occupations were categorized according to the ISCO-08 classification (1).

Table S5. Descriptive statistics for the explanatory variables included in variable group D (physical work environment factors)

		Source population[a] (1)	Study sample, N=71 207 (102 379 obs.) (2)	Regression sample, N=48 434 (67 780 obs.) (3)	(2)-(1)	(3)-(1)
		% of pop.	% of N	% of N	%pt.	%pt.
Very loud noise	Almost all the time	2.1	1.9	1.8	-0.2	-0.3
	About 3/4 of the time	2.4	2.3	2.2	-0.2	-0.3
	About 1/2 of the time	4.1	3.8	3.7	-0.3	-0.5
	About 1/4 of the time	8.9	8.4	8.1	-0.5	-0.8
	Rarely	36.4	37.4	37.2	1.0	0.8
	Never	42.7	43.4	44.7	0.7	2.0
	Unknown	3.3	2.7	2.4	-0.7	-0.9
Disturbing noise	Almost all the time	8.9	9.2	9.8	0.4	0.9
	About 3/4 of the time	7.0	7.3	7.8	0.3	0.8
	About 1/2 of the time	11.9	12.0	12.5	0.1	0.7
	About 1/4 of the time	19.5	19.7	20.4	0.2	0.9
	Rarely	34.3	35.1	33.8	0.8	-0.5
	Never	15.2	14.0	13.3	-1.2	-1.9
	Unknown	3.3	2.7	2.4	-0.7	-0.9
Strong vibrations	Almost all the time	1.1	0.9	0.8	-0.2	-0.4
	About 3/4 of the time	1.0	0.9	0.7	-0.1	-0.3
	About 1/2 of the time	1.7	1.5	1.2	-0.2	-0.6
	About 1/4 of the time	3.6	3.1	2.4	-0.5	-1.2
	Rarely	19.1	18.7	16.6	-0.4	-2.5
	Never	70.1	72.2	75.9	2.0	5.8
	Unknown	3.4	2.7	2.5	-0.6	-0.9
Wet or damp hands	Almost all the time	3.3	2.9	2.9	-0.5	-0.4
	About 3/4 of the time	3.2	3.0	3.2	-0.2	0.0
	About 1/2 of the time	4.8	4.3	4.4	-0.4	-0.4
	About 1/4 of the time	9.8	9.4	9.6	-0.5	-0.2
	Rarely	24.0	23.9	22.9	-0.1	-1.2
	Never	51.5	53.8	54.5	2.4	3.1
	Unknown	3.4	2.7	2.5	-0.7	-0.9
Skin contact with chemicals	Almost all the time	2.7	2.4	2.6	-0.3	-0.1
	About 3/4 of the time	1.7	1.6	1.7	-0.2	-0.1
	About 1/2 of the time	2.7	2.4	2.5	-0.3	-0.2
	About 1/4 of the time	7.6	7.0	7.3	-0.6	-0.3
	Rarely	25.4	25.3	24.0	-0.1	-1.4
	Never	56.5	58.6	59.5	2.2	3.0
	Unknown	3.4	2.7	2.4	-0.7	-0.9
Sitting	Almost all the time	16.3	16.5	15.9	0.2	-0.4
	About 3/4 of the time	21.4	22.7	22.8	1.3	1.4
	About 1/2 of the time	16.7	17.8	19.1	1.2	2.4
	About 1/4 of the time	15.8	16.4	18.3	0.6	2.6
	Rarely	19.2	17.9	17.2	-1.3	-2.0
	Never	7.1	5.7	4.0	-1.3	-3.0
	Unknown	3.6	2.9	2.6	-0.7	-1.0
Walking or standing	Almost all the time	23.3	20.7	18.6	-2.6	-4.7
	About 3/4 of the time	14.2	14.6	15.9	0.4	1.7
	About 1/2 of the time	19.0	20.2	21.7	1.3	2.8
	About 1/4 of the time	24.4	25.7	26.0	1.4	1.6
	Rarely	14.9	15.2	14.7	0.3	-0.3
	Never	0.7	0.6	0.5	-0.1	-0.2
	Unknown	3.6	2.9	2.7	-0.7	-0.9
Twisted back or forward bending position	Almost all the time	3.8	3.4	3.0	-0.4	-0.8

	About 3/4 of the time	4.1	3.7	3.3	-0.4	-0.8
	About 1/2 of the time	6.6	5.9	5.5	-0.7	-1.1
	About 1/4 of the time	12.7	12.0	11.8	-0.7	-0.9
	Rarely	34.2	34.7	35.2	0.6	1.0
	Never	35.0	37.4	38.5	2.4	3.5
	Unknown	3.7	3.0	2.7	-0.7	-1.0
Arms raised at shoulder level or above	Almost all the time	1.4	1.2	1.0	-0.2	-0.4
	About 3/4 of the time	1.8	1.6	1.3	-0.3	-0.5
	About 1/2 of the time	3.5	3.0	2.6	-0.5	-0.9
	About 1/4 of the time	10.6	9.5	8.6	-1.1	-2.0
	Rarely	40.0	40.5	41.4	0.5	1.4
	Never	39.1	41.4	42.4	2.3	3.3
	Unknown	3.6	2.9	2.7	-0.7	-0.9
Same arm movements many times a minute	Almost all the time	5.2	4.6	4.0	-0.6	-1.2
	About 3/4 of the time	3.6	3.2	2.9	-0.3	-0.7
	About 1/2 of the time	4.7	4.2	3.5	-0.5	-1.2
	About 1/4 of the time	6.7	5.9	5.2	-0.7	-1.5
	Rarely	22.3	22.1	21.3	-0.2	-1.0
	Never	54.1	57.1	60.6	3.0	6.5
	Unknown	3.6	2.9	2.7	-0.7	-1.0
Squatting or kneeling	Almost all the time	0.9	0.7	0.5	-0.2	-0.3
	About 3/4 of the time	1.3	1.1	1.0	-0.2	-0.4
	About 1/2 of the time	3.0	2.5	2.0	-0.6	-1.0
	About 1/4 of the time	10.6	9.7	9.4	-0.9	-1.3
	Rarely	29.9	29.9	30.6	0.0	0.7
	Never	50.8	53.3	53.9	2.6	3.2
	Unknown	3.6	2.9	2.6	-0.7	-0.9
Pushing or pulling	Almost all the time	1.9	1.5	1.4	-0.4	-0.5
	About 3/4 of the time	1.9	1.6	1.5	-0.2	-0.3
	About 1/2 of the time	3.8	3.2	3.0	-0.6	-0.8
	About 1/4 of the time	12.5	11.5	11.3	-1.0	-1.2
	Rarely	29.1	29.4	29.2	0.3	0.1
	Never	47.2	49.8	50.9	2.6	3.7
	Unknown	3.7	3.0	2.7	-0.7	-0.9
Lifting or carrying	Almost all the time	3.5	2.7	2.2	-0.8	-1.2
	About 3/4 of the time	3.2	2.7	2.3	-0.6	-0.9
	About 1/2 of the time	6.2	5.3	4.5	-0.9	-1.7
	About 1/4 of the time	15.5	14.6	13.7	-0.9	-1.8
	Rarely	32.3	34.5	35.7	2.2	3.4
	Never	35.6	37.2	38.8	1.6	3.2
	Unknown	3.8	3.1	2.9	-0.7	-0.9
Typical weight of things being lifted or carried	No lifting or carrying	35.6	37.2	38.8	1.6	3.2
	Below 5 kg	22.9	23.9	24.9	1.0	2.0
	5–15 kg	25.7	24.7	23.2	-1.0	-2.5
	16–29 kg	7.4	6.7	5.5	-0.7	-2.0
	30+ kg	4.3	4.1	4.5	-0.2	0.2
	Unknown	4.1	3.5	3.3	-0.7	-0.9
Typical lifting is strenuous	No lifting or carrying	35.6	37.2	38.8	1.6	3.2
	Always	1.5	1.3	1.3	-0.2	-0.2
	Often	7.4	6.7	6.3	-0.7	-1.1
	Sometimes	20.2	19.1	18.3	-1.1	-1.9
	Rarely	22.1	22.6	22.5	0.5	0.3
	Never	9.3	9.8	9.9	0.5	0.6
	Unknown	3.9	3.3	3.0	-0.7	-0.9
Uses aids when necessary when lifting or moving things or people	No lifting or carrying	35.6	37.2	38.8	1.6	3.2
	Always	9.8	9.9	10.3	0.1	0.4
	Often	16.2	15.7	15.0	-0.5	-1.1

	Sometimes	11.4	10.7	9.9	-0.7	-1.5
	Rarely	8.7	8.5	8.1	-0.2	-0.6
	Never	14.4	14.8	14.9	0.4	0.5
	Unknown	4.0	3.3	3.0	-0.7	-0.9
Lifts or moves things or people alone when there should be two to do it	No lifting or carrying	35.6	37.2	38.8	1.6	3.2
	Always	1.1	0.9	0.9	-0.1	-0.2
	Often	6.4	5.4	4.7	-1.0	-1.7
	Sometimes	14.8	13.7	12.8	-1.1	-2.0
	Rarely	19.4	19.7	19.4	0.3	0.0
	Never	18.9	19.9	20.5	1.0	1.6
	Unknown	3.9	3.3	3.0	-0.7	-0.9
Physical strenuousness of work	0 (Not strenuous at all)	25.2	27.2	27.7	2.0	2.5
	1	11.0	11.4	11.8	0.4	0.8
	2	10.2	10.7	11.1	0.5	0.9
	3	8.8	9.1	9.4	0.3	0.6
	4	6.0	6.1	6.2	0.1	0.3
	5	8.6	8.5	8.5	-0.1	-0.1
	6	7.2	6.5	6.3	-0.7	-0.9
	7	9.2	8.3	7.9	-0.9	-1.3
	8	7.2	6.6	6.1	-0.6	-1.2
	9	2.0	1.7	1.6	-0.3	-0.4
	10 (Maximum)	1.3	1.2	1.1	-0.1	-0.3
	Unknown	3.3	2.7	2.4	-0.7	-0.9

Notes: All variables in the table were constructed using information from the WEHD survey.

[a] We obtained the figures for the source population by applying the sample weights associated with observations in the study sample.

Table S6. Descriptive statistics for the explanatory variables included in variable group E (health characteristics)

		Source population[a]	Study sample, N=71 207 (102 379 obs.)	Regression sample, N=48 434 (67 780 obs.)		
		(1)	(2)	(3)	(2)-(1)	(3)-(1)
		% of pop.	% of N	% of N	%pt.	%pt.
Body mass index	0–18.49 (underweight)	1.2	1.1	1.1	-0.2	-0.2
	18.5–24.99 (normal weight)	47.7	46.4	47.7	-1.3	0.0
	25–29.99 (overweight)	32.8	34.2	33.2	1.3	0.3
	30–34.99 (obesity, class 1)	10.0	10.6	10.5	0.7	0.5
	35–39.99 (obesity, class 2)	2.6	2.7	2.8	0.1	0.1
	40+ (obesity, class 3)	0.9	1.0	1.0	0.0	0.1
	Unknown	4.8	4.1	3.8	-0.7	-0.9
Depression	Yes	4.9	4.6	4.6	-0.2	-0.2
	No	90.0	90.9	91.3	0.9	1.2
	Unknown	5.1	4.4	4.1	-0.7	-1.0
Asthma	Yes	4.9	5.1	5.2	0.2	0.2
	No	89.9	90.4	90.6	0.6	0.8
	Unknown	5.2	4.5	4.2	-0.7	-1.0
Diabetes	Yes	2.3	2.7	2.6	0.4	0.3
	No	92.4	92.8	93.2	0.4	0.8
	Unknown	5.3	4.6	4.3	-0.7	-1.0
Atherosclerosis or myocardial infarction	Yes	0.9	1.1	1.1	0.2	0.2
	No	93.8	94.2	94.6	0.5	0.8
	Unknown	5.4	4.7	4.4	-0.7	-1.0
Ischemic stroke	Yes	0.4	0.4	0.4	0.1	0.0
	No	94.3	94.9	95.2	0.7	1.0
	Unknown	5.4	4.7	4.4	-0.7	-1.0
Cancer	Yes	1.0	1.3	1.3	0.3	0.3
	No	93.6	94.1	94.4	0.4	0.7
	Unknown	5.3	4.6	4.3	-0.7	-1.0
Hearing loss	Yes	3.1	3.7	3.6	0.6	0.5
	No	91.6	91.7	92.1	0.1	0.5
	Unknown	5.3	4.6	4.3	-0.7	-1.0
Eczema	Yes	8.0	7.8	8.0	-0.2	0.0
	No	86.7	87.6	87.8	0.9	1.0
	Unknown	5.3	4.6	4.3	-0.7	-1.0
Back disease	Yes	8.7	9.4	9.2	0.7	0.5
	No	86.2	86.2	86.7	0.0	0.5
	Unknown	5.2	4.5	4.2	-0.7	-1.0
Migraine	Yes	5.8	6.0	6.3	0.2	0.5
	No	88.9	89.4	89.5	0.5	0.6
	Unknown	5.3	4.6	4.3	-0.7	-1.0
Another long-term illness	Yes	7.9	8.7	8.7	0.8	0.8
	No	86.9	86.7	87.1	-0.1	0.2
	Unknown	5.2	4.5	4.3	-0.7	-1.0

Notes: The variables in the table (except *Body mass index*) indicates whether a worker was in treatment at questionnaire completion or had been treated during the previous year for a specific health problem. All variables were constructed using information from the WEHD survey.

[a] We obtained the figures for the source population by applying the sample weights associated with observations in the study sample.

Table S7. Descriptive statistics for the explanatory variables included in variable group F (health behaviors)

		Source population[a]	Study sample, N=71 207 (102 379 obs.)	Regression sample, N=48 434 (67 780 obs.)		
		(1)	(2)	(3)	(2)-(1)	(3)-(1)
		% of pop.	% of N	% of N	%pt.	%pt.
Smoking	Yes, on a daily basis	14.0	13.3	12.8	-0.7	-1.2
	Yes, occasionally	5.6	5.0	4.9	-0.6	-0.7
	No, but have been smoking	25.5	28.4	28.9	2.8	3.4
	No, have never smoked	50.7	49.9	50.2	-0.8	-0.5
	Unknown	4.2	3.5	3.2	-0.7	-1.0
Number of cigarettes/cigars/pipe fillings per day	Not smoking	76.3	78.2	79.2	2.0	2.9
	Less than 5	5.5	4.9	4.9	-0.6	-0.6
	5–9	4.1	3.9	3.9	-0.2	-0.2
	10–14	4.3	4.1	4.1	-0.2	-0.2
	15+	5.7	5.3	4.8	-0.3	-0.9
	Unknown	4.2	3.6	3.3	-0.7	-1.0
Alcohol consumption per day, Monday-Thursday (alcohol units)[b]	0	67.8	66.5	67.8	-1.3	0.0
	1	16.7	17.8	17.3	1.0	0.6
	2	6.6	7.3	7.0	0.7	0.4
	3–4	3.0	3.4	3.1	0.3	0.1
	5+	0.8	0.8	0.7	0.0	-0.1
	Unknown	5.1	4.3	4.1	-0.8	-1.1
Alcohol consumption per day, Friday-Sunday (alcohol units)[b]	0	19.3	18.2	18.7	-1.1	-0.7
	1	23.0	23.5	24.1	0.5	1.1
	2	22.0	23.3	23.8	1.3	1.8
	3–4	20.9	22.0	21.7	1.1	0.8
	5+	10.5	9.3	8.4	-1.1	-2.0
	Unknown	4.3	3.6	3.3	-0.7	-1.0
Hours of light exercise per week	4+	27.9	28.2	29.5	0.4	1.6
	2–4	31.1	32.1	32.9	1.1	1.9
	0.1–2	29.2	29.3	28.4	0.0	-0.8
	0	7.2	6.5	5.6	-0.7	-1.6
	Unknown	4.6	3.9	3.6	-0.8	-1.1
Hours of medium exercise per week	4+	13.3	12.4	12.3	-1.0	-1.0
	2–4	28.0	28.5	28.9	0.4	0.9
	0.1–2	36.1	37.5	38.0	1.4	1.9
	0	18.0	17.8	17.2	-0.2	-0.8
	Unknown	4.6	3.9	3.6	-0.7	-1.0
Hours of hard exercise per week	4+	5.1	3.8	3.5	-1.3	-1.7
	2–4	7.4	6.5	6.4	-0.9	-1.0
	0.1–2	12.3	11.4	11.3	-0.9	-1.0
	0	69.9	73.8	74.5	3.9	4.6
	Unknown	5.3	4.6	4.3	-0.7	-1.0

Notes: All variables in the table were constructed using information from the WEHD survey.

[a] We obtained the figures for the source population by applying the sample weights associated with observations in the study sample.

[b] One unit of alcohol equals 15 ml of pure alcohol.

Appendix 3. The algorithm for estimating economic effects of hypothetical improvements of the psychosocial work environment

Our algorithm for estimating cost changes from hypothetical improvements of the psychosocial work environment (PSWE) used a number of steps, which were slightly different when estimating changes in costs of sickness absence (SA) and costs of health care use.

When estimating changes in SA costs, we used in the first step information from the regression sample to estimate a hurdle model (2). This two-part model used a logit model to describe the probability of experiencing SA within the measurement period and a zero-truncated negative binomial model to describe the number of SA hours if SA occurred.

In the second step, we used the parameter estimates obtained in step 1 and study sample information to calculate the expected hours of SA over a one-year period for each study sample observation in the different PSWE situations we compared. For example, for the hypothetical situation where all workers experience the most desirable PSWE with respect to all PSWE factors, we calculated the expected hours of SA by setting the values of all 17 PSWE variables equal to “Most desirable”, length of the period over which we measure hours of SA to one year and the remaining covariates to their observed values. If workers have no superior, leadership quality is not changeable. Therefore, when the observed value of the variable “Quality of leadership” equaled “No superior”, we retained this value of “Quality of leadership” in all hypothetical PSWE situations.

In the third step, we calculated situation-specific costs of SA for each study sample observation as a product between the expected hours of SA from step 2 and an observation-specific hourly wage rate. We estimated the hourly wage rate as the ratio between wage earnings and hours of work in the main job around the date of questionnaire completion. We restricted the hourly wage rates (in 2023 euros) to be in the range €7 to €268 (50 to 2000 Danish Kroner).

In the fourth step, we calculated sample-weighted situation-specific averages of the SA costs from step 3.

In the fifth step, we used the averages from step 4 to compute the difference between expected average SA costs per worker in the most and least desirable PSWE situation and in the most desirable and observed PSWE situation, respectively.

In the sixth and final step, using the delta method (3), we calculated standard errors and 95% confidence intervals for the cost differences computed in step 5. For doing this, we used the cluster-robust variance-covariance matrix of the parameter estimates obtained in step 1 (with clustering at the worker level) (4).

When we estimated changes in health care use costs from hypothetical improvements of the PSWE, costs of health care use replaced hours of SA as the dependent variable in steps 1 to 2 of the algorithm above, step 3 was omitted, and steps 4 to 6 used costs of health care use as cost measure instead of costs of SA. We made separate estimates for our four health care cost measures: costs of prescription drug use, costs of primary health care use, costs of hospital treatment and total health care costs.

Finally, we calculated estimates of the total cost changes as the sum of the estimated changes in costs of SA and total health care costs.

Appendix 4. Estimates stratified by sex and cost types

Figure S1. Gender-specific estimates with 95% confidence intervals for annual economic gains per worker from two hypothetical improvements of the general psychosocial work environment. Parametric g-formula analyses with adjustment for sociodemographic characteristics, job characteristics, physical work environment characteristics, health status and health behaviors.

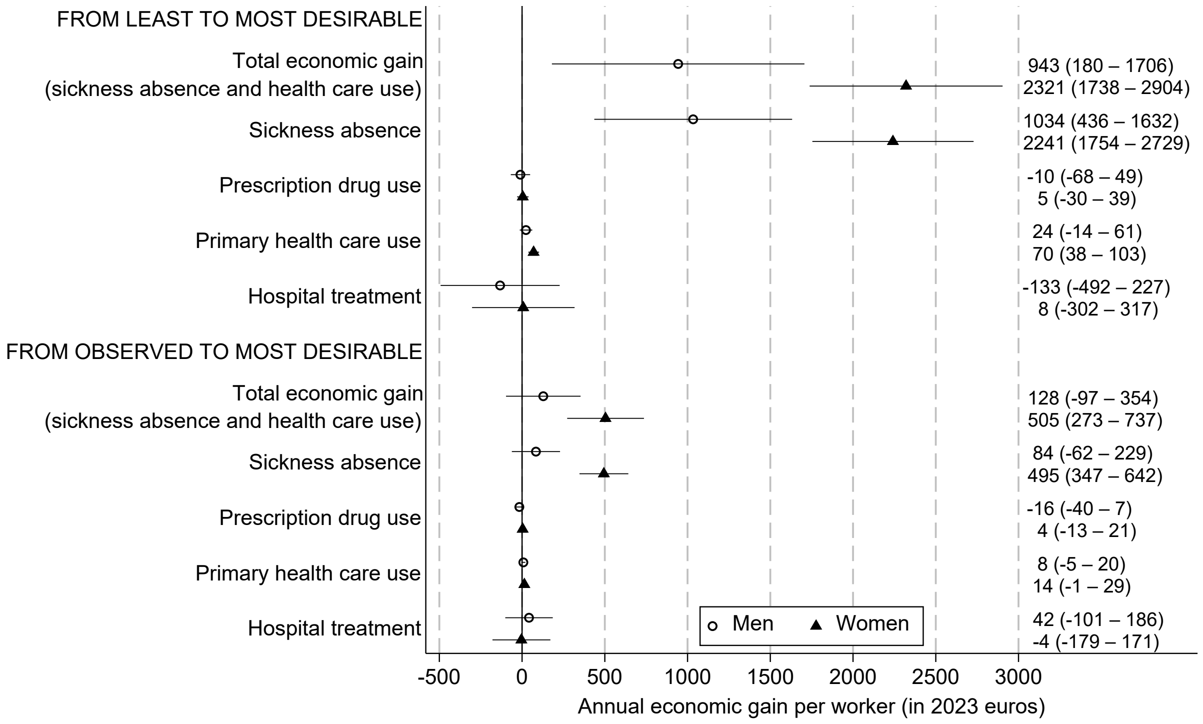

Figure S2. Estimates with 95% confidence intervals for reductions in annual costs of sickness absence per worker from hypothetical improvements (least to most desirable) of specific psychosocial work environment factors. Parametric g-formula analyses with adjustment for sociodemographic characteristics, job characteristics, physical work environment characteristics, health status and health behaviors.

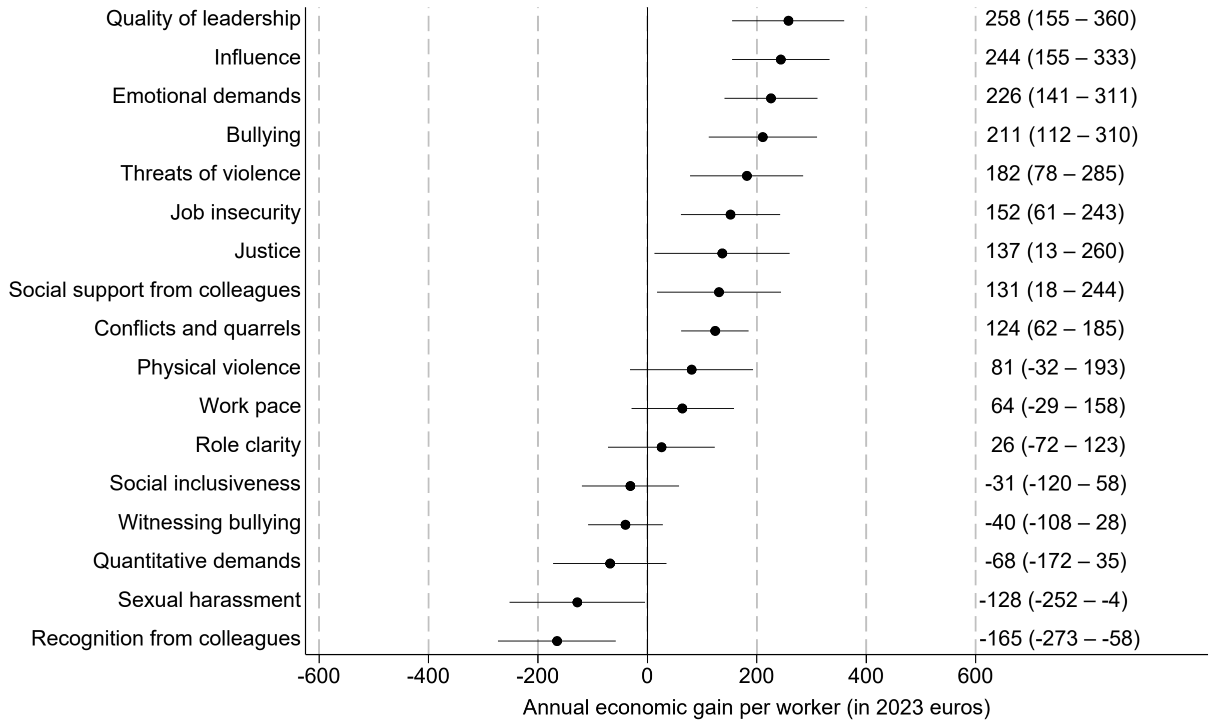

Figure S3. Estimates with 95% confidence intervals for reductions in annual costs of sickness absence per worker from hypothetical improvements (observed to most desirable) of specific psychosocial work environment factors. Parametric g-formula analyses with adjustment for sociodemographic characteristics, job characteristics, physical work environment characteristics, health status and health behaviors.

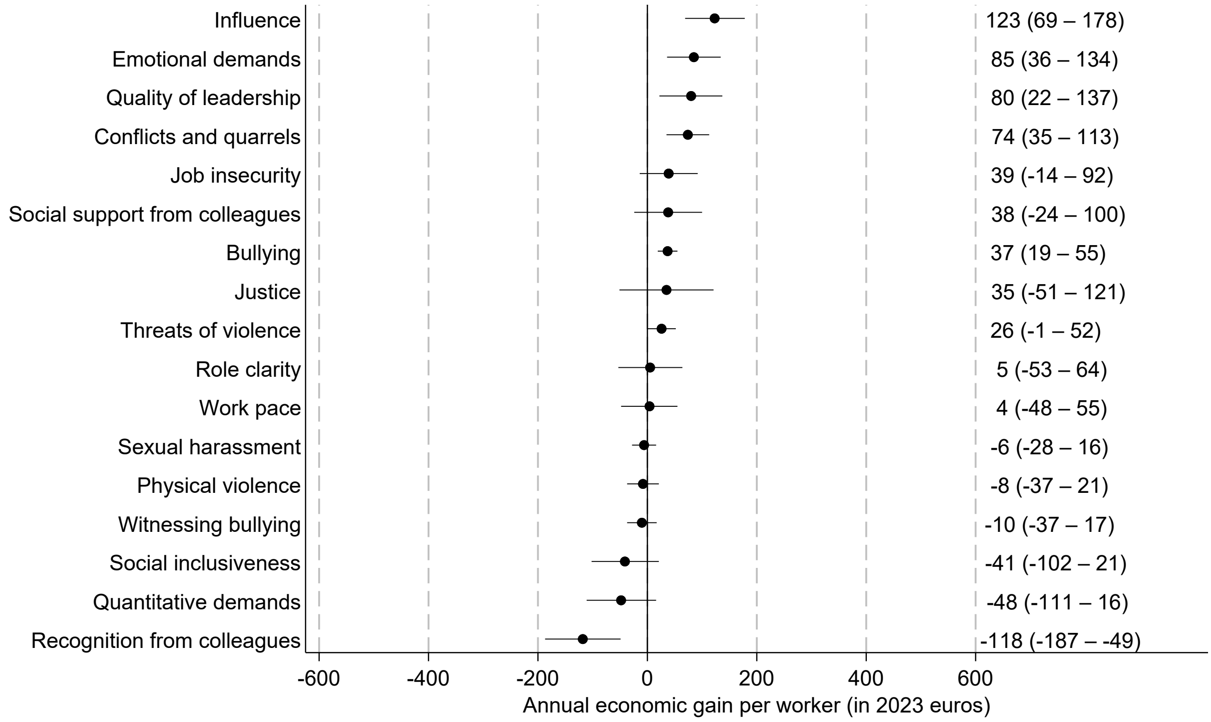

Figure S4. Estimates with 95% confidence intervals for reductions in annual costs of health care use per worker from hypothetical improvements (least to most desirable) of specific psychosocial work environment factors. Parametric g-formula analyses with adjustment for sociodemographic characteristics, job characteristics, physical work environment characteristics, health status and health behaviors.

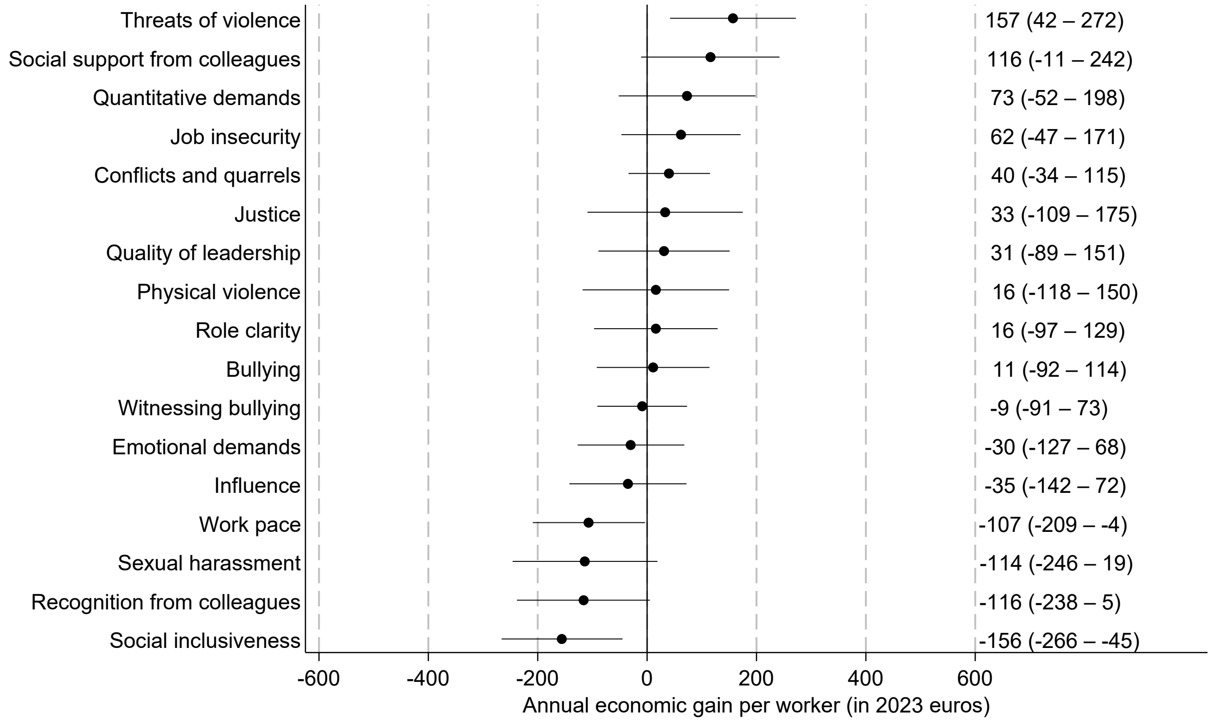

Figure S5. Estimates with 95% confidence intervals for reductions in annual costs of health care use per worker from hypothetical improvements (observed to most desirable) of specific psychosocial work environment factors. Parametric g-formula analyses with adjustment for sociodemographic characteristics, job characteristics, physical work environment characteristics, health status and health behaviors.

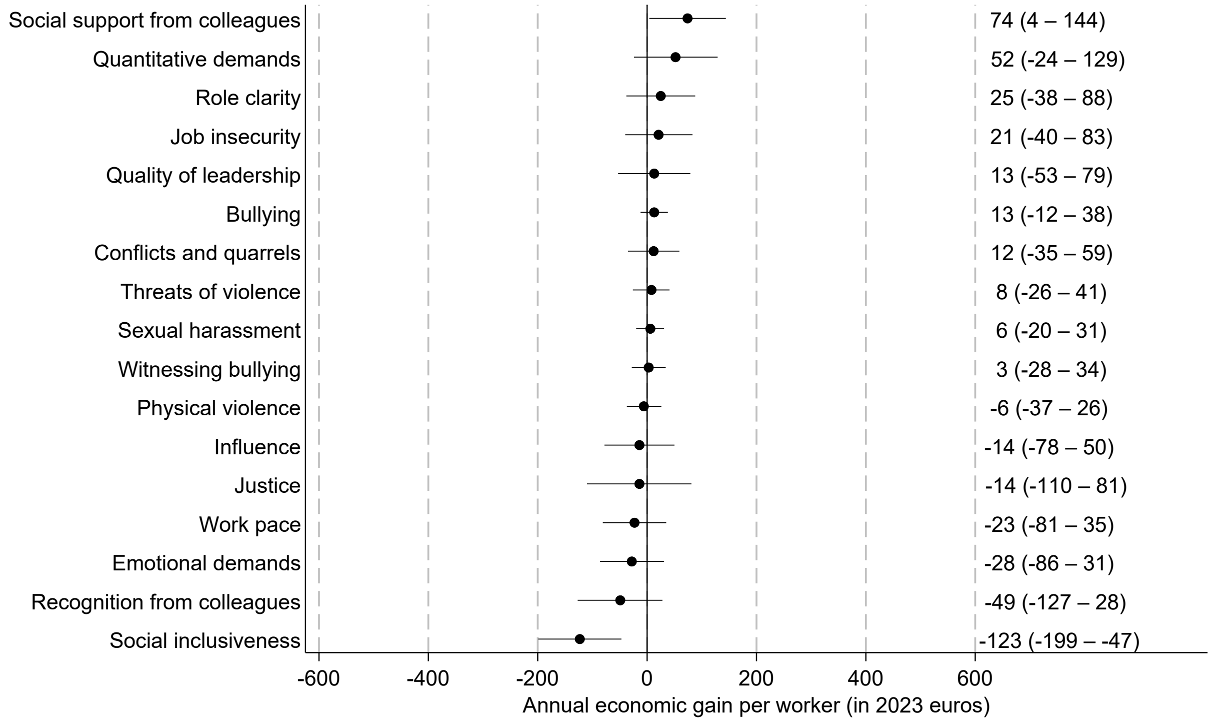

Figure S6. Men. Estimates with 95% confidence intervals for annual economic gains per worker from hypothetical improvements (least to most desirable) of specific psychosocial work environment factors. Parametric g-formula analyses with adjustment for sociodemographic characteristics, job characteristics, physical work environment characteristics, health status and health behaviors.

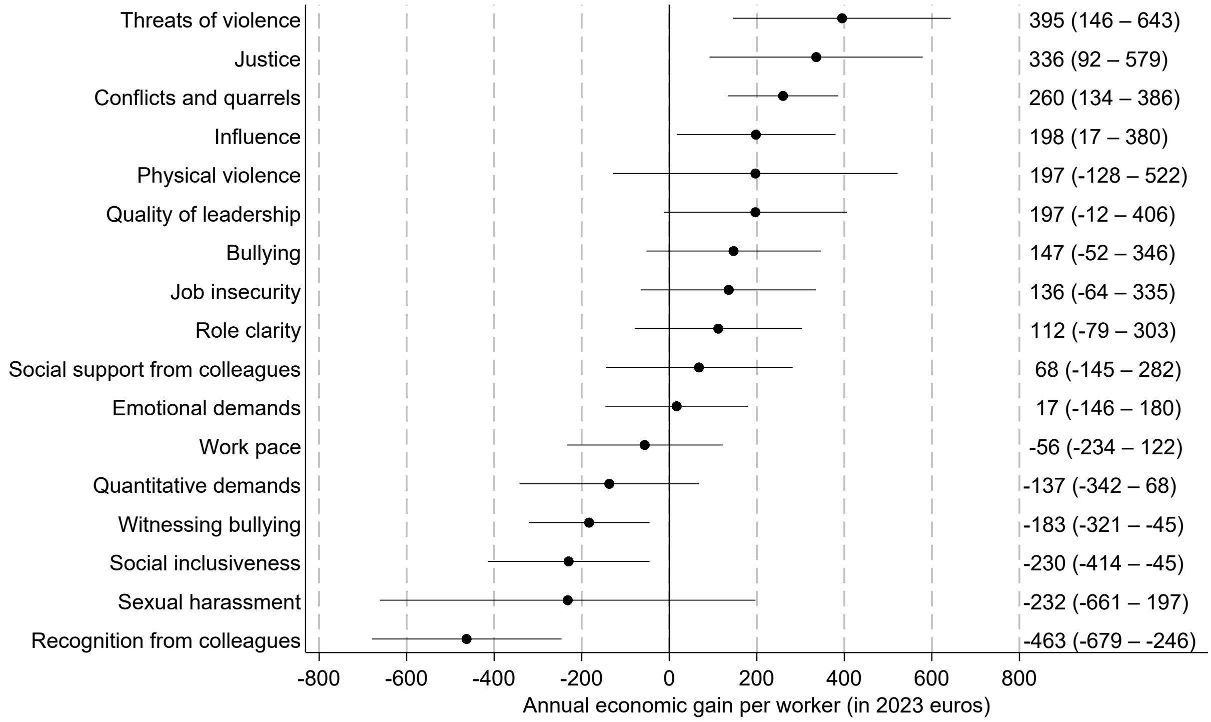

Figure S7. Women. Estimates with 95% confidence intervals for annual economic gains per worker from hypothetical improvements (least to most desirable) of specific psychosocial work environment factors. Parametric g-formula analyses with adjustment for sociodemographic characteristics, job characteristics, physical work environment characteristics, health status and health behaviors.

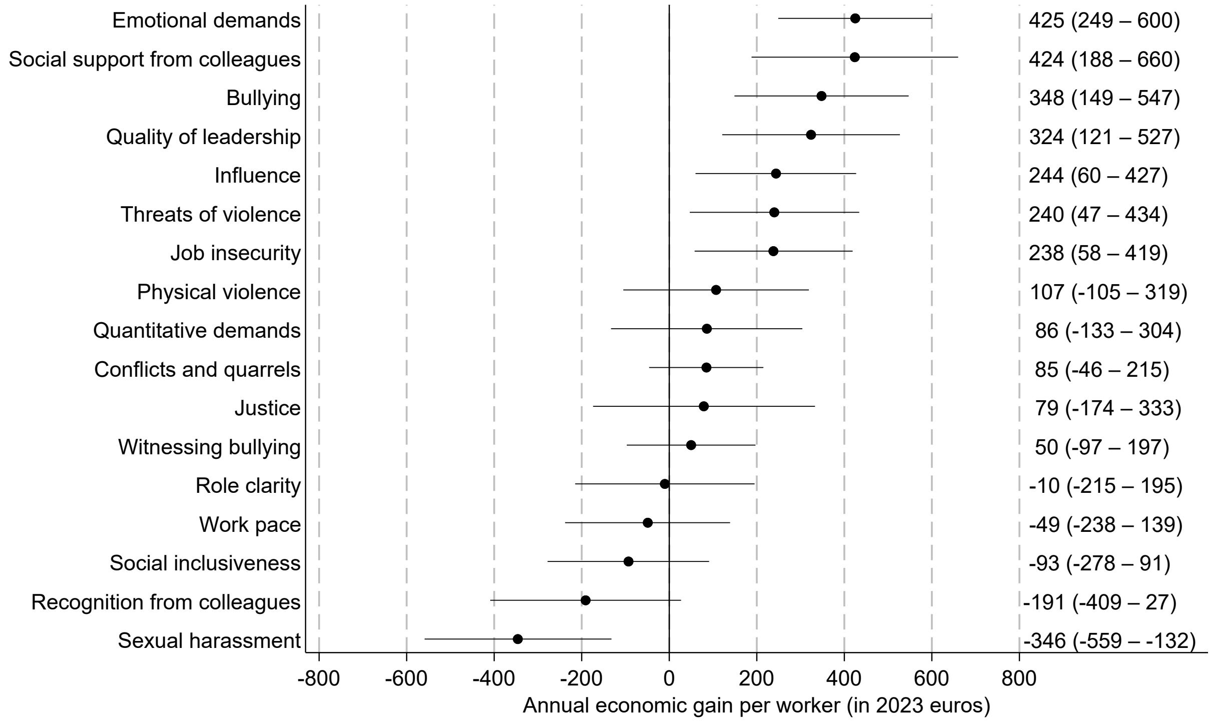

Figure S8. Men. Estimates with 95% confidence intervals for annual economic gains per worker from hypothetical improvements (observed to most desirable) of specific psychosocial work environment factors. Parametric g-formula analyses with adjustment for sociodemographic characteristics, job characteristics, physical work environment characteristics, health status and health behaviors.

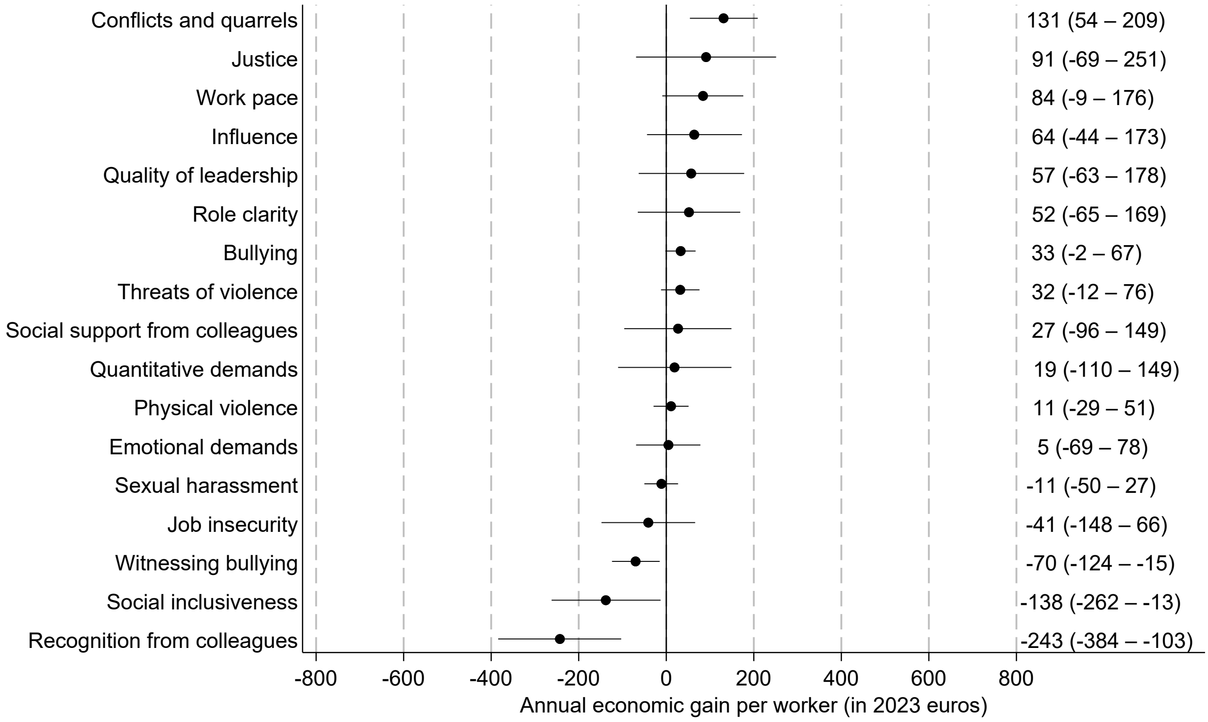

Figure S9. Women. Estimates with 95% confidence intervals for annual economic gains per worker from hypothetical improvements (observed to most desirable) of specific psychosocial work environment factors. Parametric g-formula analyses with adjustment for sociodemographic characteristics, job characteristics, physical work environment characteristics, health status and health behaviors.

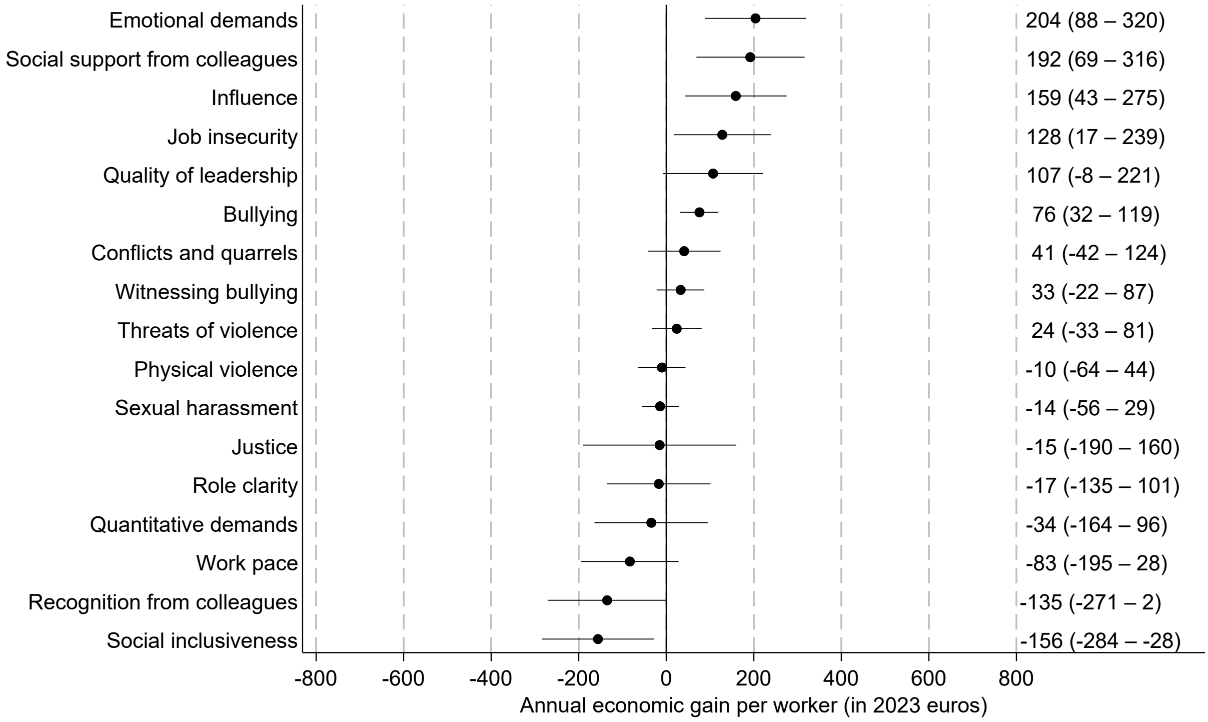

Figure S10. Men. Estimates with 95% confidence intervals for reductions in annual costs of sickness absence per worker from hypothetical improvements (least to most desirable) of specific psychosocial work environment factors. Parametric g-formula analyses with adjustment for sociodemographic characteristics, job characteristics, physical work environment characteristics, health status and health behaviors.

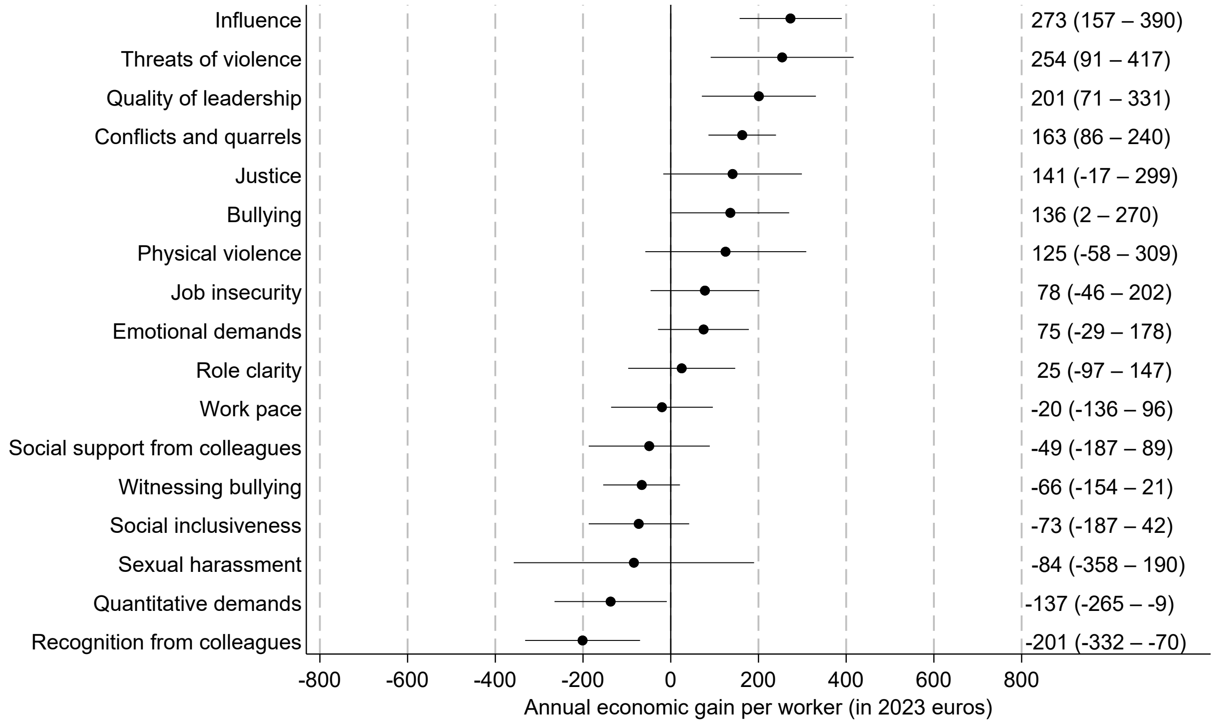

Figure S11. Women. Estimates with 95% confidence intervals for reductions in annual costs of sickness absence per worker from hypothetical improvements (least to most desirable) of specific psychosocial work environment factors. Parametric g-formula analyses with adjustment for sociodemographic characteristics, job characteristics, physical work environment characteristics, health status and health behaviors.

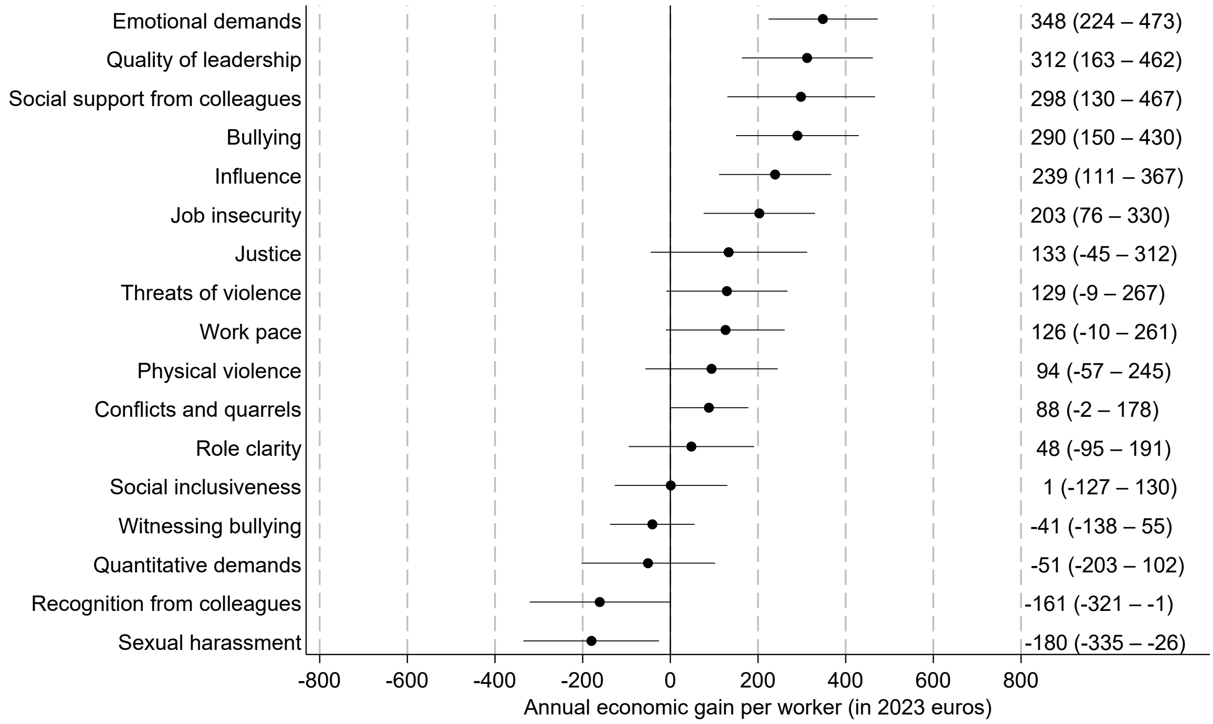

Figure S12. Men. Estimates with 95% confidence intervals for reductions in annual costs of sickness absence per worker from hypothetical improvements (observed to most desirable) of specific psychosocial work environment factors. Parametric g-formula analyses with adjustment for sociodemographic characteristics, job characteristics, physical work environment characteristics, health status and health behaviors.

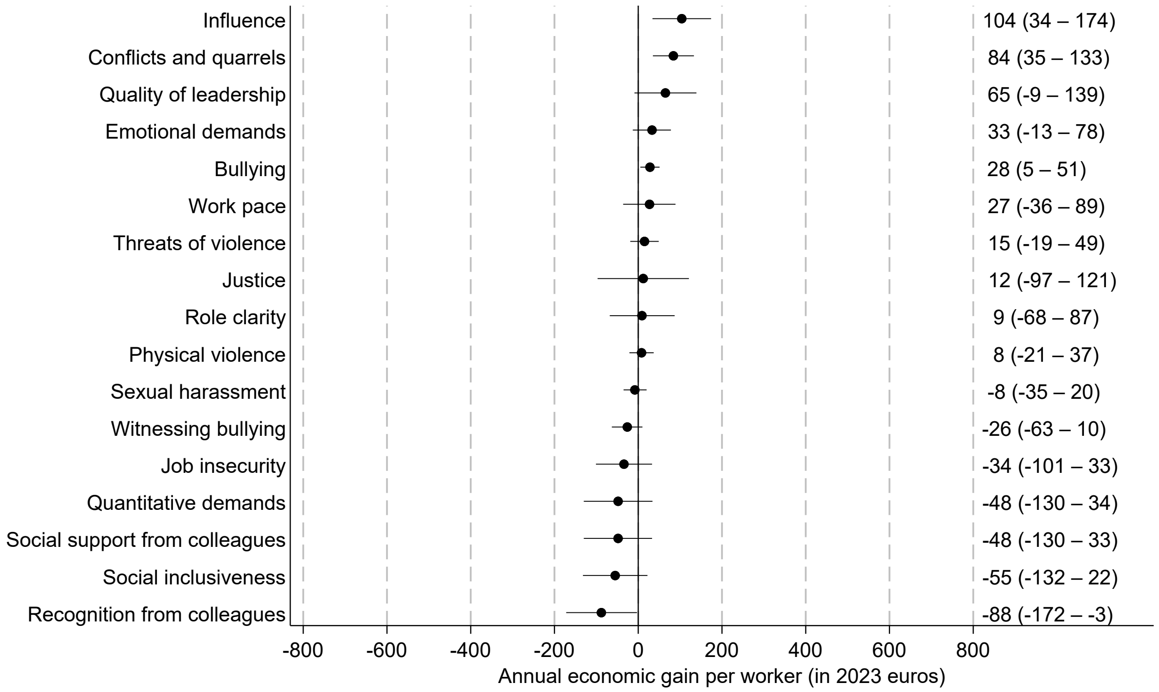

Figure S13. Women. Estimates with 95% confidence intervals for reductions in annual costs of sickness absence per worker from hypothetical improvements (observed to most desirable) of specific psychosocial work environment factors. Parametric g-formula analyses with adjustment for sociodemographic characteristics, job characteristics, physical work environment characteristics, health status and health behaviors.

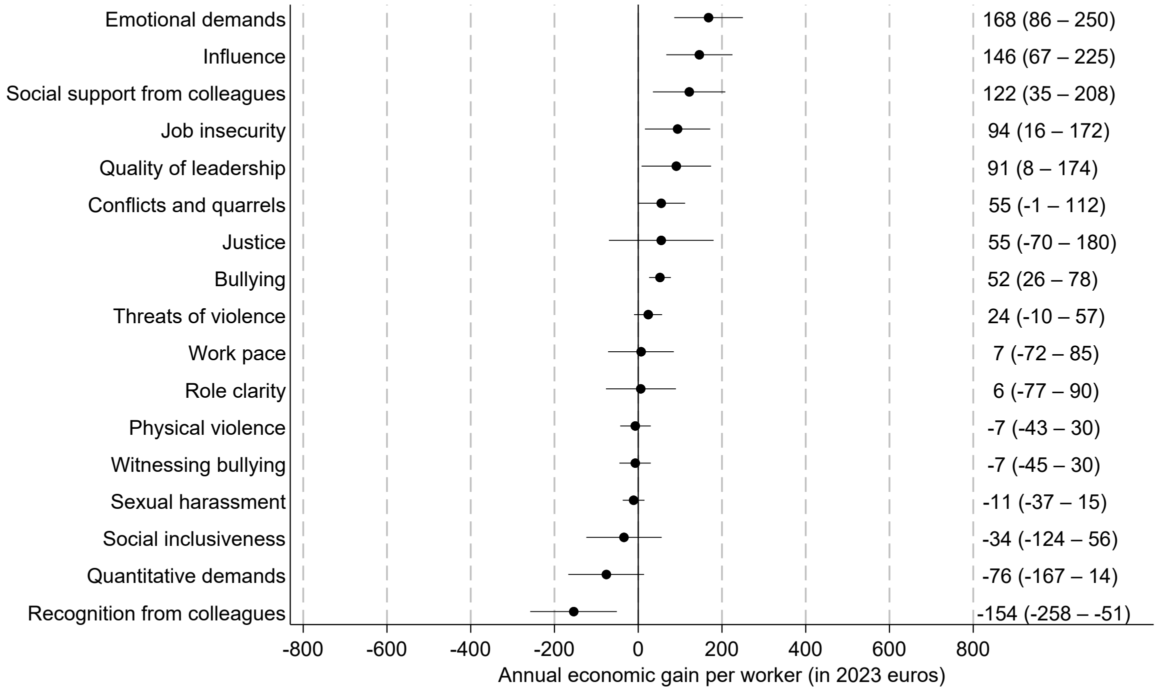

Figure S14. Men. Estimates with 95% confidence intervals for reductions in annual costs of health care use per worker from hypothetical improvements (least to most desirable) of specific psychosocial work environment factors. Parametric g-formula analyses with adjustment for sociodemographic characteristics, job characteristics, physical work environment characteristics, health status and health behaviors.

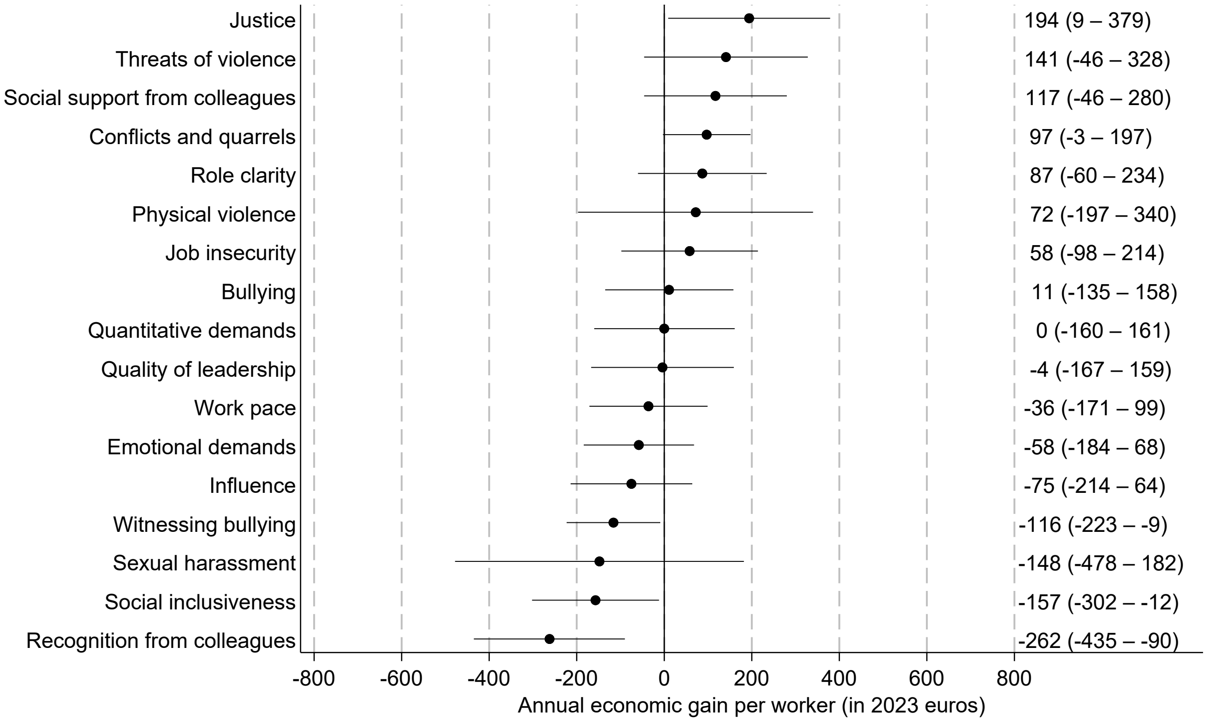

Figure S15. Women. Estimates with 95% confidence intervals for reductions in annual costs of health care use per worker from hypothetical improvements (least to most desirable) of specific psychosocial work environment factors. Parametric g-formula analyses with adjustment for sociodemographic characteristics, job characteristics, physical work environment characteristics, health status and health behaviors.

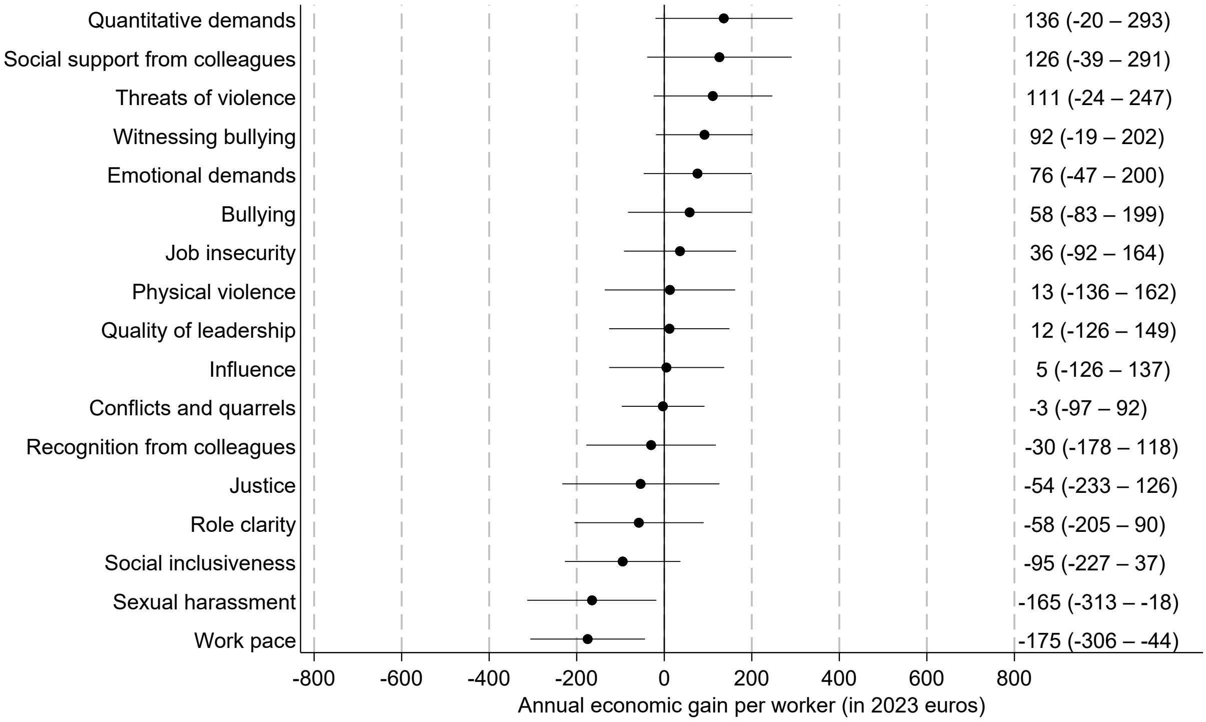

Figure S16. Men. Estimates with 95% confidence intervals for reductions in annual costs of health care use per worker from hypothetical improvements (observed to most desirable) of specific psychosocial work environment factors. Parametric g-formula analyses with adjustment for sociodemographic characteristics, job characteristics, physical work environment characteristics, health status and health behaviors.

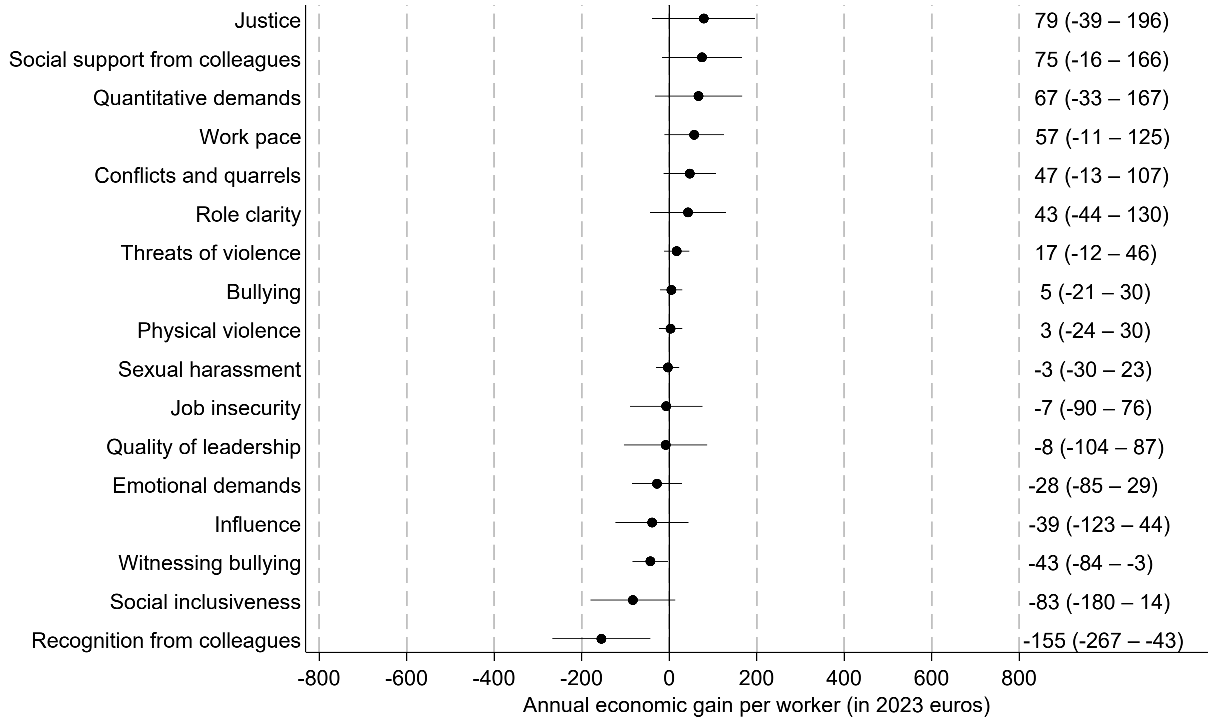

Figure S17. Women. Estimates with 95% confidence intervals for reductions in annual costs of health care use per worker from hypothetical improvements (observed to most desirable) of specific psychosocial work environment factors. Parametric g-formula analyses with adjustment for sociodemographic characteristics, job characteristics, physical work environment characteristics, health status and health behaviors.

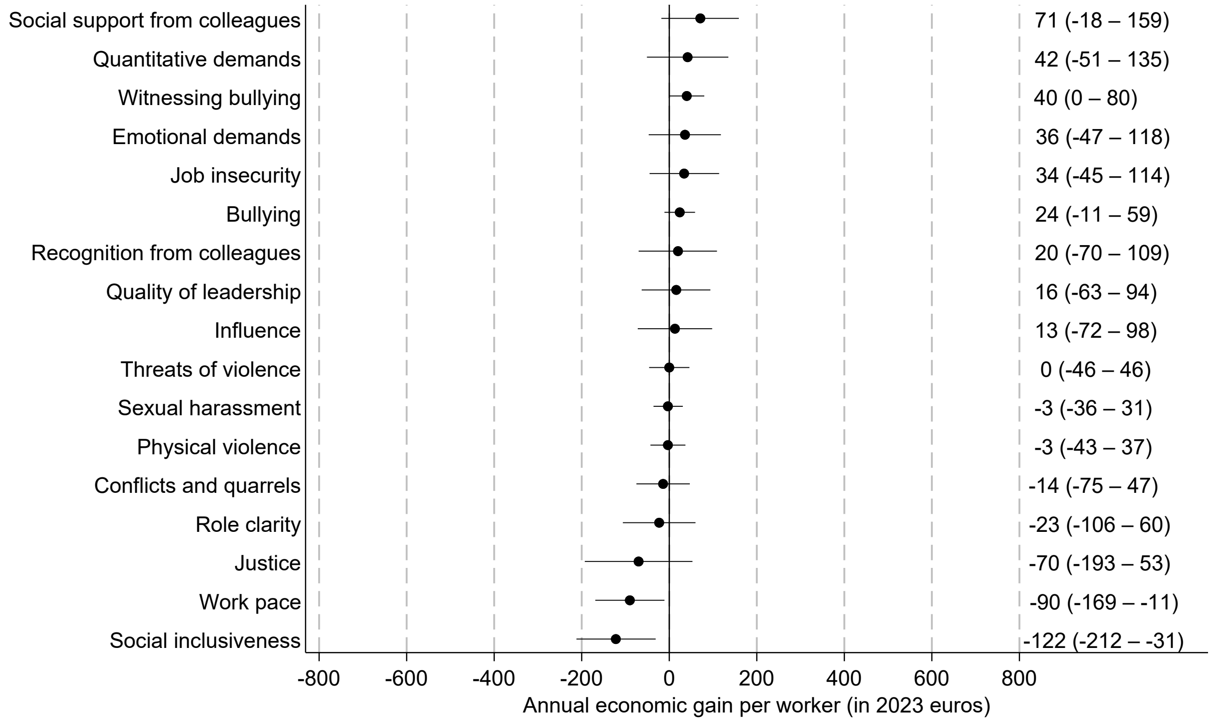

Appendix 5. Estimates for different sectors and industries

Table S8. Estimates with standard errors and 95% confidence intervals for annual economic gains per worker from two hypothetical improvements of the general psychosocial work environment. Parametric g-formula analyses with adjustment for sociodemographic characteristics, job characteristics, physical work environment characteristics, health status and health behaviors.

	FROM LEAST TO MOST DESIRABLE				FROM OBSERVED TO MOST DESIRABLE			
	Estimate	Standard error	95% CI, lower bound	95% CI, upper bound	Estimate	Standard error	95% CI, lower bound	95% CI, upper bound
ALL WORKERS (67 780 obs.)								
Total economic gain	1685	230	1234	2135	305	87	134	476
Sickness absence	1705	185	1343	2067	301	53	196	406
Health care use	-21	137	-289	247	4	69	-131	139
PUBLIC SECTOR (43 537 obs.)[a]								
Total economic gain	2278	300	1690	2866	521	120	287	756
Sickness absence	2144	242	1670	2619	475	75	328	623
Health care use	133	177	-213	480	46	93	-136	228
PRIVATE COMPANIES (23 482 obs.)								
Total economic gain	254	416	-562	1070	61	129	-192	314
Sickness absence	852	364	138	1565	148	78	-5	300
Health care use	-598	202	-994	-202	-87	103	-289	116
MANUFACTURING, MINING AND QUARRYING[b] (7968 obs.)								
Total economic gain	460	1084	-1665	2585	-89	212	-504	326
Sickness absence	1014	1006	-959	2986	6	145	-279	291
Health care use	-554	403	-1344	236	-95	154	-397	207
PUBLIC ADMINISTRATION, DEFENCE AND POLICE[c] (6982 obs.)								
Total economic gain	1262	682	-74	2598	637	220	207	1068
Sickness absence	1586	572	464	2707	667	121	429	905
Health care use	-324	370	-1050	402	-30	183	-389	329
EDUCATION[c] (10 242 obs.)								
Total economic gain[d]	-	-	-	-	-	-	-	-
Sickness absence	2905	680	1573	4238	658	144	376	939
Health care use[d]	-	-	-	-	-	-	-	-
HUMAN HEALTH ACTIVITIES[c] (7368 obs.)								
Total economic gain	2198	645	933	3463	81	281	-470	632
Sickness absence	2377	558	1284	3470	272	195	-111	654
Health care use	-179	325	-816	458	-191	202	-588	206
SOCIAL INSTITUTIONS[c] (14 187 obs.)								
Total economic gain	2879	452	1992	3766	847	179	495	1198
Sickness absence	2373	363	1661	3085	653	123	412	894
Health care use	506	270	-23	1035	193	131	-63	449
WORKERS NOT EMPLOYED IN SOCIAL INSTITUTIONS (53 593 OBS.)								
Total economic gain	1639	287	1077	2202	197	96	8	385
Sickness absence	1731	237	1266	2196	227	60	109	345
Health care use	-92	162	-409	225	-30	75	-178	117

Notes: Numbers of observations in the table are numbers of observations used to estimate the regression models.

[a] Includes workers employed in the following sector categories: "State", "Regions", Municipalities" and "Public companies".

[b] Workplaces in this industry are predominantly private companies.

[c] Workplaces in this industry are predominantly public sector institutions or companies.

[d] We were not able to estimate the hurdle model for health care use costs.

Figure S18. Public sector. Estimates with 95% confidence intervals for annual economic gains per worker from hypothetical improvements (least to most desirable) of specific psychosocial work environment factors. Parametric g-formula analyses with adjustment for sociodemographic characteristics, job characteristics, physical work environment characteristics, health status and health behaviors.

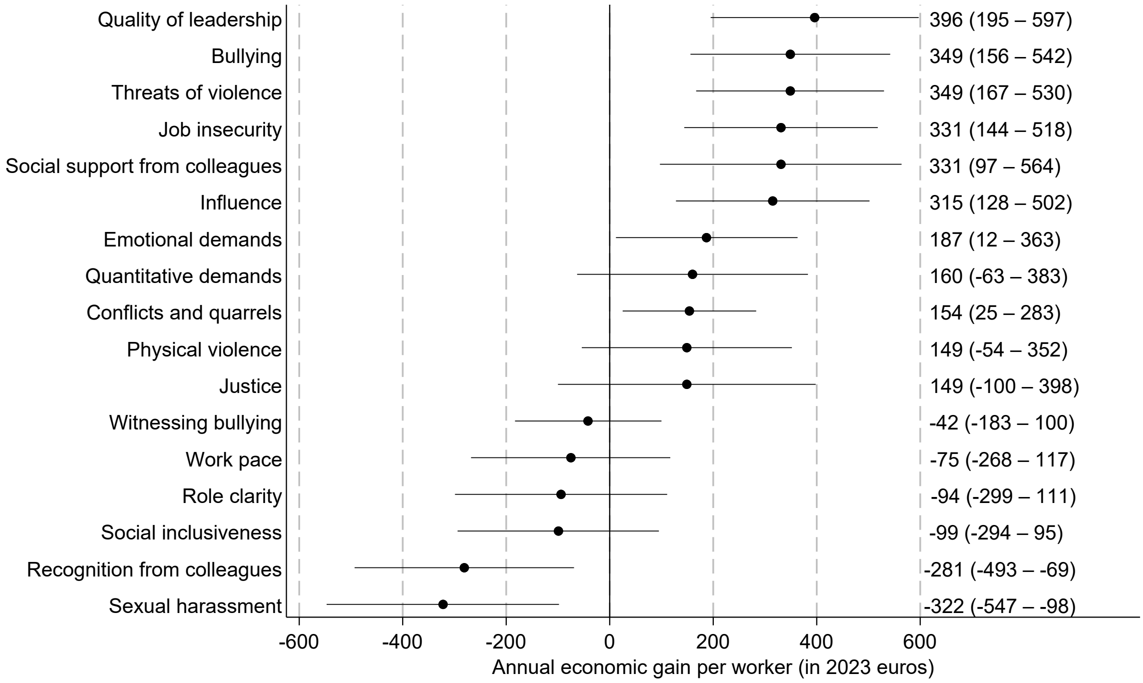

Figure S19. Public sector. Estimates with 95% confidence intervals for annual economic gains per worker from hypothetical improvements (observed to most desirable) of specific psychosocial work environment factors. Parametric g-formula analyses with adjustment for sociodemographic characteristics, job characteristics, physical work environment characteristics, health status and health behaviors.

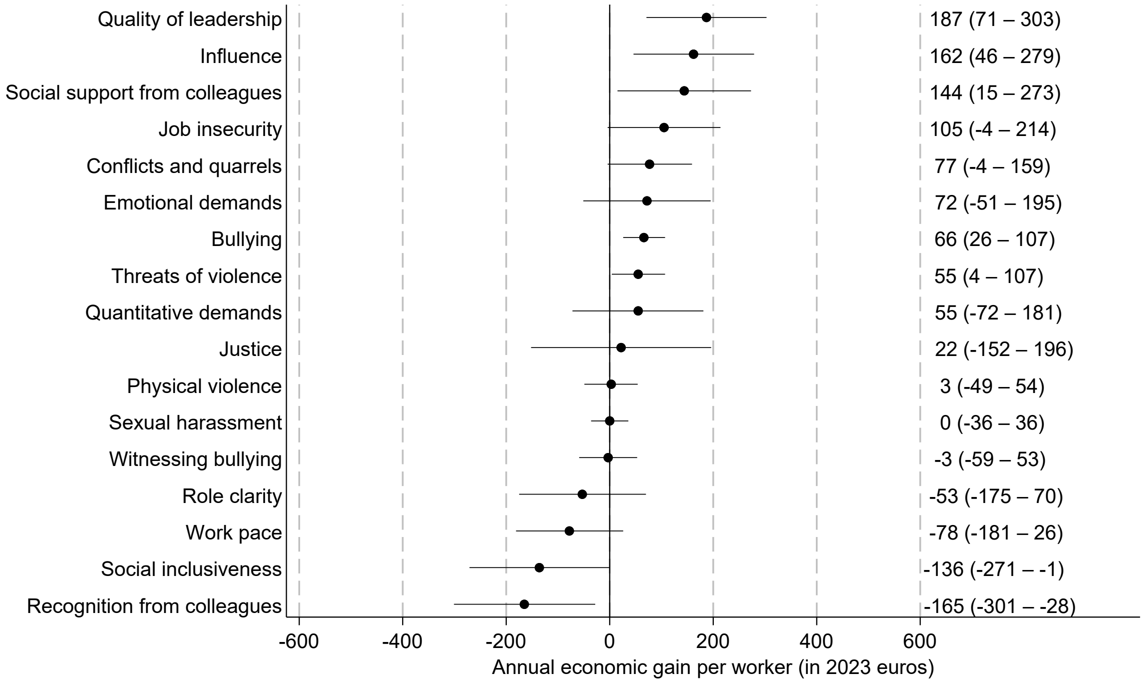

Figure S20. Public sector. Estimates with 95% confidence intervals for reductions in annual costs of sickness absence per worker from hypothetical improvements (least to most desirable) of specific psychosocial work environment factors. Parametric g-formula analyses with adjustment for sociodemographic characteristics, job characteristics, physical work environment characteristics, health status and health behaviors.

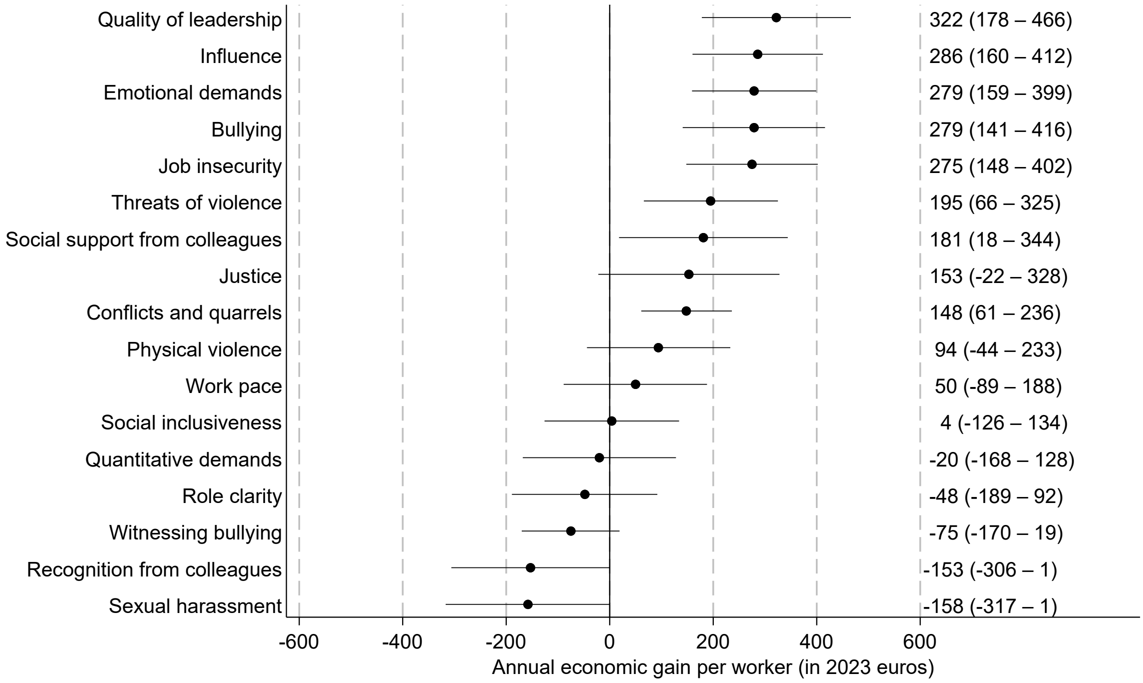

Figure S21. Public sector. Estimates with 95% confidence intervals for reductions in annual costs of sickness absence per worker from hypothetical improvements (observed to most desirable) of specific psychosocial work environment factors. Parametric g-formula analyses with adjustment for sociodemographic characteristics, job characteristics, physical work environment characteristics, health status and health behaviors.

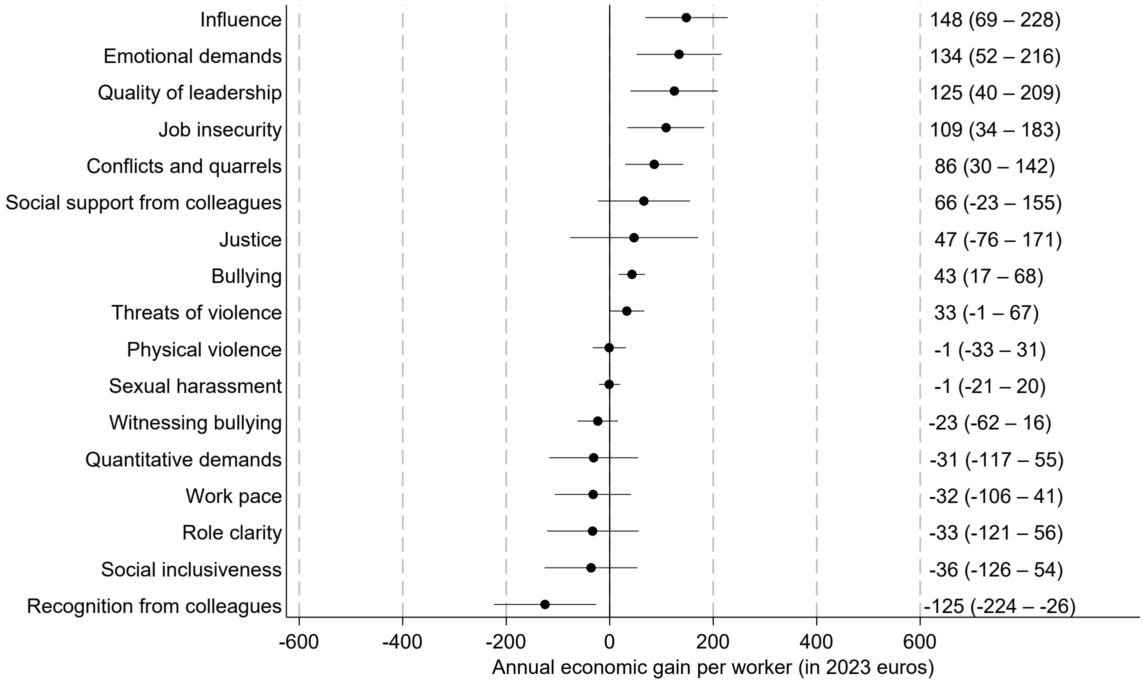

Figure S22. Public sector. Estimates with 95% confidence intervals for reductions in annual costs of health care use per worker from hypothetical improvements (least to most desirable) of specific psychosocial work environment factors. Parametric g-formula analyses with adjustment for sociodemographic characteristics, job characteristics, physical work environment characteristics, health status and health behaviors.

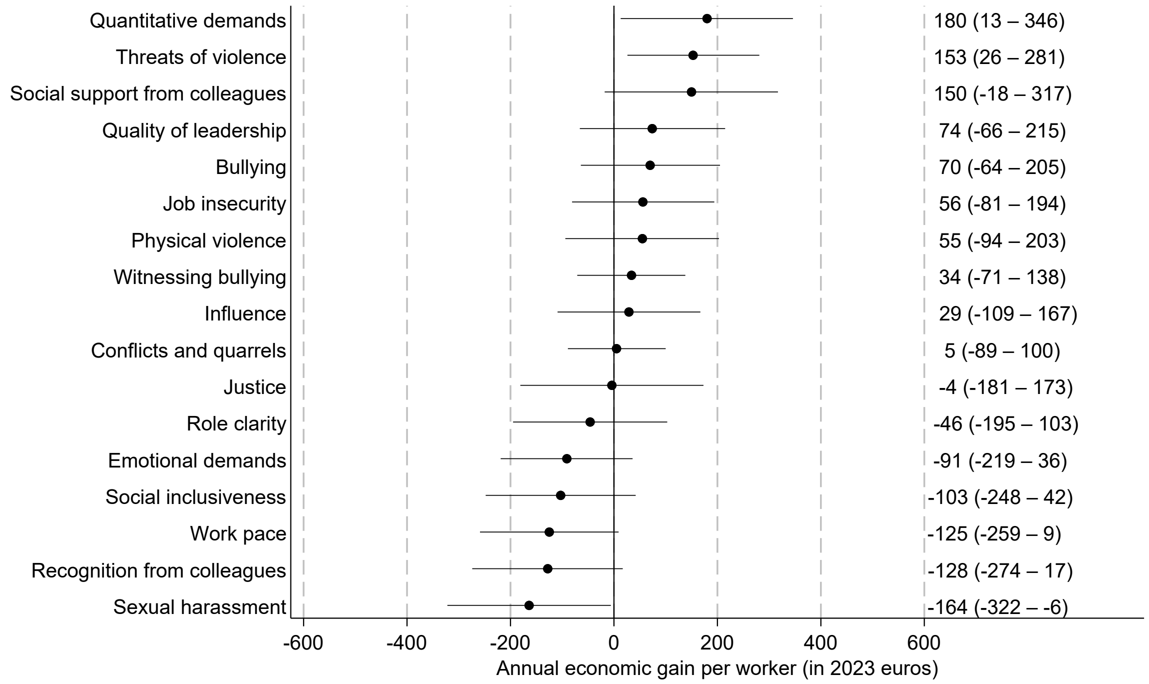

Figure S23. Public sector. Estimates with 95% confidence intervals for reductions in annual costs of health care use per worker from hypothetical improvements (observed to most desirable) of specific psychosocial work environment factors. Parametric g-formula analyses with adjustment for sociodemographic characteristics, job characteristics, physical work environment characteristics, health status and health behaviors.

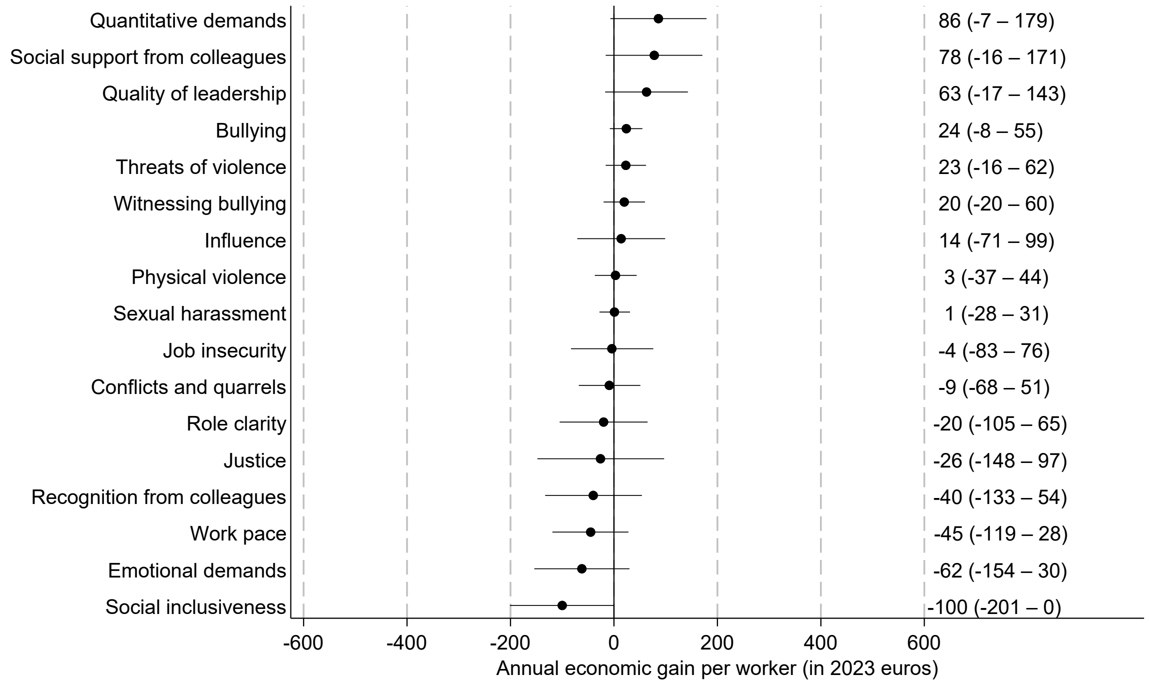

Figure S24. Private companies. Estimates with 95% confidence intervals for annual economic gains per worker from hypothetical improvements (least to most desirable) of specific psychosocial work environment factors. Parametric g-formula analyses with adjustment for sociodemographic characteristics, job characteristics, physical work environment characteristics, health status and health behaviors.

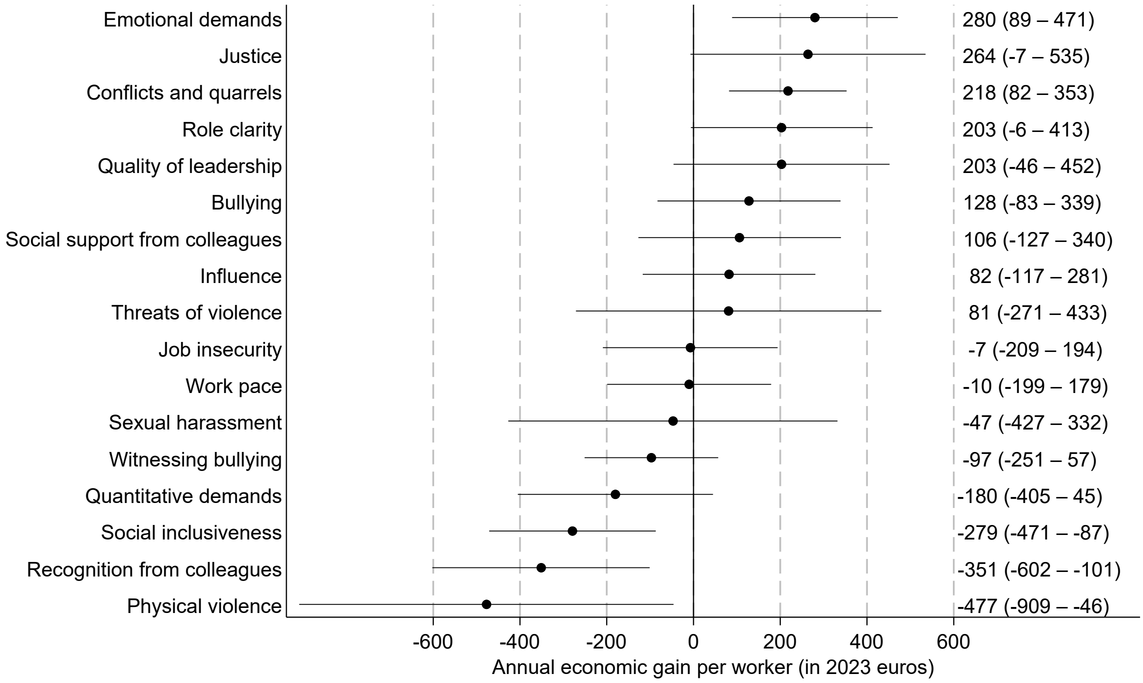

Figure S25. Private companies. Estimates with 95% confidence intervals for annual economic gains per worker from hypothetical improvements (observed to most desirable) of specific psychosocial work environment factors. Parametric g-formula analyses with adjustment for sociodemographic characteristics, job characteristics, physical work environment characteristics, health status and health behaviors.

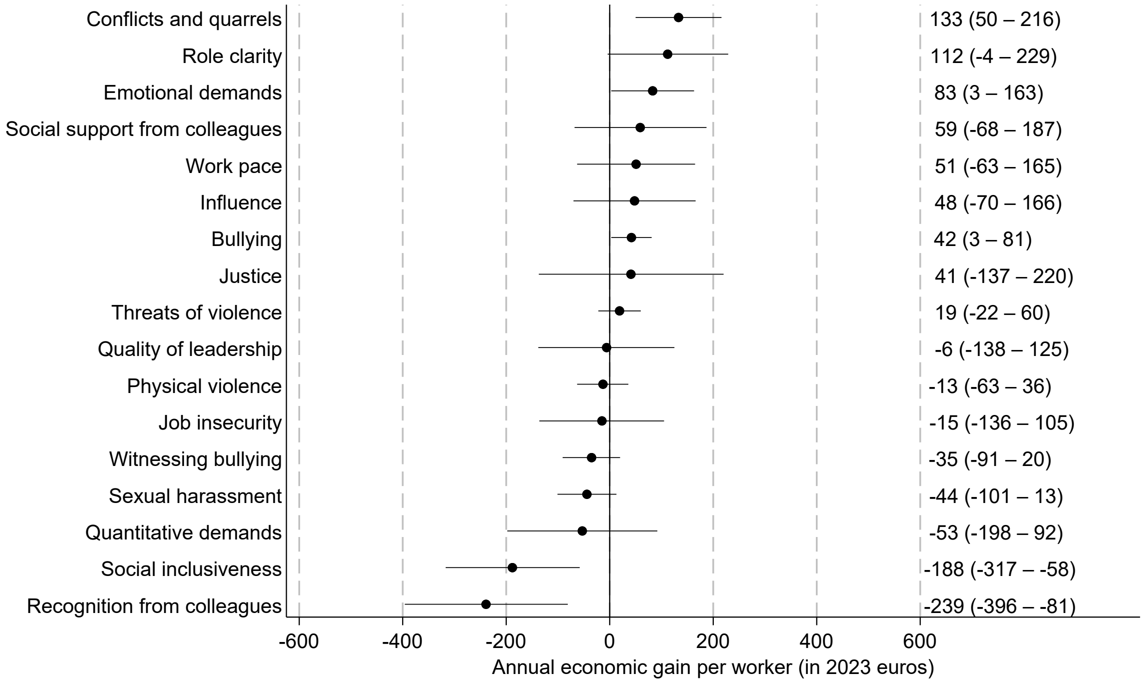

Figure S26. Private companies. Estimates with 95% confidence intervals for reductions in annual costs of sickness absence per worker from hypothetical improvements (least to most desirable) of specific psychosocial work environment factors. Parametric g-formula analyses with adjustment for sociodemographic characteristics, job characteristics, physical work environment characteristics, health status and health behaviors.

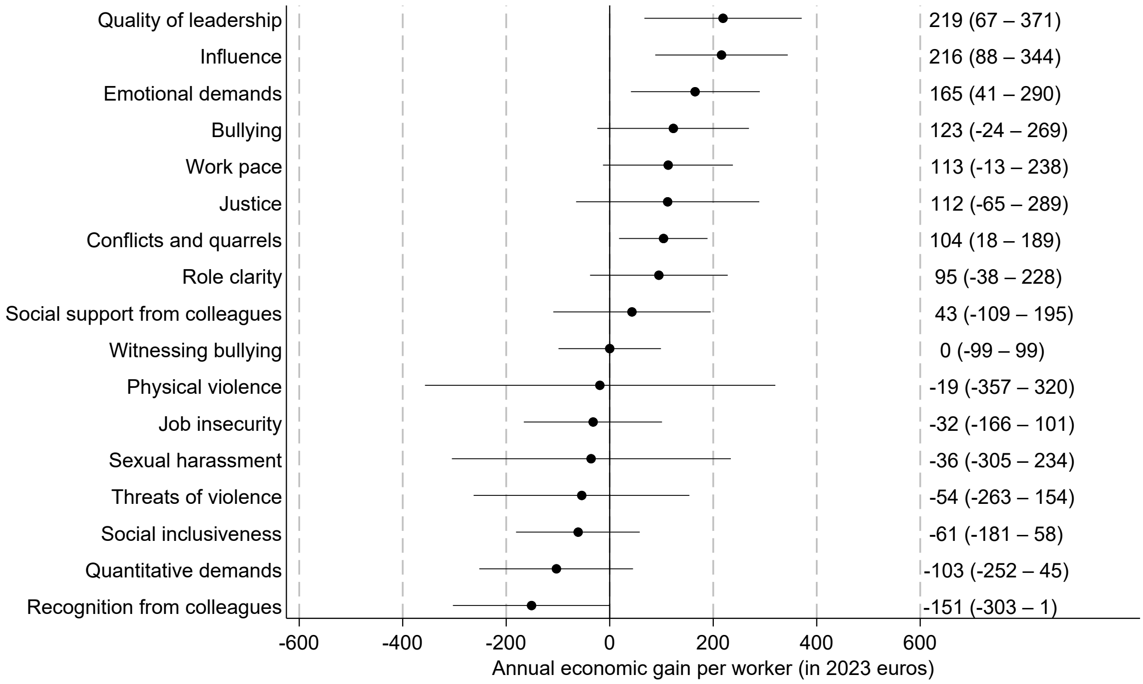

Figure S27. Private companies. Estimates with 95% confidence intervals for reductions in annual costs of sickness absence per worker from hypothetical improvements (observed to most desirable) of specific psychosocial work environment factors. Parametric g-formula analyses with adjustment for sociodemographic characteristics, job characteristics, physical work environment characteristics, health status and health behaviors.

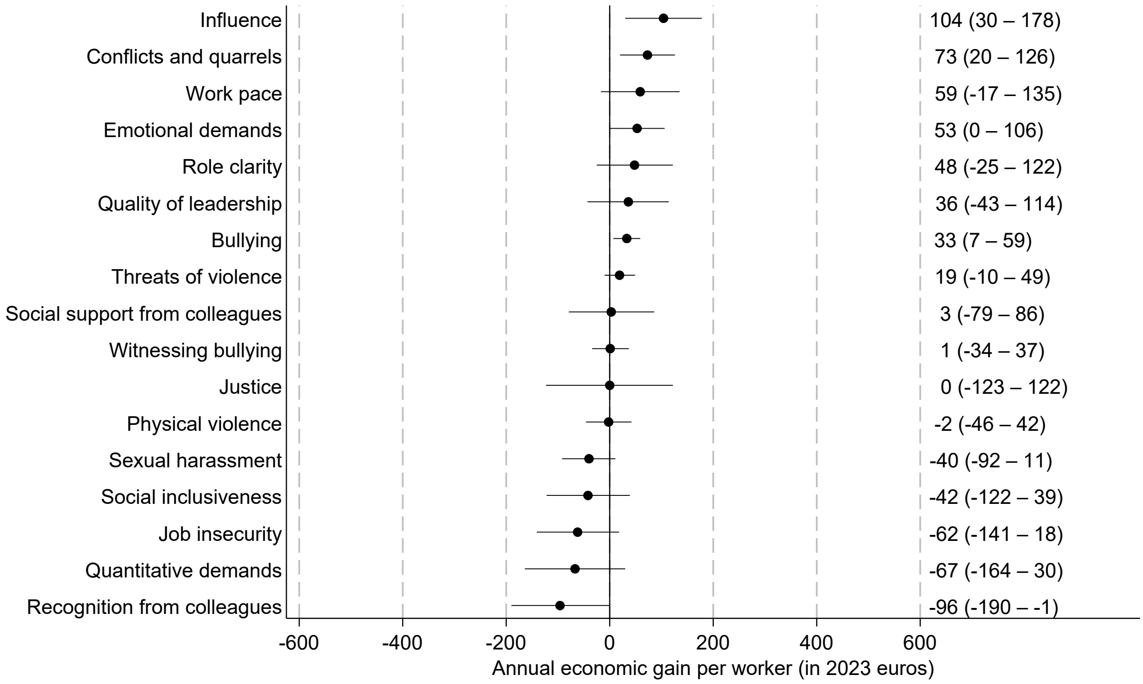

Figure S28. Private companies. Estimates with 95% confidence intervals for reductions in annual costs of health care use per worker from hypothetical improvements (least to most desirable) of specific psychosocial work environment factors. Parametric g-formula analyses with adjustment for sociodemographic characteristics, job characteristics, physical work environment characteristics, health status and health behaviors.

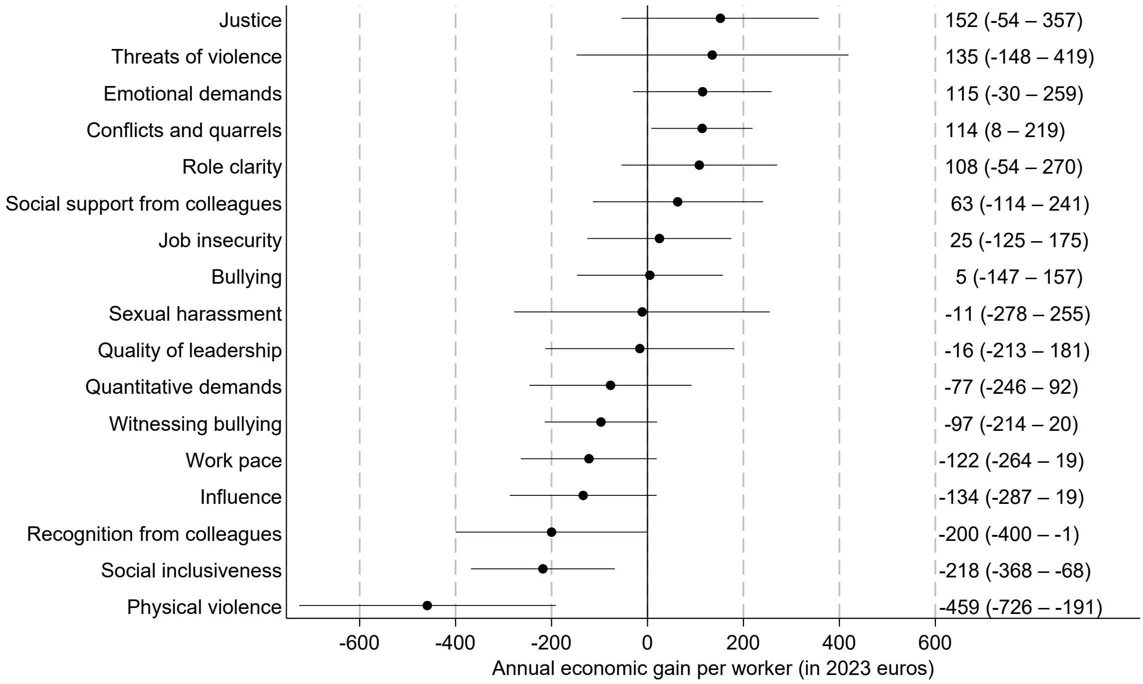

Figure S29. Private companies. Estimates with 95% confidence intervals for reductions in annual costs of health care use per worker from hypothetical improvements (observed to most desirable) of specific psychosocial work environment factors. Parametric g-formula analyses with adjustment for sociodemographic characteristics, job characteristics, physical work environment characteristics, health status and health behaviors.

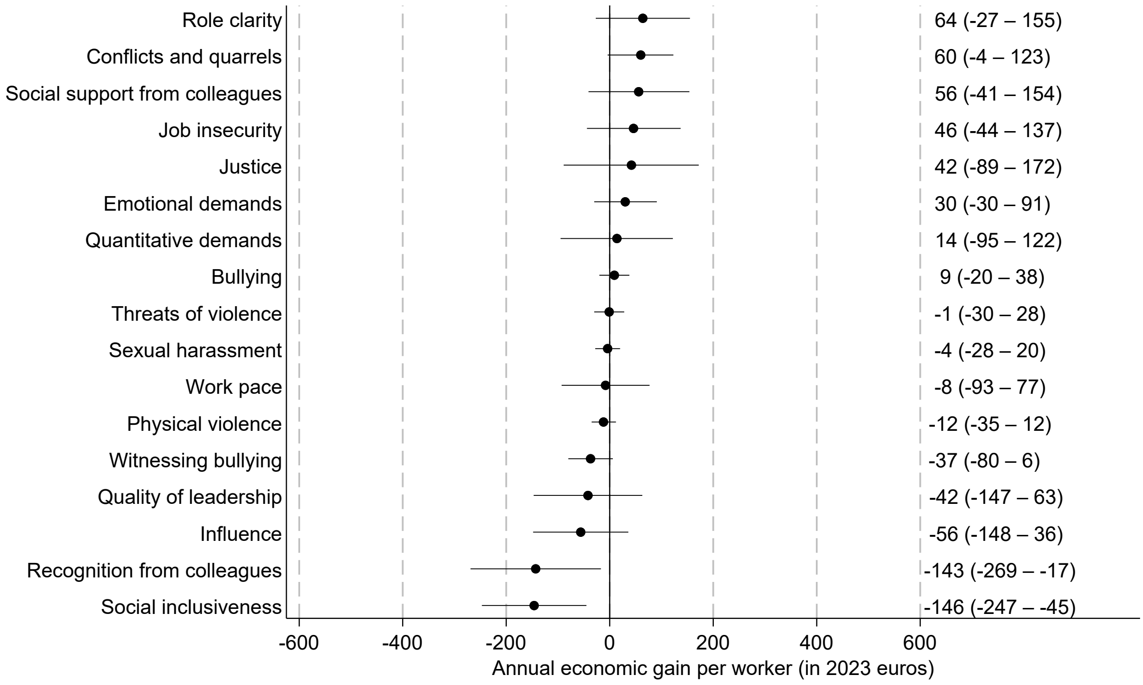

Figure S30. Manufacturing, mining and quarrying. Estimates with 95% confidence intervals for annual economic gains per worker from hypothetical improvements (least to most desirable) of specific psychosocial work environment factors. Parametric g-formula analyses with adjustment for sociodemographic characteristics, job characteristics, physical work environment characteristics, health status and health behaviors.

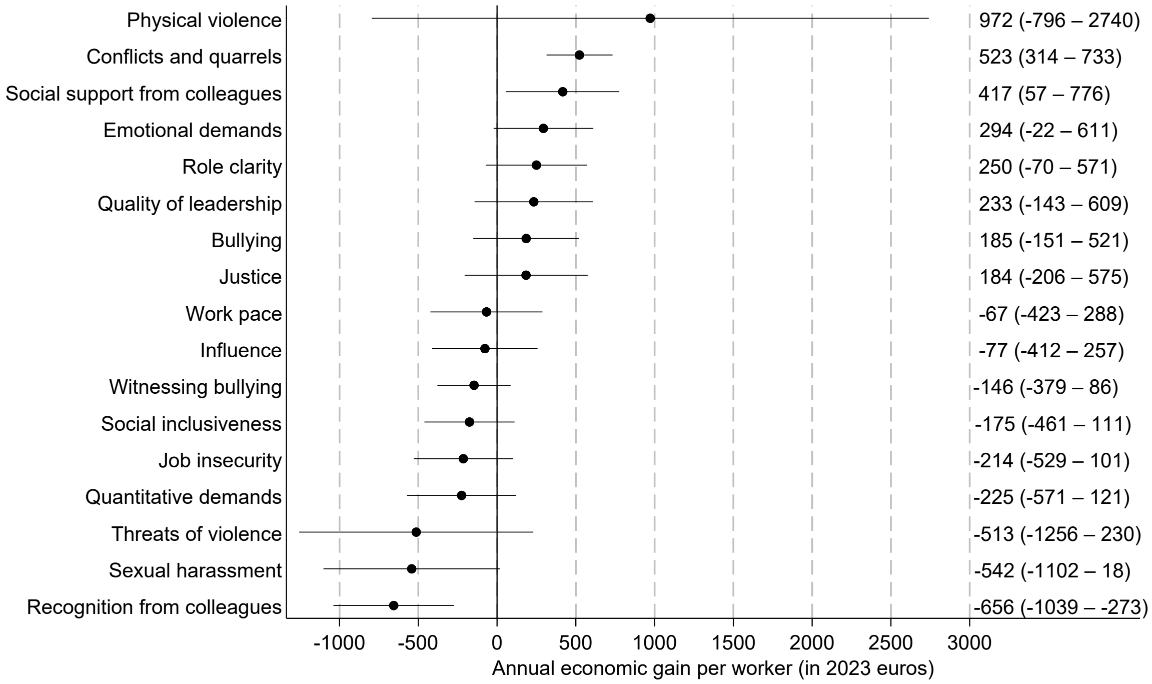

Figure S31. Manufacturing, mining and quarrying. Estimates with 95% confidence intervals for annual economic gains per worker from hypothetical improvements (observed to most desirable) of specific psychosocial work environment factors. Parametric g-formula analyses with adjustment for sociodemographic characteristics, job characteristics, physical work environment characteristics, health status and health behaviors.

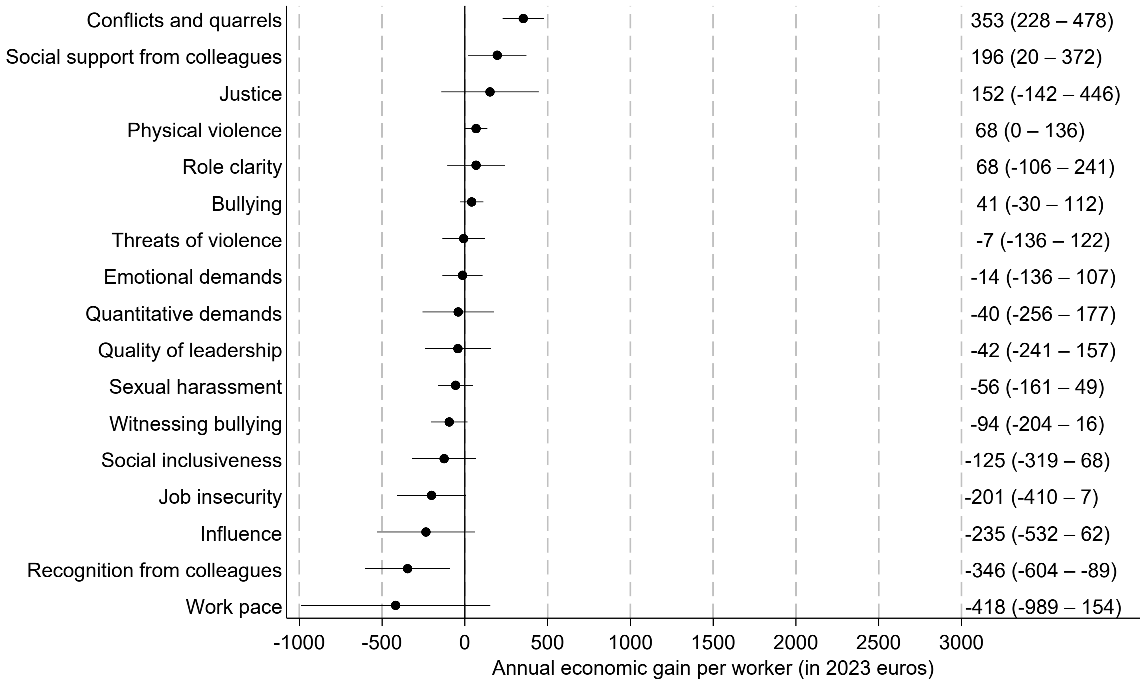

Figure S32. Manufacturing, mining and quarrying. Estimates with 95% confidence intervals for reductions in annual costs of sickness absence per worker from hypothetical improvements (least to most desirable) of specific psychosocial work environment factors. Parametric g-formula analyses with adjustment for sociodemographic characteristics, job characteristics, physical work environment characteristics, health status and health behaviors.

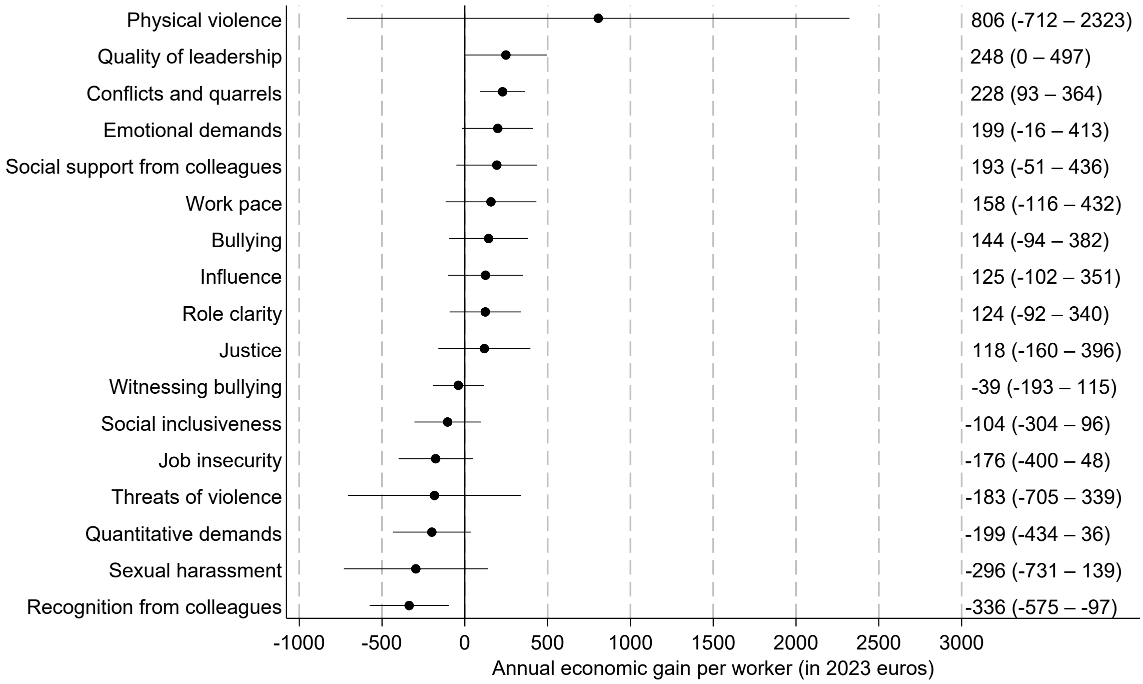

Figure S33. Manufacturing, mining and quarrying. Estimates with 95% confidence intervals for reductions in annual costs of sickness absence per worker from hypothetical improvements (observed to most desirable) of specific psychosocial work environment factors. Parametric g-formula analyses with adjustment for sociodemographic characteristics, job characteristics, physical work environment characteristics, health status and health behaviors.

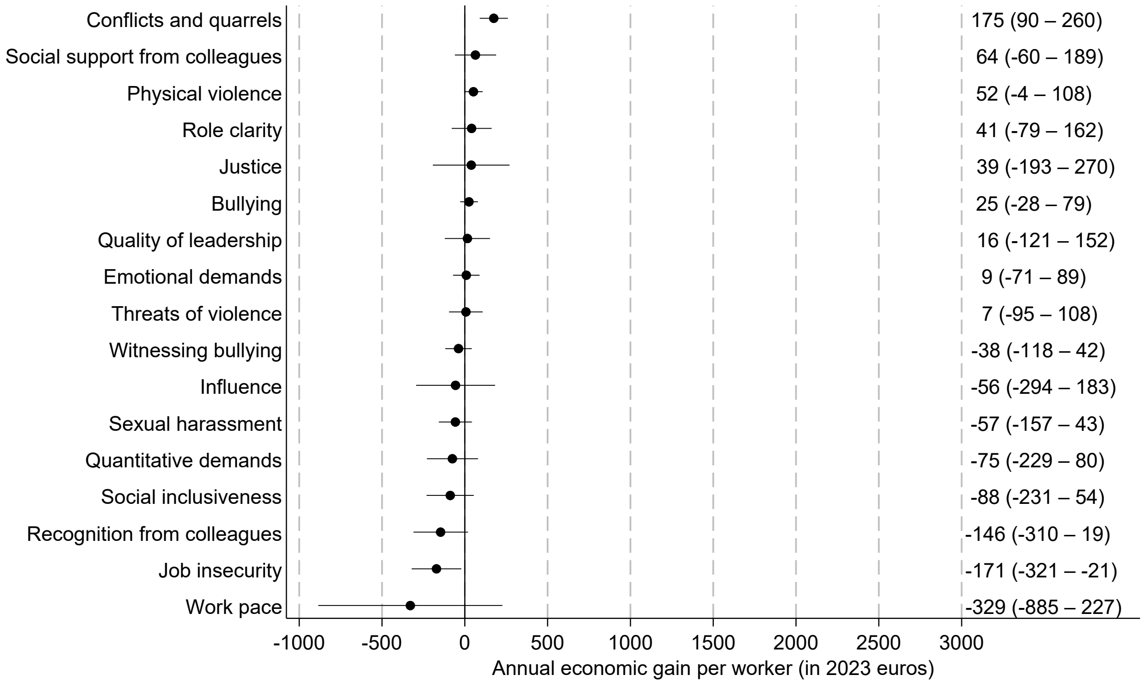

Figure S34. Manufacturing, mining and quarrying. Estimates with 95% confidence intervals for reductions in annual costs of health care use per worker from hypothetical improvements (least to most desirable) of specific psychosocial work environment factors. Parametric g-formula analyses with adjustment for sociodemographic characteristics, job characteristics, physical work environment characteristics, health status and health behaviors.

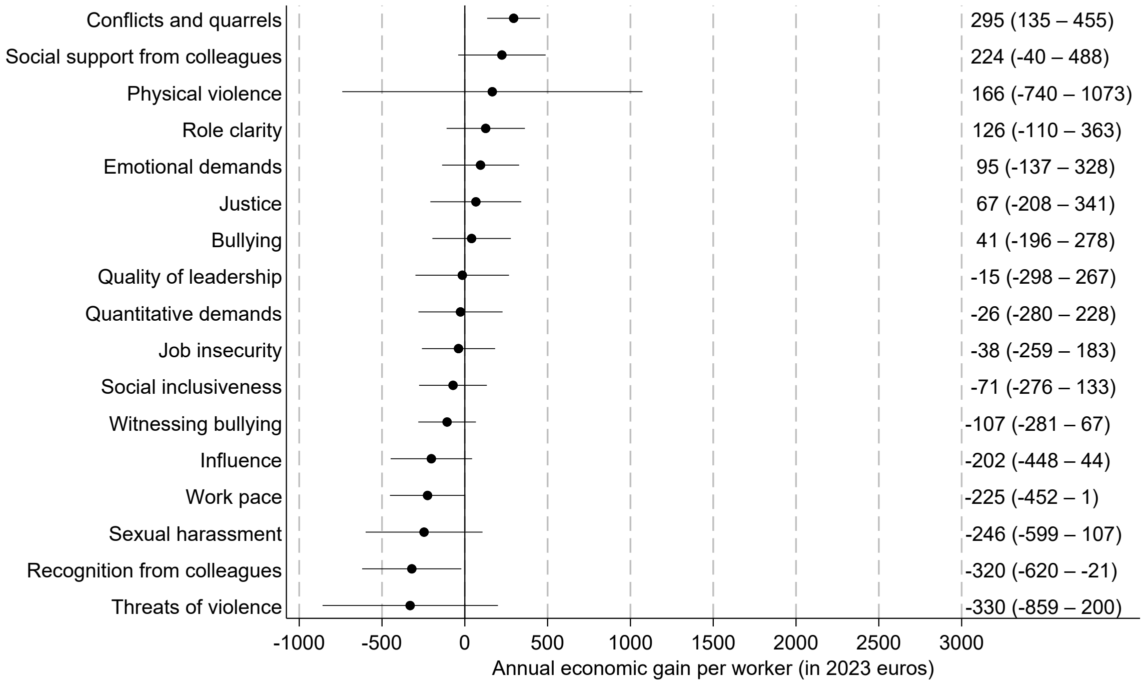

Figure S35. Manufacturing, mining and quarrying. Estimates with 95% confidence intervals for reductions in annual costs of health care use per worker from hypothetical improvements (observed to most desirable) of specific psychosocial work environment factors. Parametric g-formula analyses with adjustment for sociodemographic characteristics, job characteristics, physical work environment characteristics, health status and health behaviors.

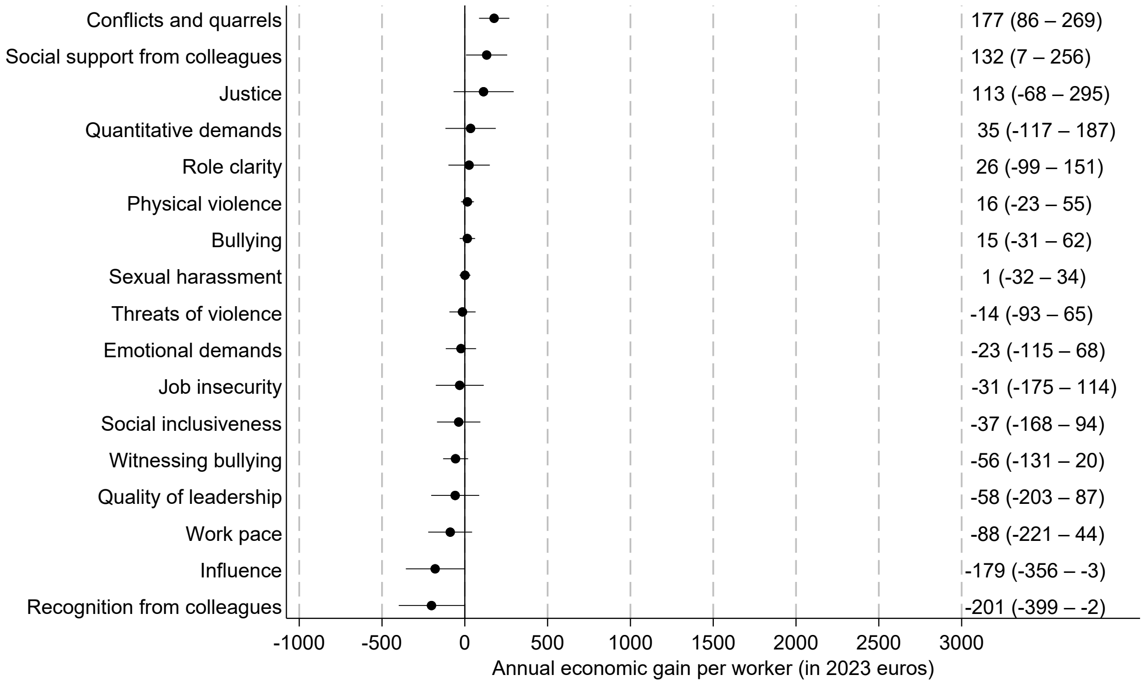

Figure S36. Public administration, defence and police. Estimates with 95% confidence intervals for annual economic gains per worker from hypothetical improvements (least to most desirable) of specific psychosocial work environment factors. Parametric g-formula analyses with adjustment for sociodemographic characteristics, job characteristics, physical work environment characteristics, health status and health behaviors.

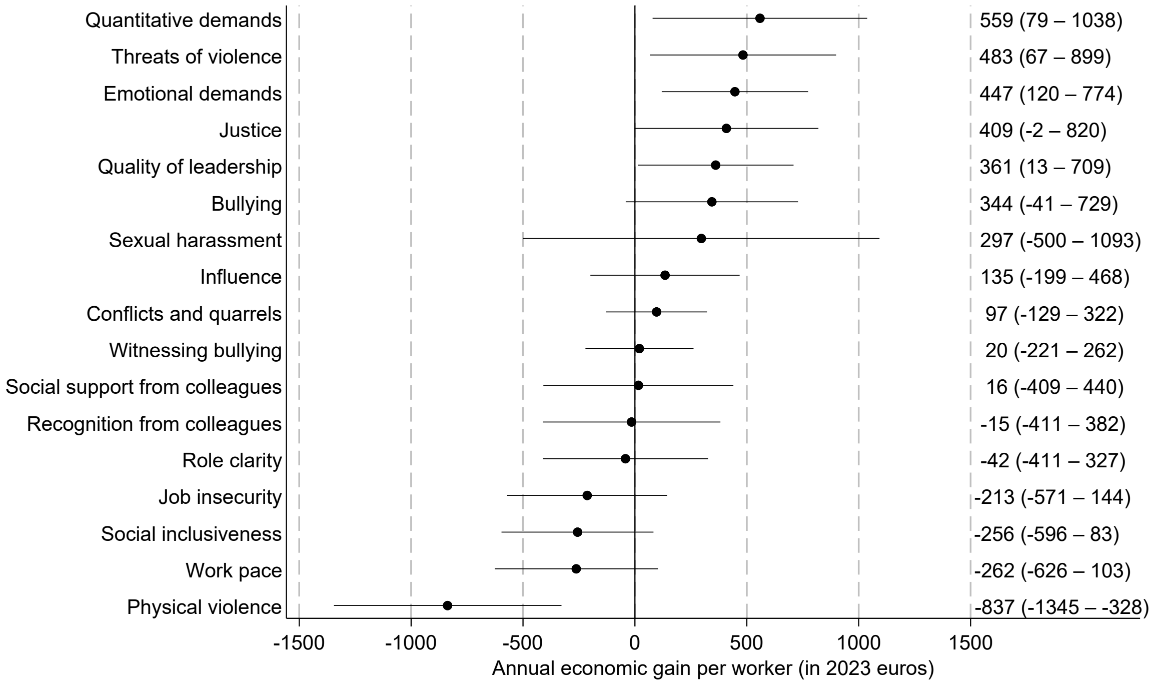

Figure S37. Public administration, defence and police. Estimates with 95% confidence intervals for annual economic gains per worker from hypothetical improvements (observed to most desirable) of specific psychosocial work environment factors. Parametric g-formula analyses with adjustment for sociodemographic characteristics, job characteristics, physical work environment characteristics, health status and health behaviors.

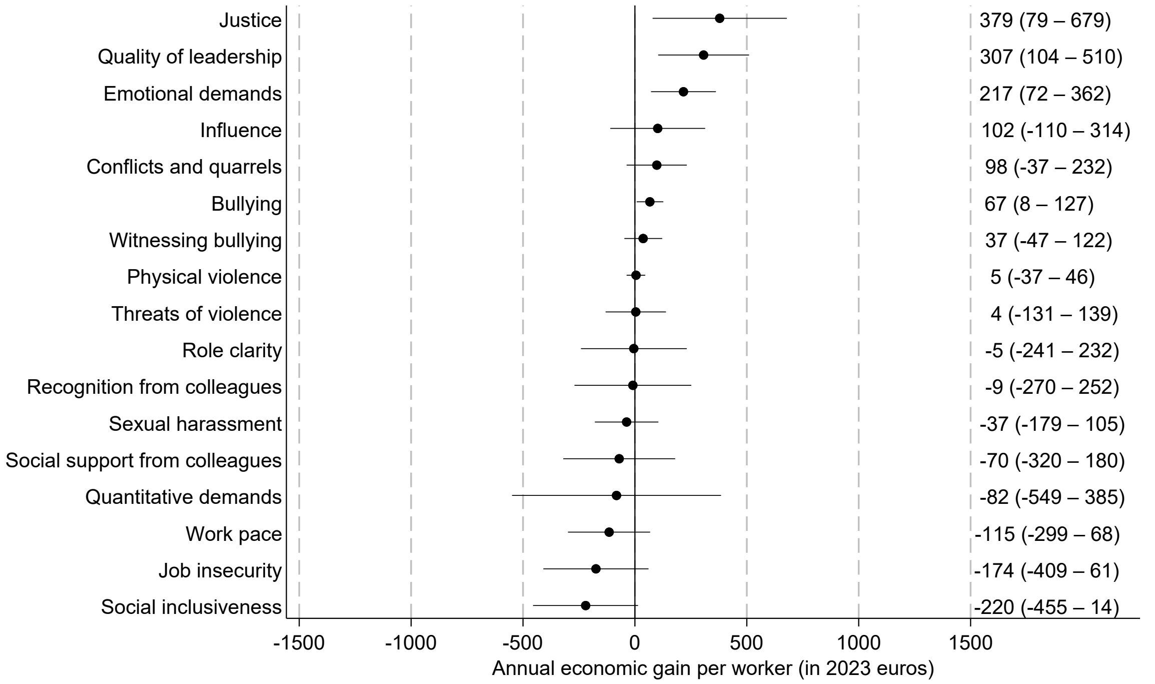

Figure S38. Public administration, defence and police. Estimates with 95% confidence intervals for reductions in annual costs of sickness absence per worker from hypothetical improvements (least to most desirable) of specific psychosocial work environment factors. Parametric g-formula analyses with adjustment for sociodemographic characteristics, job characteristics, physical work environment characteristics, health status and health behaviors.

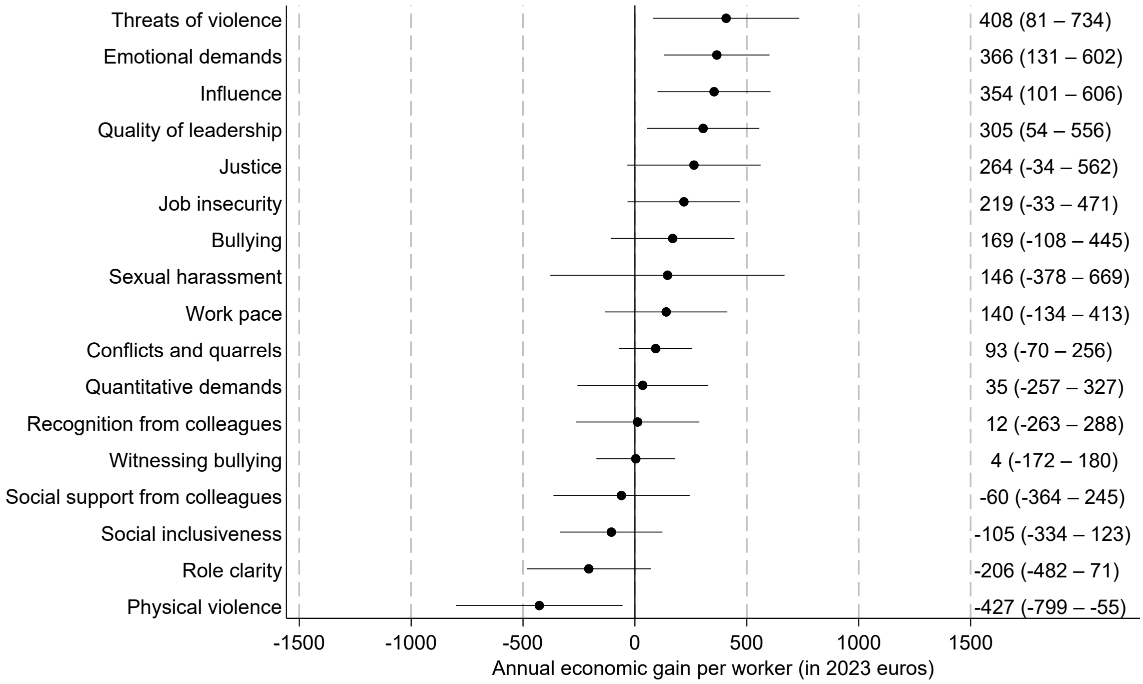

Figure S39. Public administration, defence and police. Estimates with 95% confidence intervals for reductions in annual costs of sickness absence per worker from hypothetical improvements (observed to most desirable) of specific psychosocial work environment factors. Parametric g-formula analyses with adjustment for sociodemographic characteristics, job characteristics, physical work environment characteristics, health status and health behaviors.

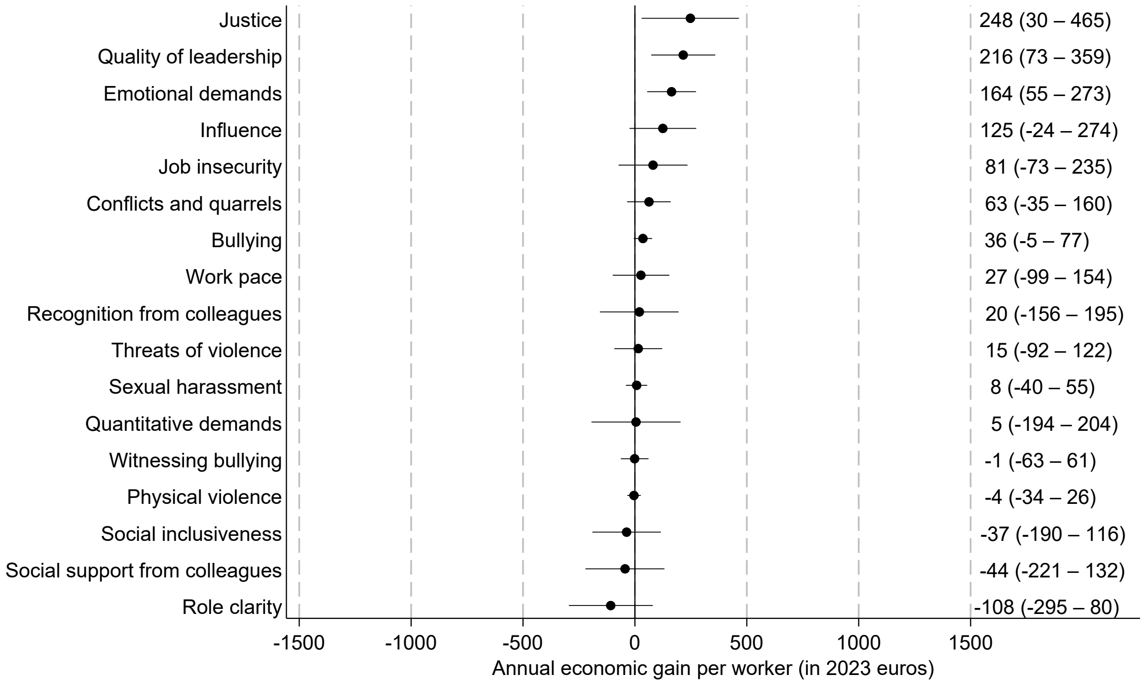

Figure S40. Public administration, defence and police. Estimates with 95% confidence intervals for reductions in annual costs of health care use per worker from hypothetical improvements (least to most desirable) of specific psychosocial work environment factors. Parametric g-formula analyses with adjustment for sociodemographic characteristics, job characteristics, physical work environment characteristics, health status and health behaviors.

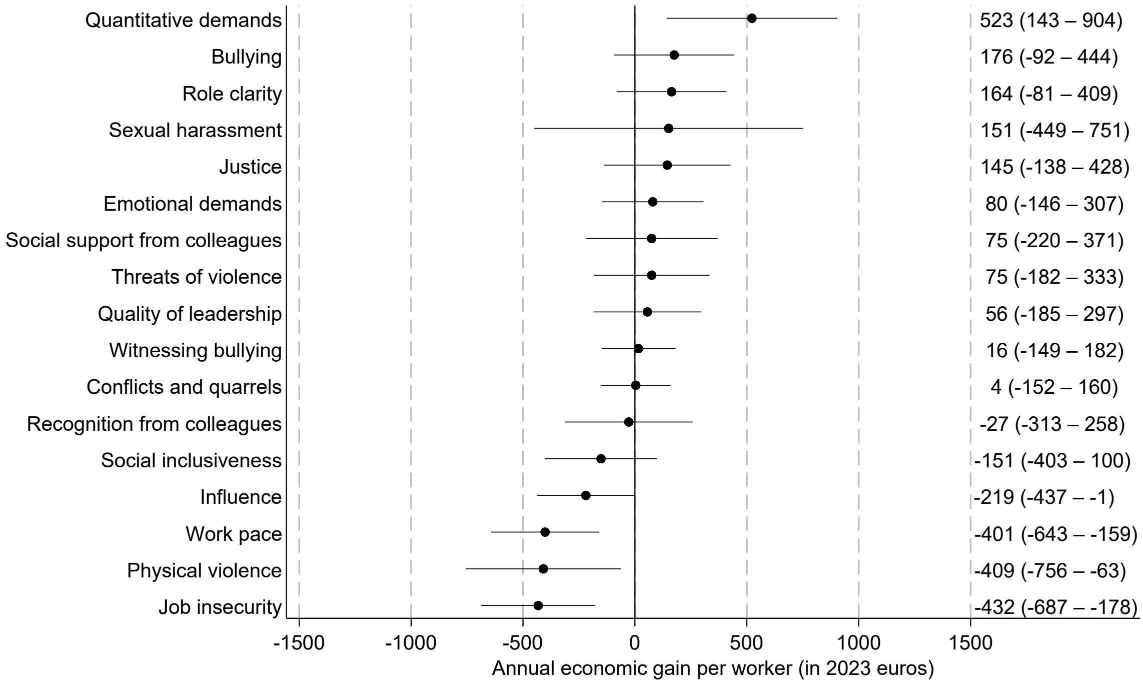

Figure S41. Public administration, defence and police. Estimates with 95% confidence intervals for reductions in annual costs of health care use per worker from hypothetical improvements (observed to most desirable) of specific psychosocial work environment factors. Parametric g-formula analyses with adjustment for sociodemographic characteristics, job characteristics, physical work environment characteristics, health status and health behaviors.

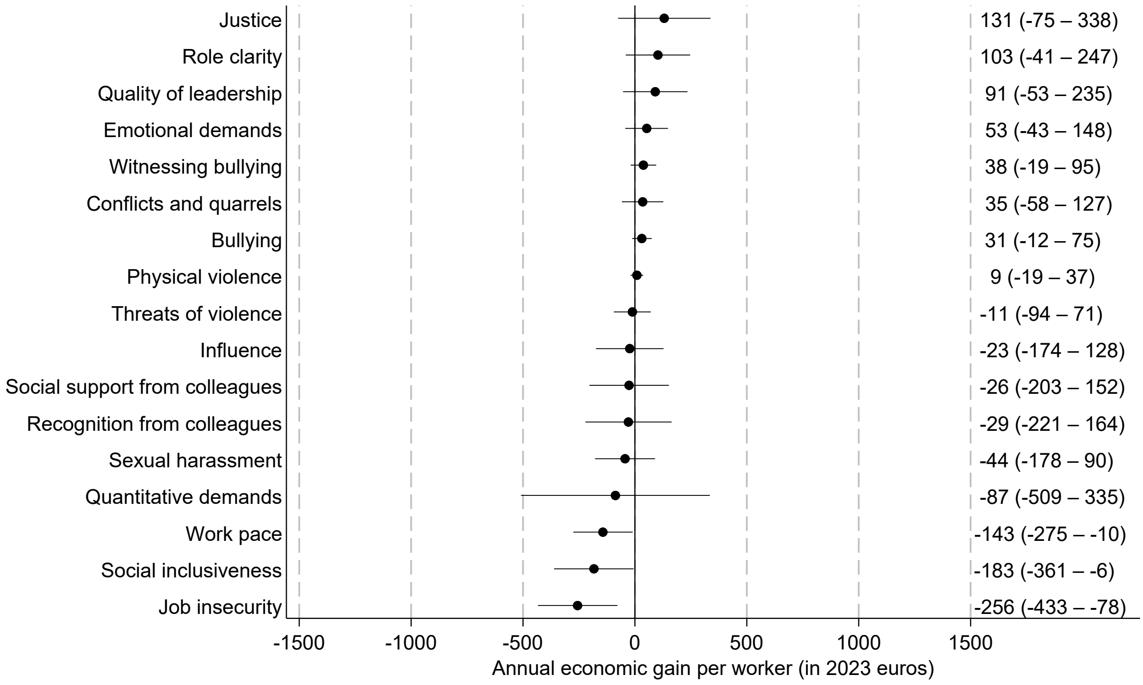

Education

For the industry labelled "Education", we were not able to estimate the hurdle model for health care use costs. Therefore, for this industry, we only include figures showing estimates for reductions in annual costs of sickness absence per worker from hypothetical improvements of specific psychosocial work environment factors. The figures are placed on the next page.

Figure S42. Education. Estimates with 95% confidence intervals for reductions in annual costs of sickness absence per worker from hypothetical improvements (least to most desirable) of specific psychosocial work environment factors. Parametric g-formula analyses with adjustment for sociodemographic characteristics, job characteristics, physical work environment characteristics, health status and health behaviors.

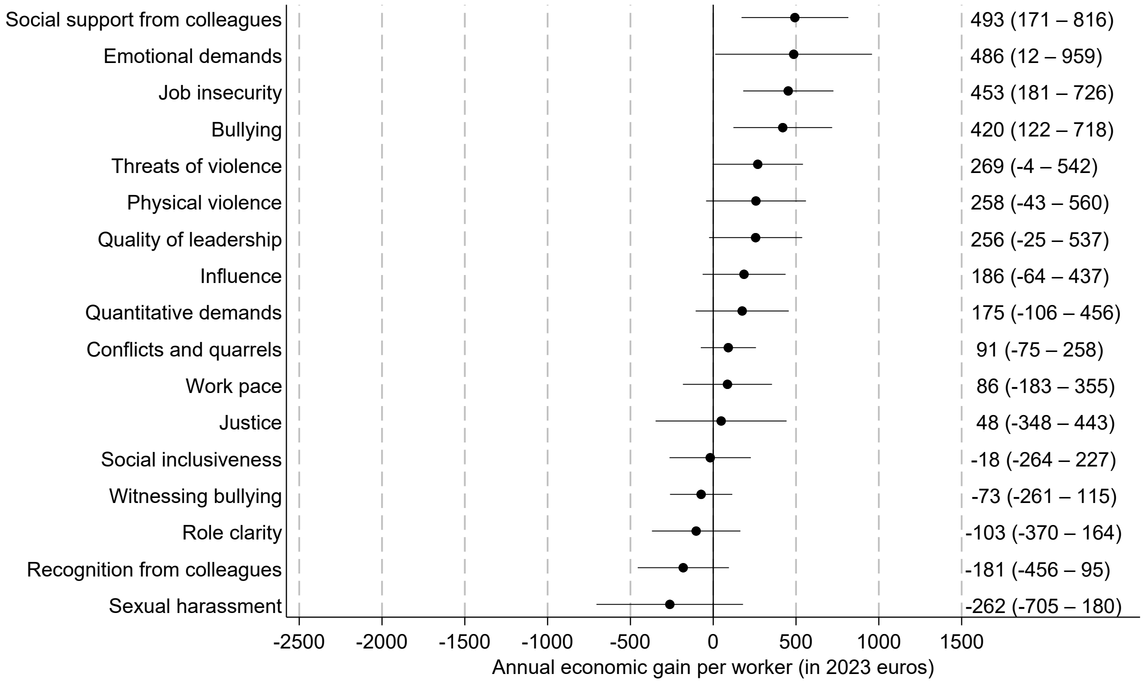

Figure S43. Education. Estimates with 95% confidence intervals for reductions in annual costs of sickness absence per worker from hypothetical improvements (observed to most desirable) of specific psychosocial work environment factors. Parametric g-formula analyses with adjustment for sociodemographic characteristics, job characteristics, physical work environment characteristics, health status and health behaviors.

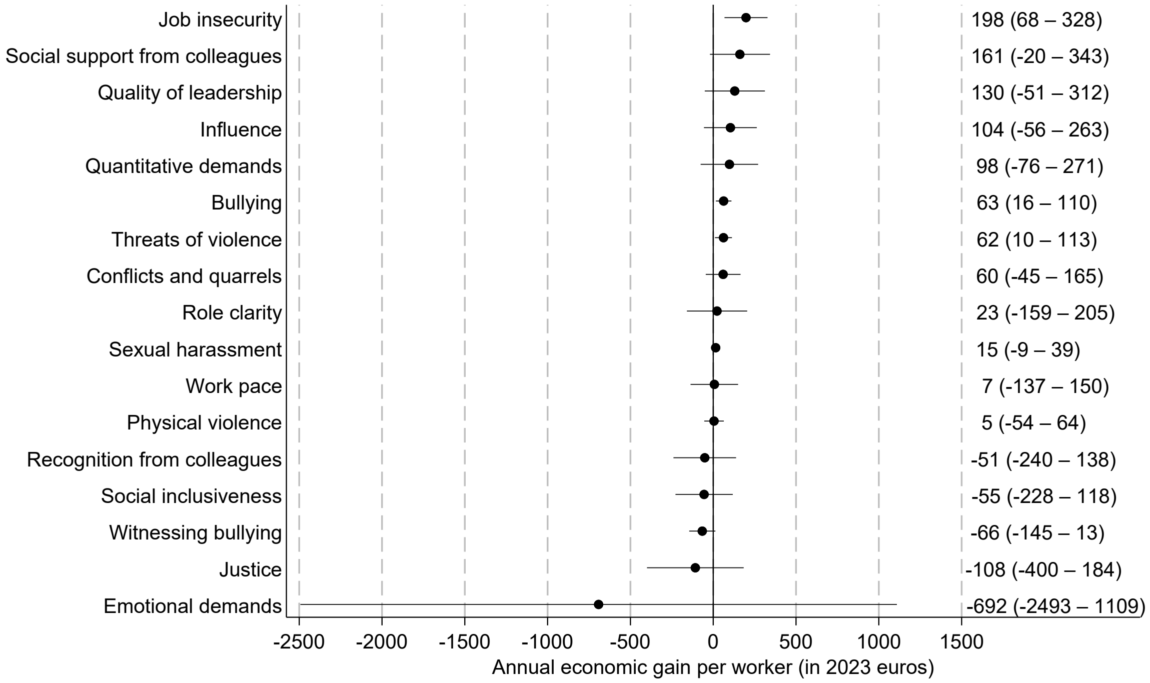

Figure S44. Human health activities. Estimates with 95% confidence intervals for annual economic gains per worker from hypothetical improvements (least to most desirable) of specific psychosocial work environment factors. Parametric g-formula analyses with adjustment for sociodemographic characteristics, job characteristics, physical work environment characteristics, health status and health behaviors.

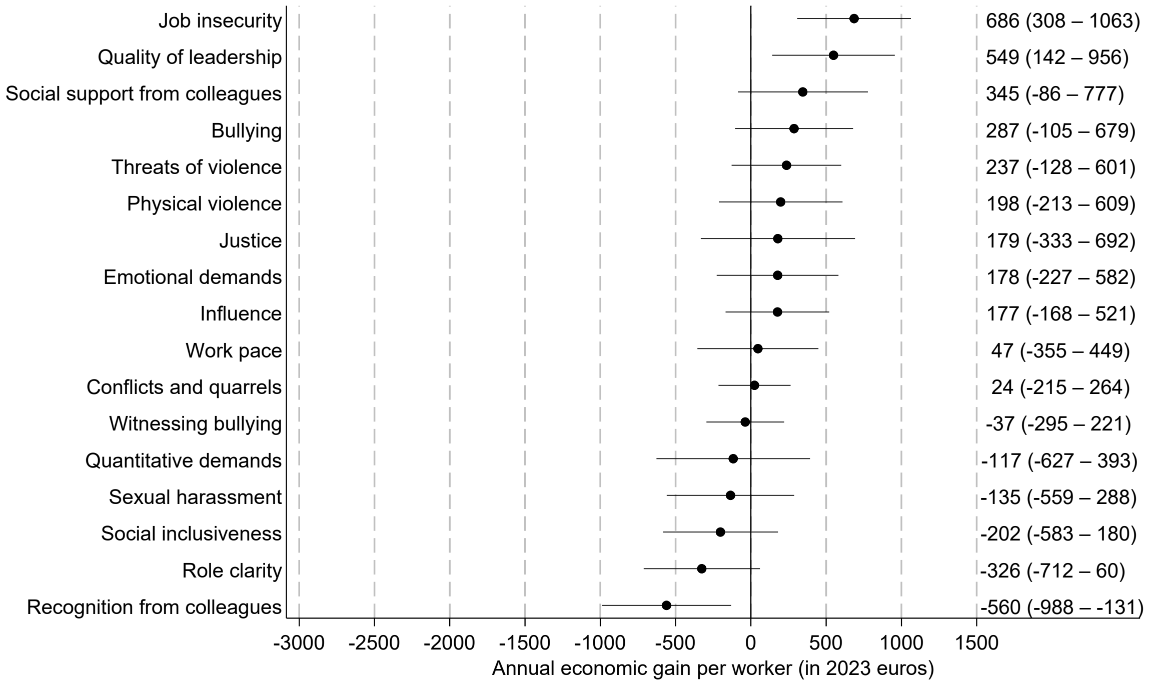

Figure S45. Human health activities. Estimates with 95% confidence intervals for annual economic gains per worker from hypothetical improvements (observed to most desirable) of specific psychosocial work environment factors. Parametric g-formula analyses with adjustment for sociodemographic characteristics, job characteristics, physical work environment characteristics, health status and health behaviors.

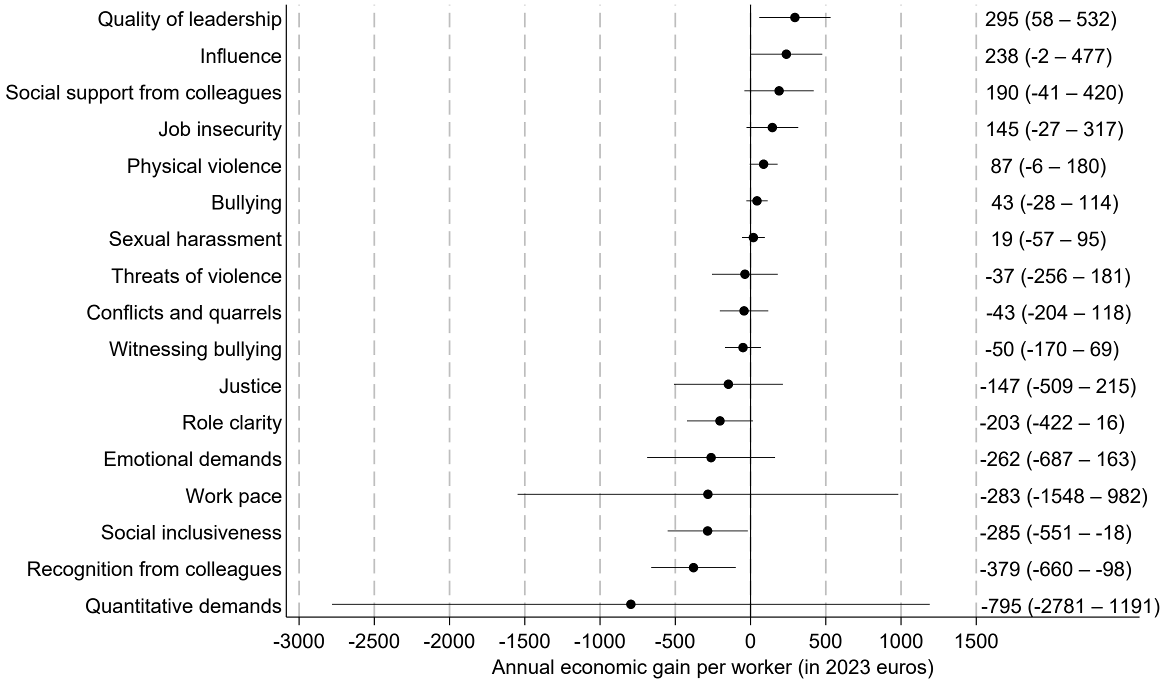

Figure S46. Human health activities. Estimates with 95% confidence intervals for reductions in annual costs of sickness absence per worker from hypothetical improvements (least to most desirable) of specific psychosocial work environment factors. Parametric g-formula analyses with adjustment for sociodemographic characteristics, job characteristics, physical work environment characteristics, health status and health behaviors.

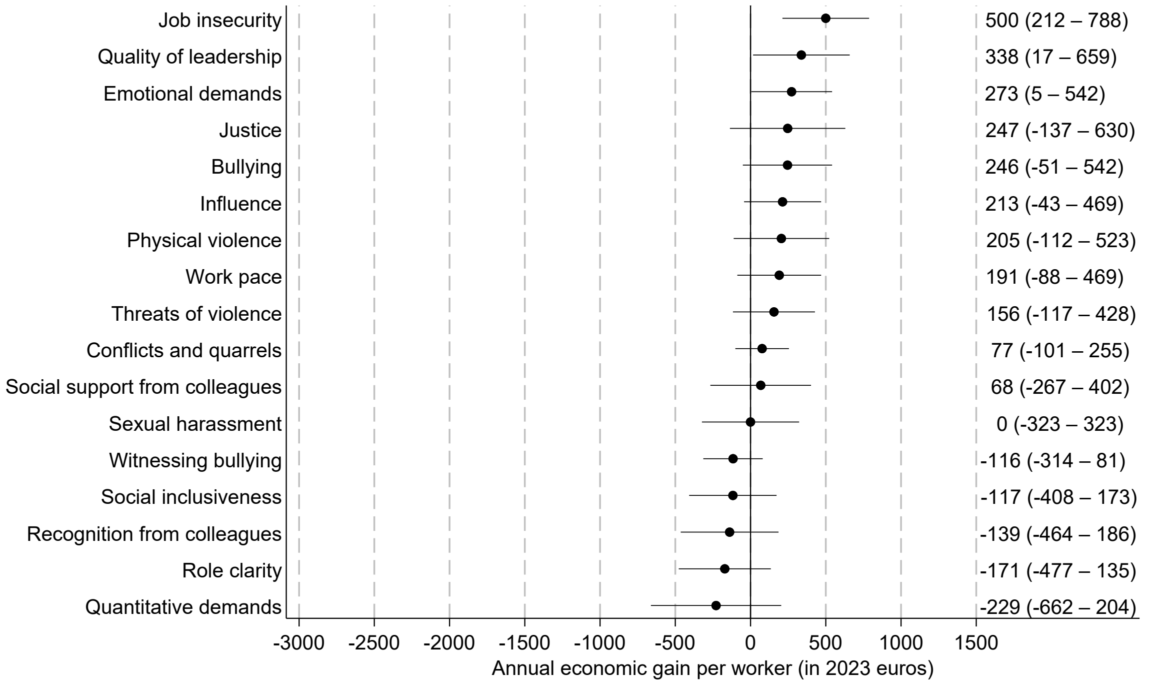

Figure S47. Human health activities. Estimates with 95% confidence intervals for reductions in annual costs of sickness absence per worker from hypothetical improvements (observed to most desirable) of specific psychosocial work environment factors. Parametric g-formula analyses with adjustment for sociodemographic characteristics, job characteristics, physical work environment characteristics, health status and health behaviors.

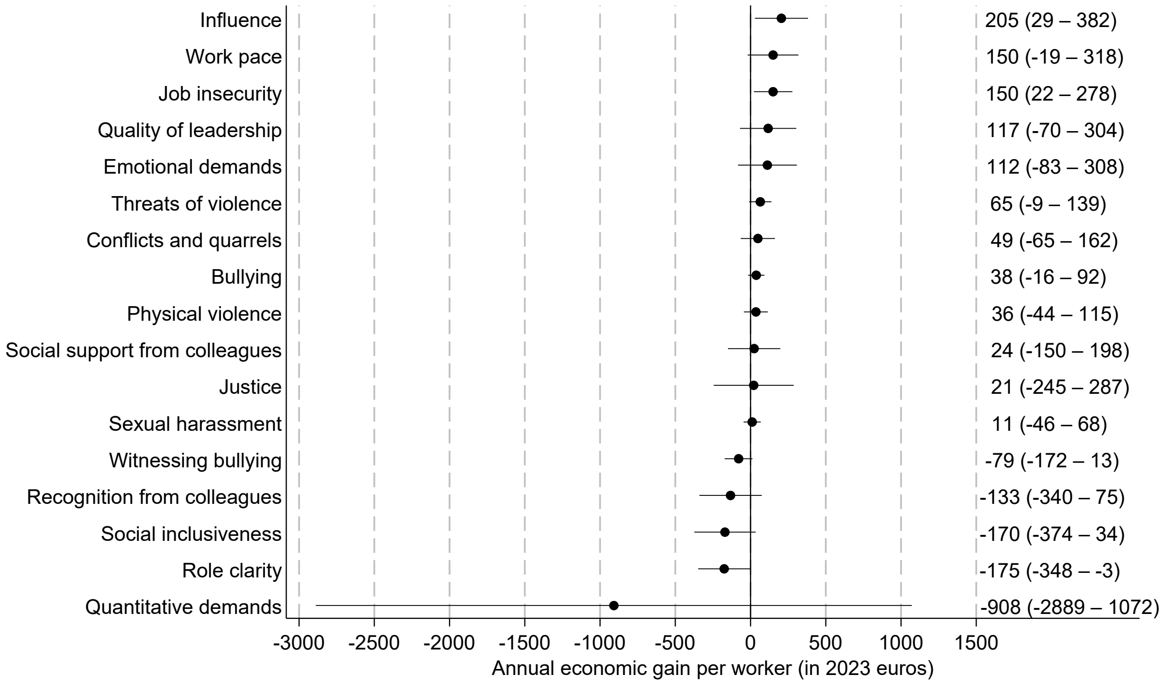

Figure S48. Human health activities. Estimates with 95% confidence intervals for reductions in annual costs of health care use per worker from hypothetical improvements (least to most desirable) of specific psychosocial work environment factors. Parametric g-formula analyses with adjustment for sociodemographic characteristics, job characteristics, physical work environment characteristics, health status and health behaviors.

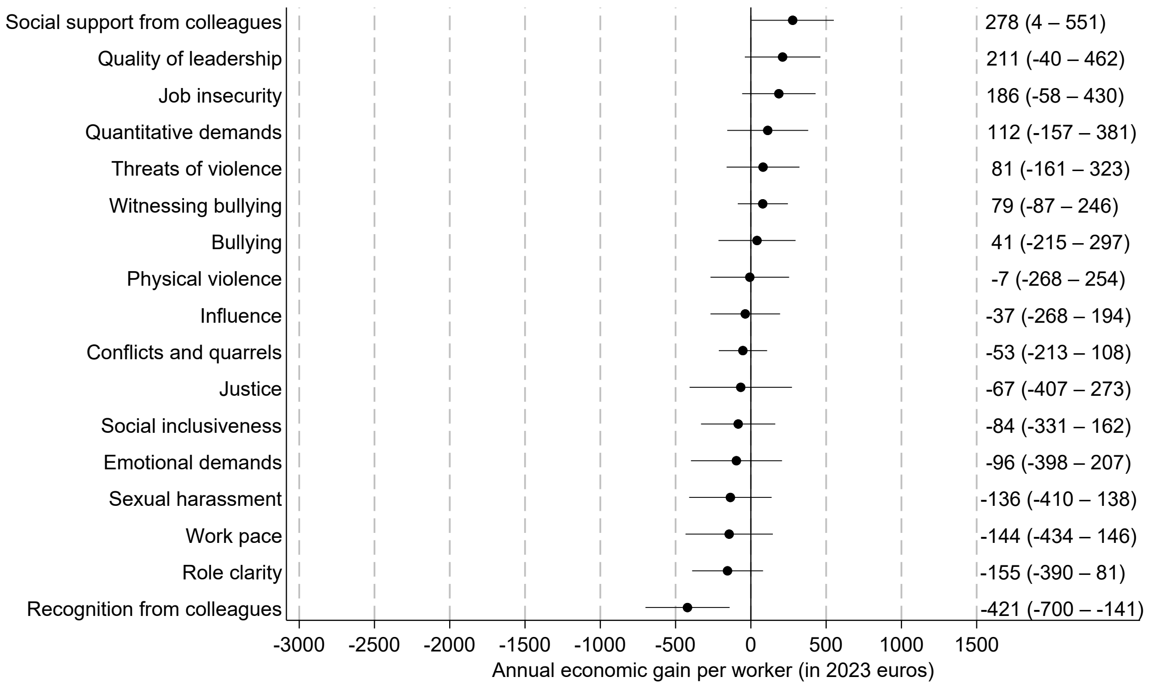

Figure S49. Human health activities. Estimates with 95% confidence intervals for reductions in annual costs of health care use per worker from hypothetical improvements (observed to most desirable) of specific psychosocial work environment factors. Parametric g-formula analyses with adjustment for sociodemographic characteristics, job characteristics, physical work environment characteristics, health status and health behaviors.

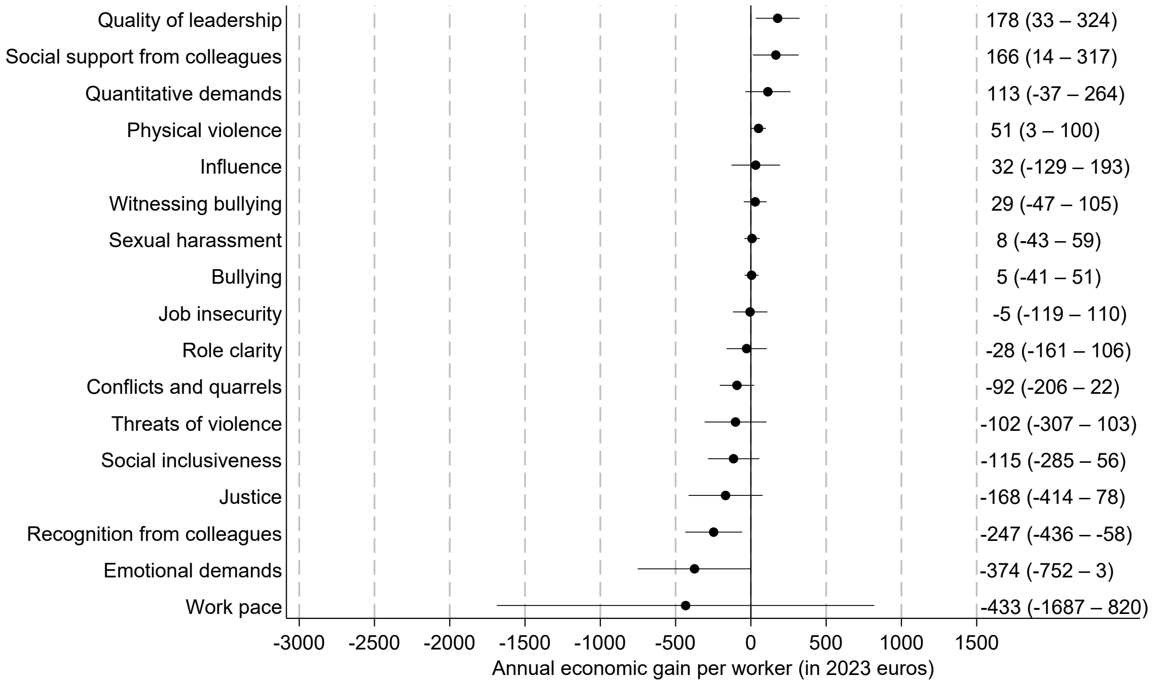

Figure S50. Social institutions. Estimates with 95% confidence intervals for annual economic gains per worker from hypothetical improvements (least to most desirable) of specific psychosocial work environment factors. Parametric g-formula analyses with adjustment for sociodemographic characteristics, job characteristics, physical work environment characteristics, health status and health behaviors.

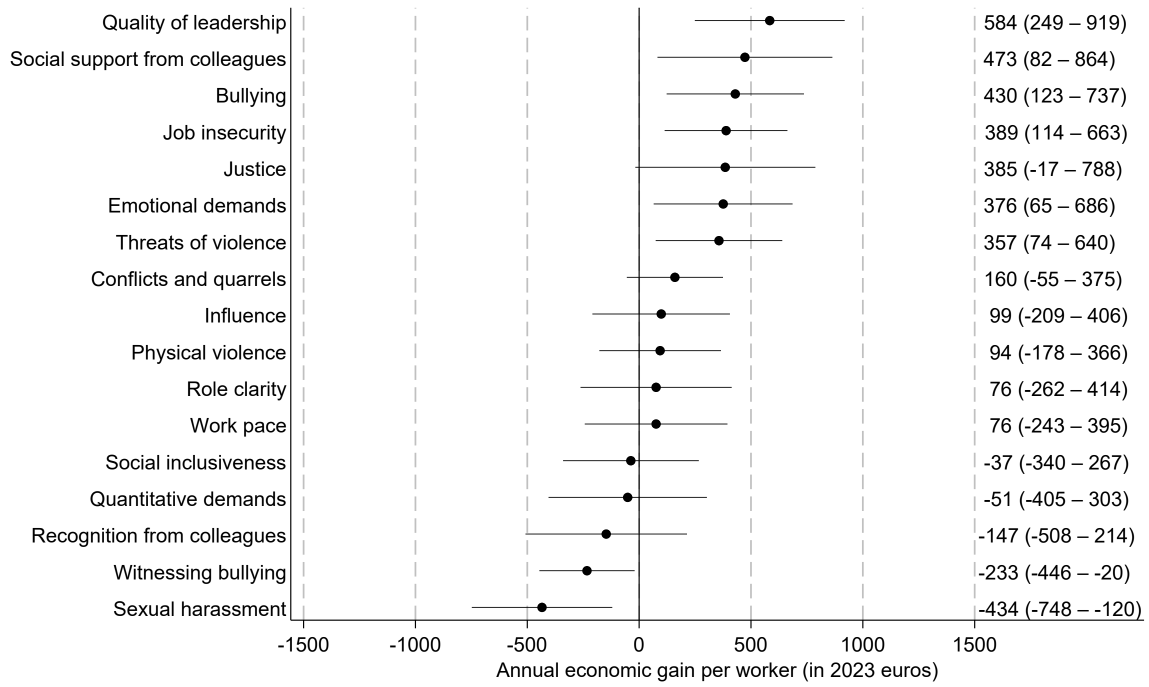

Figure S51. Social institutions. Estimates with 95% confidence intervals for annual economic gains per worker from hypothetical improvements (observed to most desirable) of specific psychosocial work environment factors. Parametric g-formula analyses with adjustment for sociodemographic characteristics, job characteristics, physical work environment characteristics, health status and health behaviors.

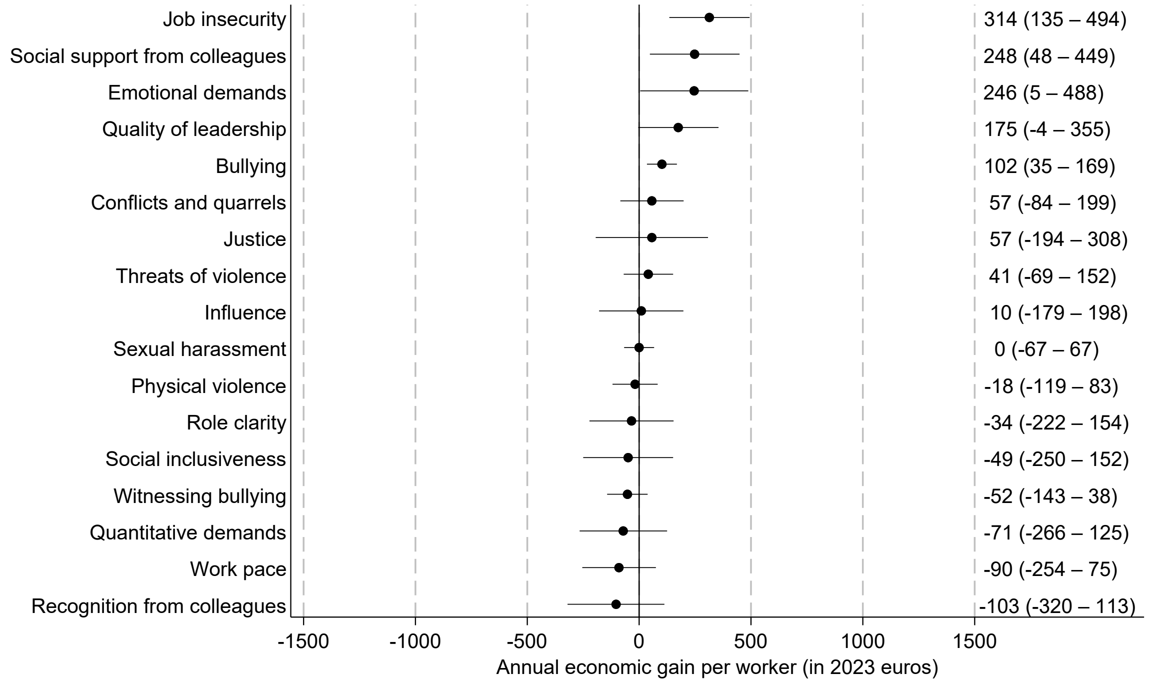

Figure S52. Social institutions. Estimates with 95% confidence intervals for reductions in annual costs of sickness absence per worker from hypothetical improvements (least to most desirable) of specific psychosocial work environment factors. Parametric g-formula analyses with adjustment for sociodemographic characteristics, job characteristics, physical work environment characteristics, health status and health behaviors.

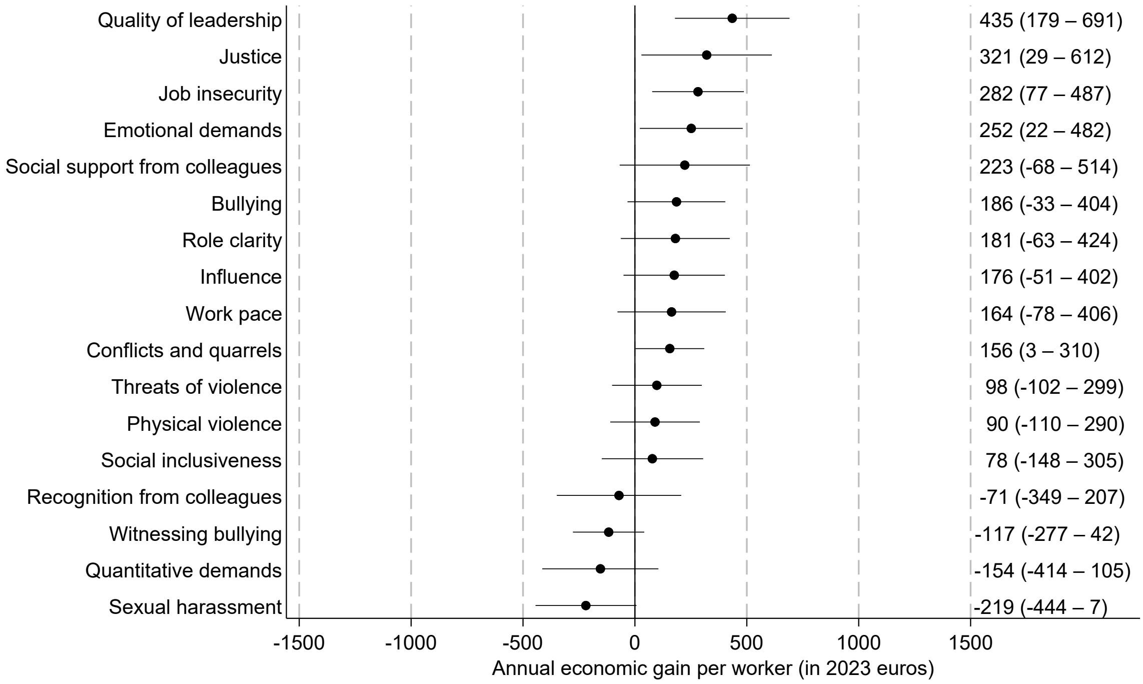

Figure S53. Social institutions. Estimates with 95% confidence intervals for reductions in annual costs of sickness absence per worker from hypothetical improvements (observed to most desirable) of specific psychosocial work environment factors. Parametric g-formula analyses with adjustment for sociodemographic characteristics, job characteristics, physical work environment characteristics, health status and health behaviors.

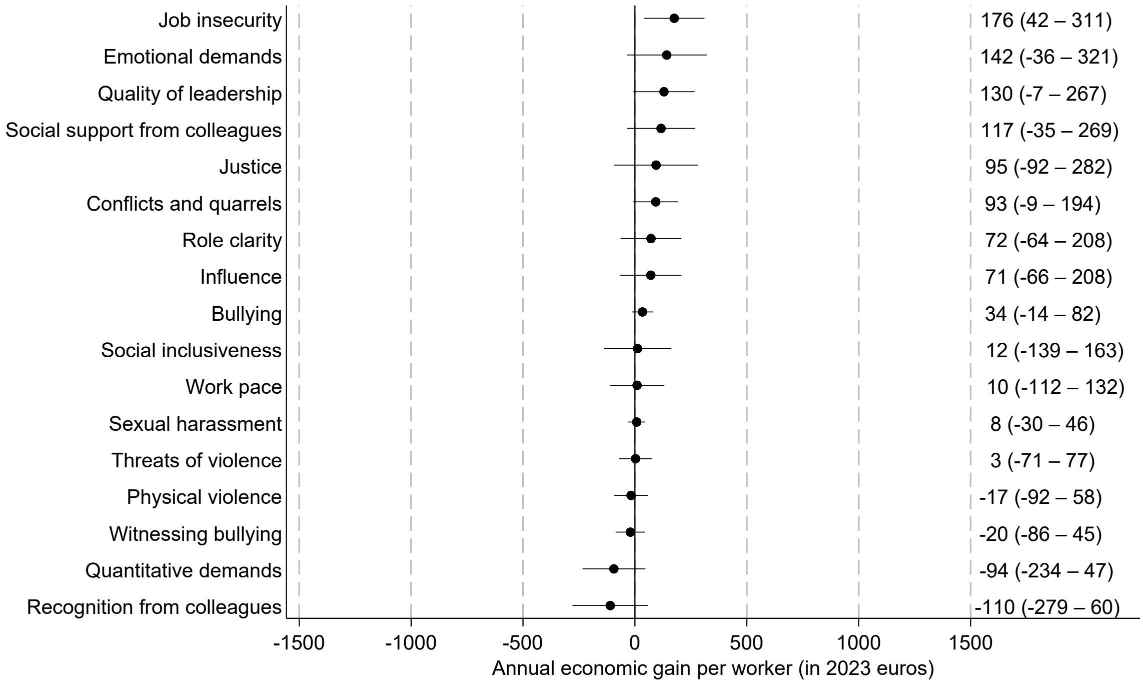

Figure S54. Social institutions. Estimates with 95% confidence intervals for reductions in annual costs of health care use per worker from hypothetical improvements (least to most desirable) of specific psychosocial work environment factors. Parametric g-formula analyses with adjustment for sociodemographic characteristics, job characteristics, physical work environment characteristics, health status and health behaviors.

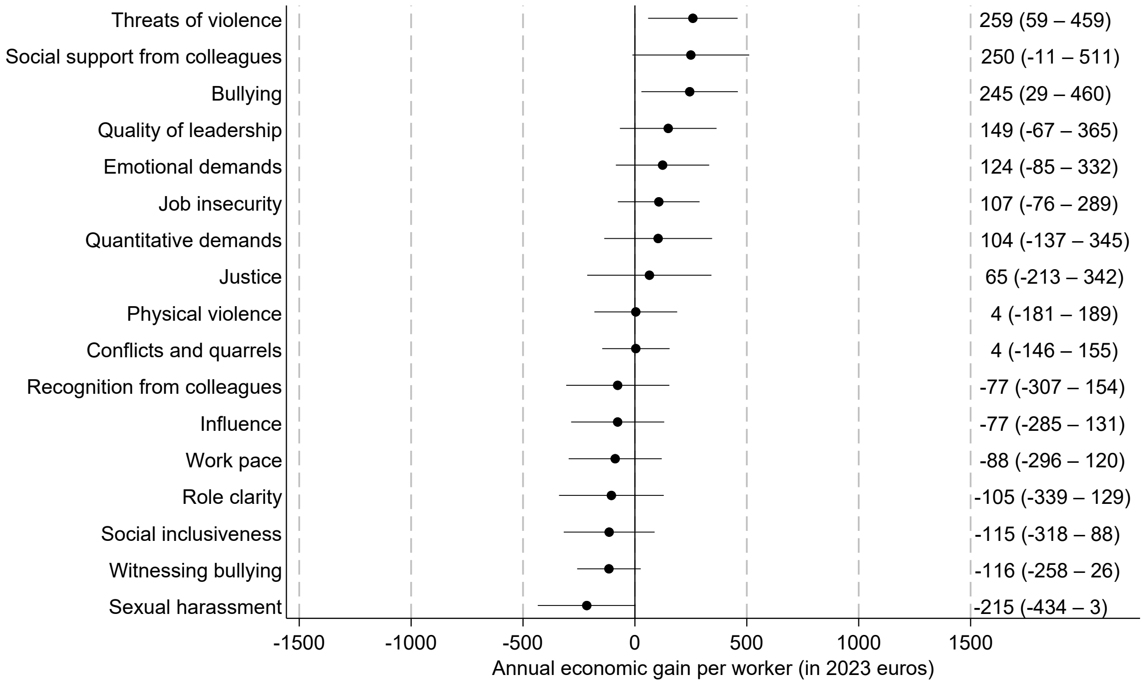

Figure S55. Social institutions. Estimates with 95% confidence intervals for reductions in annual costs of health care use per worker from hypothetical improvements (observed to most desirable) of specific psychosocial work environment factors. Parametric g-formula analyses with adjustment for sociodemographic characteristics, job characteristics, physical work environment characteristics, health status and health behaviors.

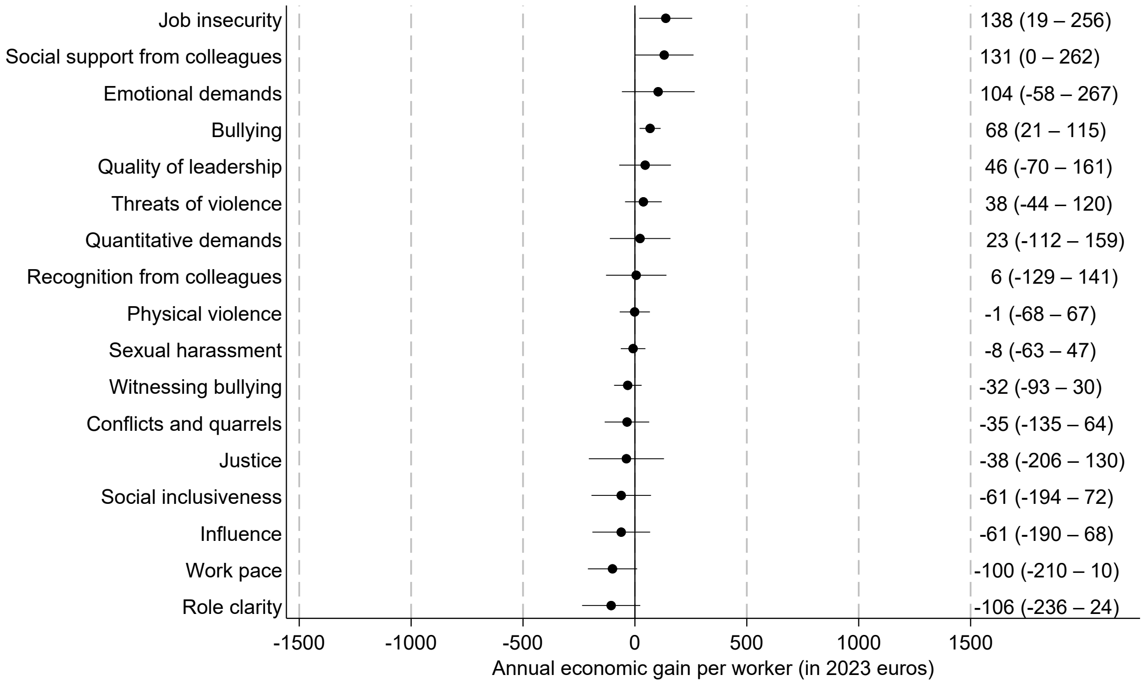

Figure S56. Workers not employed in social institutions. Estimates with 95% confidence intervals for annual economic gains per worker from hypothetical improvements (least to most desirable) of specific psychosocial work environment factors. Parametric g-formula analyses with adjustment for sociodemographic characteristics, job characteristics, physical work environment characteristics, health status and health behaviors.

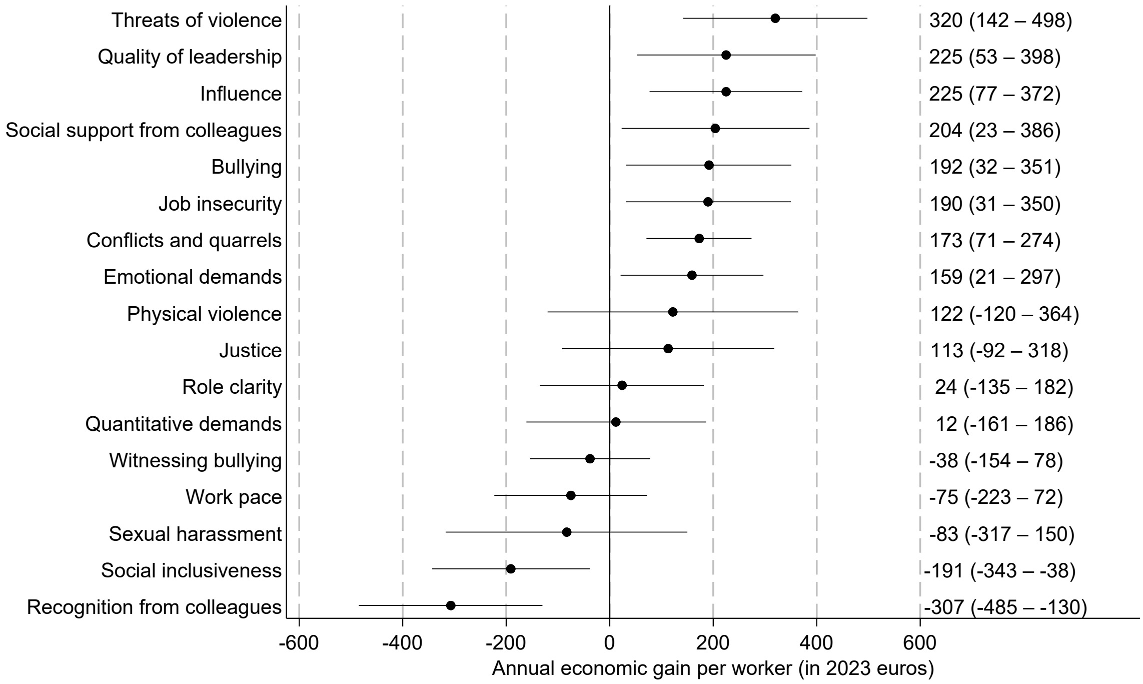

Figure S57. Workers not employed in social institutions. Estimates with 95% confidence intervals for annual economic gains per worker from hypothetical improvements (observed to most desirable) of specific psychosocial work environment factors. Parametric g-formula analyses with adjustment for sociodemographic characteristics, job characteristics, physical work environment characteristics, health status and health behaviors.

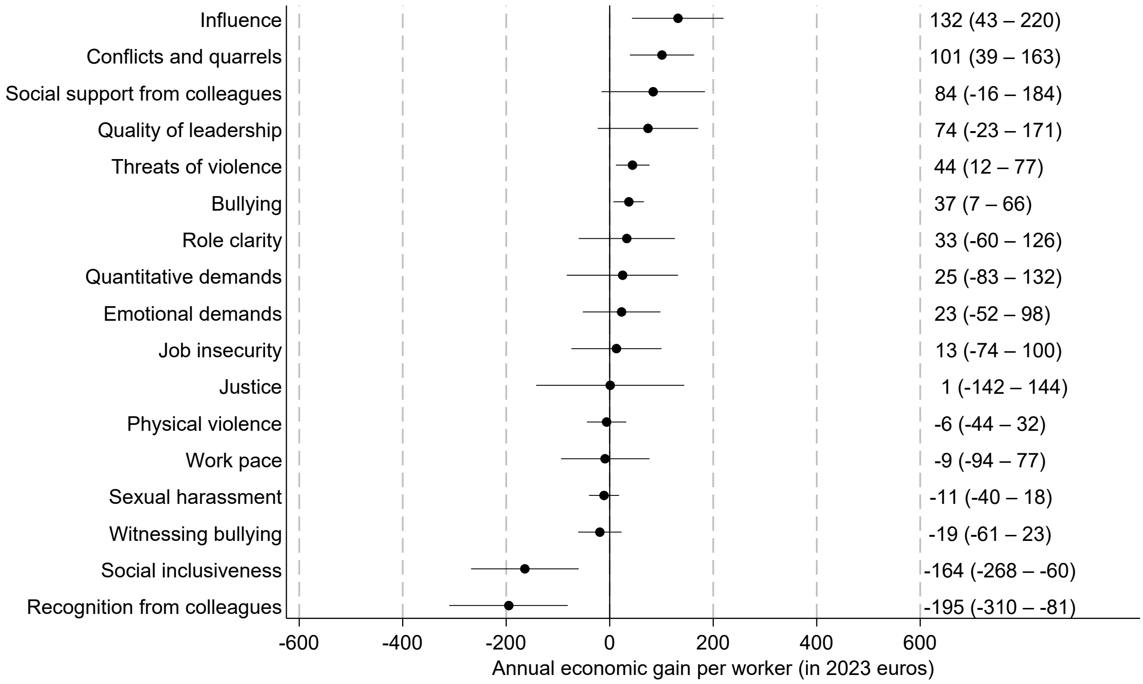

Figure S58. Workers not employed in social institutions. Estimates with 95% confidence intervals for reductions in annual costs of sickness absence per worker from hypothetical improvements (least to most desirable) of specific psychosocial work environment factors. Parametric g-formula analyses with adjustment for sociodemographic characteristics, job characteristics, physical work environment characteristics, health status and health behaviors.

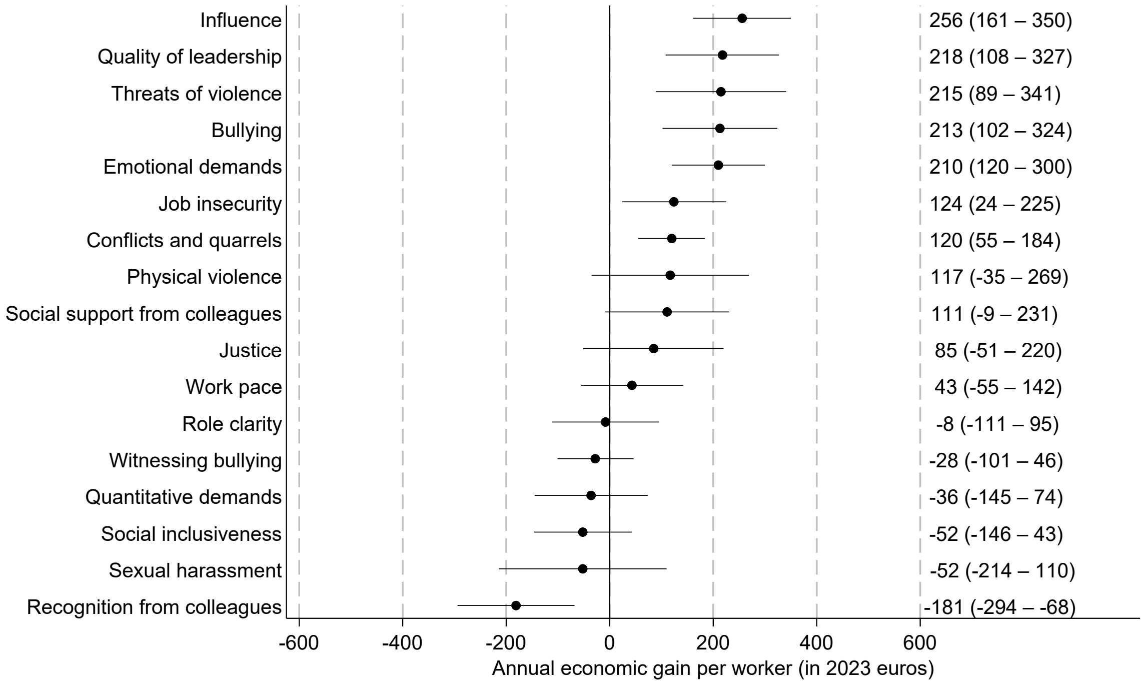

Figure S59. Workers not employed in social institutions. Estimates with 95% confidence intervals for reductions in annual costs of sickness absence per worker from hypothetical improvements (observed to most desirable) of specific psychosocial work environment factors. Parametric g-formula analyses with adjustment for sociodemographic characteristics, job characteristics, physical work environment characteristics, health status and health behaviors.

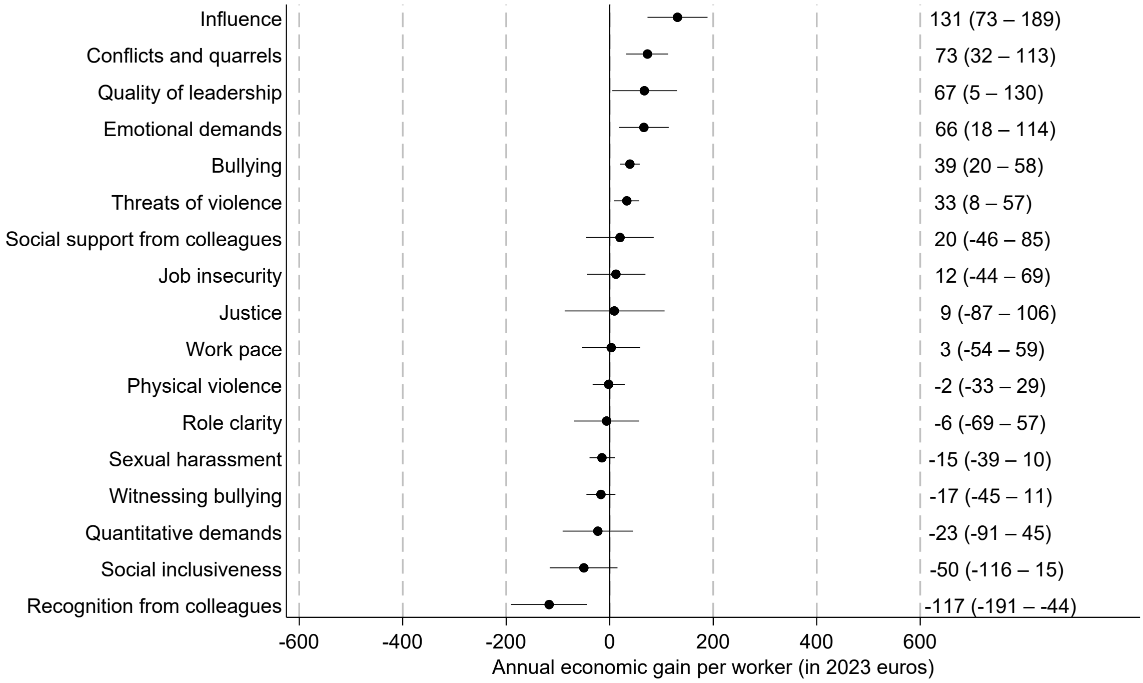

Figure S60. Workers not employed in social institutions. Estimates with 95% confidence intervals for reductions in annual costs of health care use per worker from hypothetical improvements (least to most desirable) of specific psychosocial work environment factors. Parametric g-formula analyses with adjustment for sociodemographic characteristics, job characteristics, physical work environment characteristics, health status and health behaviors.

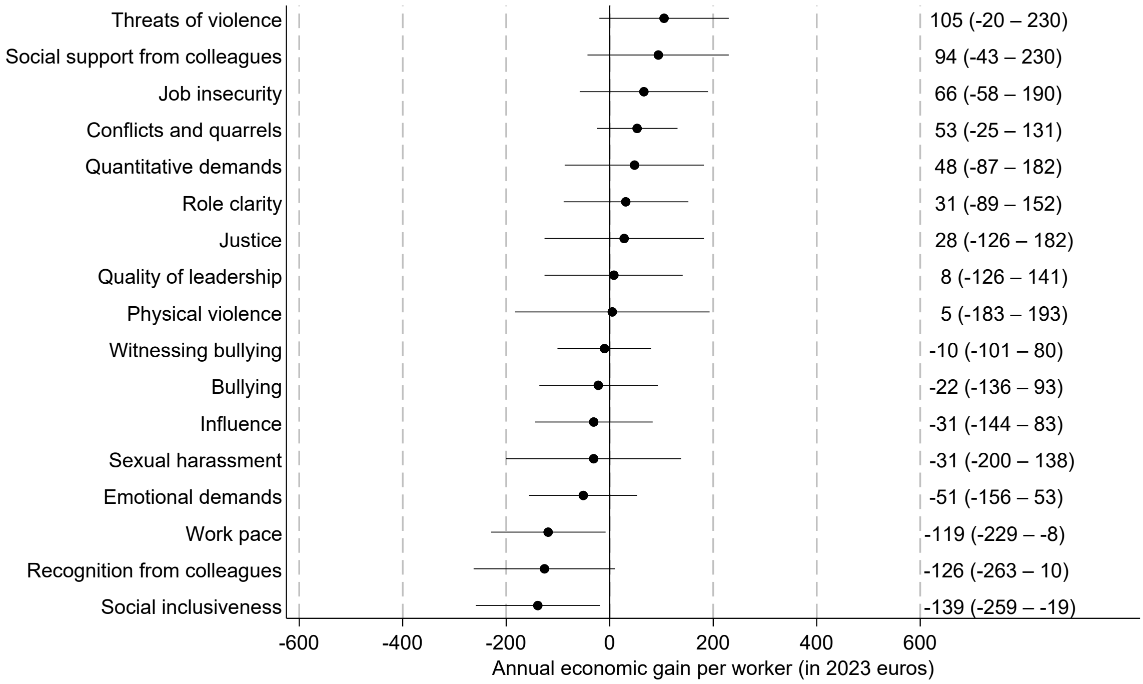

Figure S61. Workers not employed in social institutions. Estimates with 95% confidence intervals for reductions in annual costs of health care use per worker from hypothetical improvements (observed to most desirable) of specific psychosocial work environment factors. Parametric g-formula analyses with adjustment for sociodemographic characteristics, job characteristics, physical work environment characteristics, health status and health behaviors.

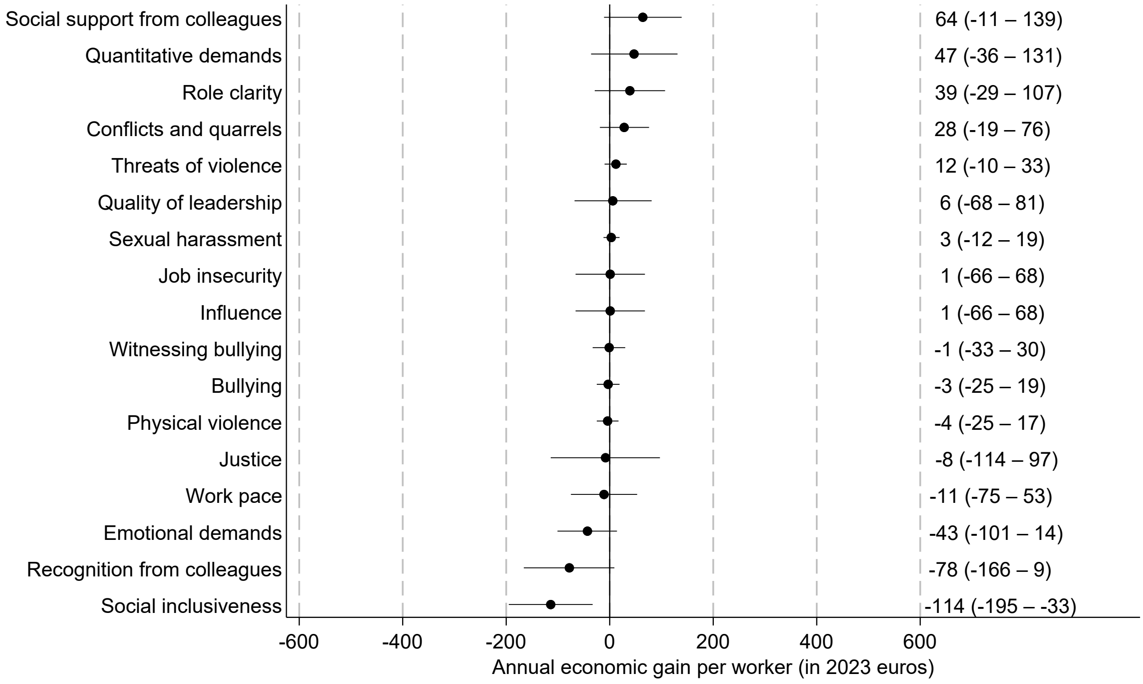

Appendix 6. Sensitivity analyses regarding sickness absence history

All estimates reported in this appendix come from analyses that only included observations from the 2014 to 2018 waves. Figures S62 to S68 report estimates from analyses, which included self-reported days of sickness absence (SA) within the last year in the set of covariates. For comparison, Figures S69 to S75 report estimates from analyses, which only included the covariates used in the main analyses.

More specifically, in the sensitivity analyses, which included self-reported days of SA within the last year in the set of covariates, we did the following: In addition to the covariates listed in Appendix 2, we included (i) an indicator variable indicating whether self-reported information on days of SA within the last year existed and (ii) a fourth order polynomial of the days of SA within the last year. For observations without information on days of SA within the last year, we arbitrarily set the days of SA within the last year to zero.

Figure S62. Estimates with 95% confidence intervals for annual economic gains per worker from two hypothetical improvements of the general psychosocial work environment. Parametric g-formula analyses with adjustment for sociodemographic characteristics, job characteristics, physical work environment characteristics, health status and health behaviors. Observations from the 2014 to 2018 waves only. Includes days of sickness absence within the last year in the set of covariates.

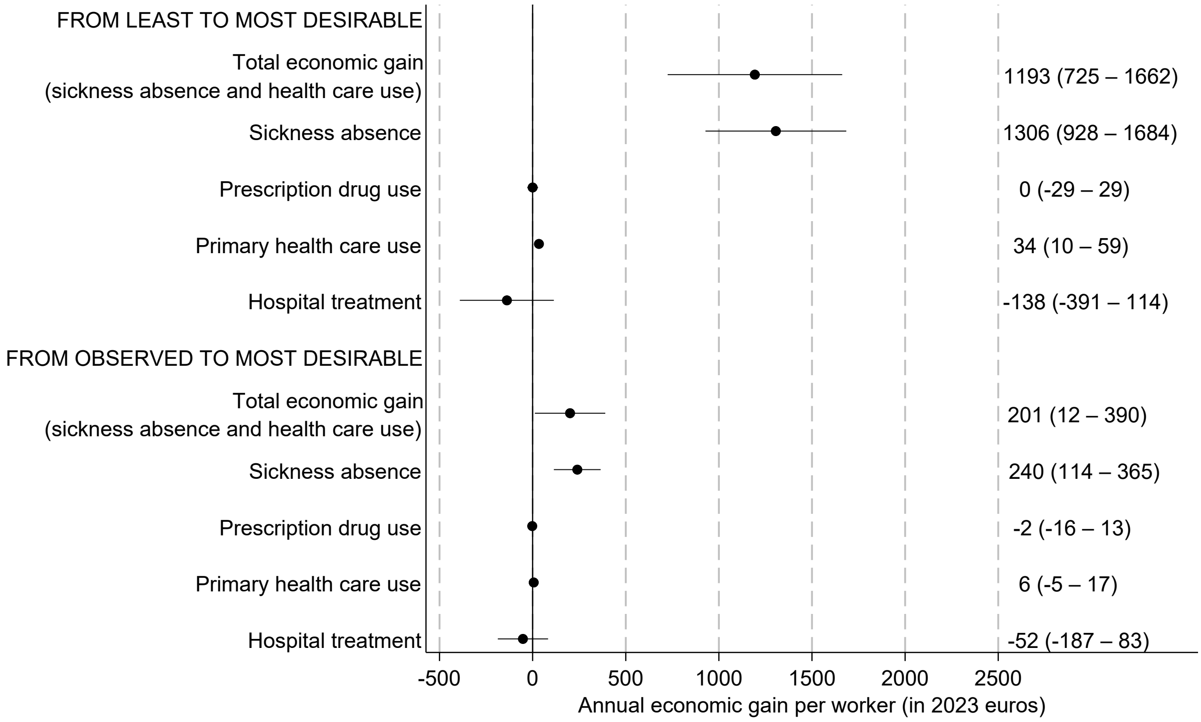

Figure S63. Estimates with 95% confidence intervals for annual economic gains per worker from hypothetical improvements (least to most desirable) of specific psychosocial work environment factors. Parametric g-formula analyses with adjustment for sociodemographic characteristics, job characteristics, physical work environment characteristics, health status and health behaviors. Observations from the 2014 to 2018 waves only. Includes days of sickness absence within the last year in the set of covariates.

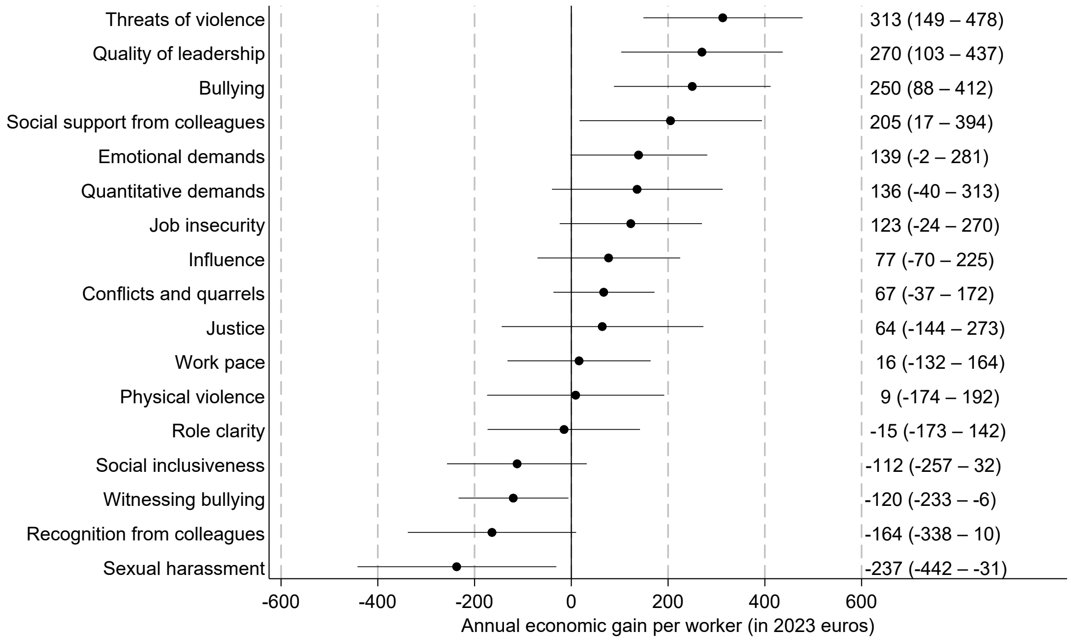

Figure S64. Estimates with 95% confidence intervals for annual economic gains per worker from hypothetical improvements (observed to most desirable) of specific psychosocial work environment factors. Parametric g-formula analyses with adjustment for sociodemographic characteristics, job characteristics, physical work environment characteristics, health status and health behaviors. Observations from the 2014 to 2018 waves only. Includes days of sickness absence within the last year in the set of covariates.

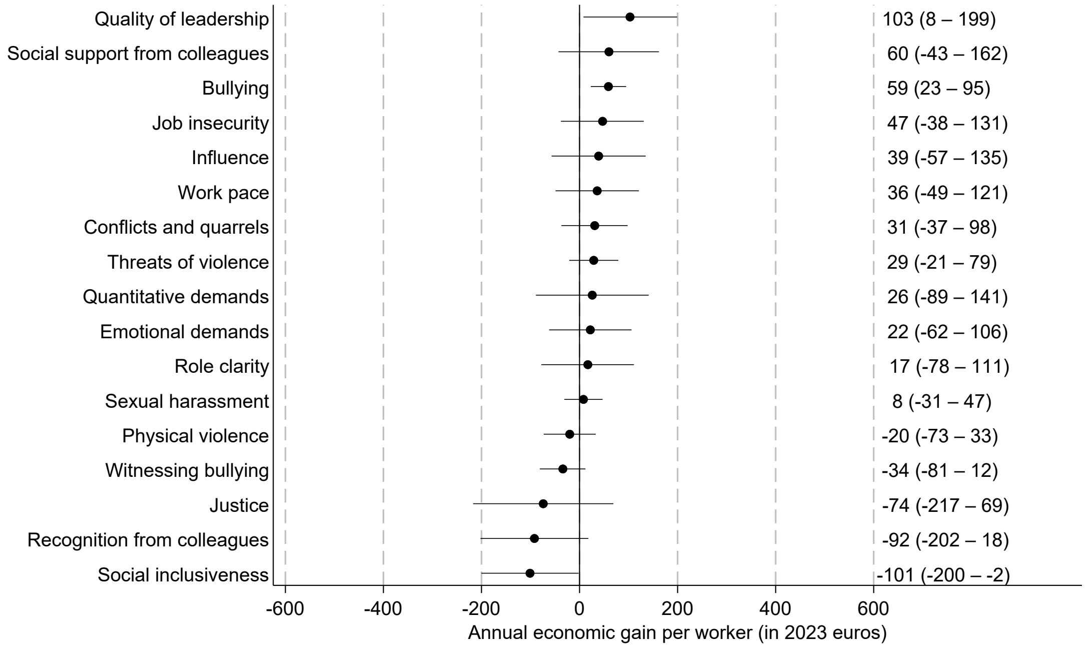

Figure S65. Estimates with 95% confidence intervals for reductions in annual costs of sickness absence per worker from hypothetical improvements (least to most desirable) of specific psychosocial work environment factors. Parametric g-formula analyses with adjustment for sociodemographic characteristics, job characteristics, physical work environment characteristics, health status and health behaviors. Observations from the 2014 to 2018 waves only. Includes days of sickness absence within the last year in the set of covariates.

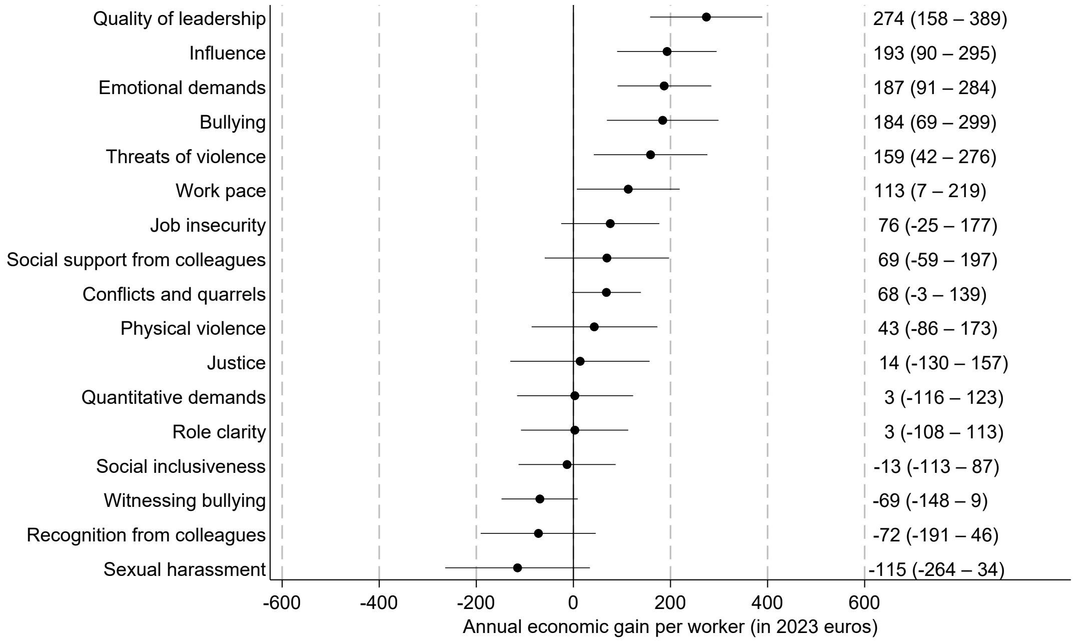

Figure S66. Estimates with 95% confidence intervals for reductions in annual costs of sickness absence per worker from hypothetical improvements (observed to most desirable) of specific psychosocial work environment factors. Parametric g-formula analyses with adjustment for sociodemographic characteristics, job characteristics, physical work environment characteristics, health status and health behaviors. Observations from the 2014 to 2018 waves only. Includes days of sickness absence within the last year in the set of covariates.

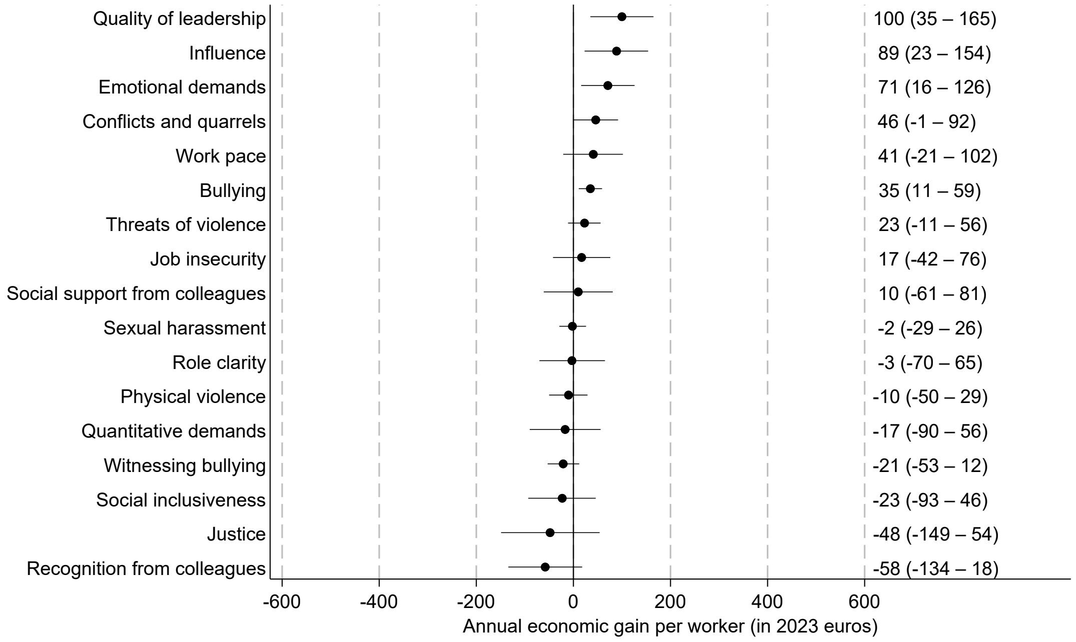

Figure S67. Estimates with 95% confidence intervals for reductions in annual costs of health care use per worker from hypothetical improvements (least to most desirable) of specific psychosocial work environment factors. Parametric g-formula analyses with adjustment for sociodemographic characteristics, job characteristics, physical work environment characteristics, health status and health behaviors. Observations from the 2014 to 2018 waves only. Includes days of sickness absence within the last year in the set of covariates.

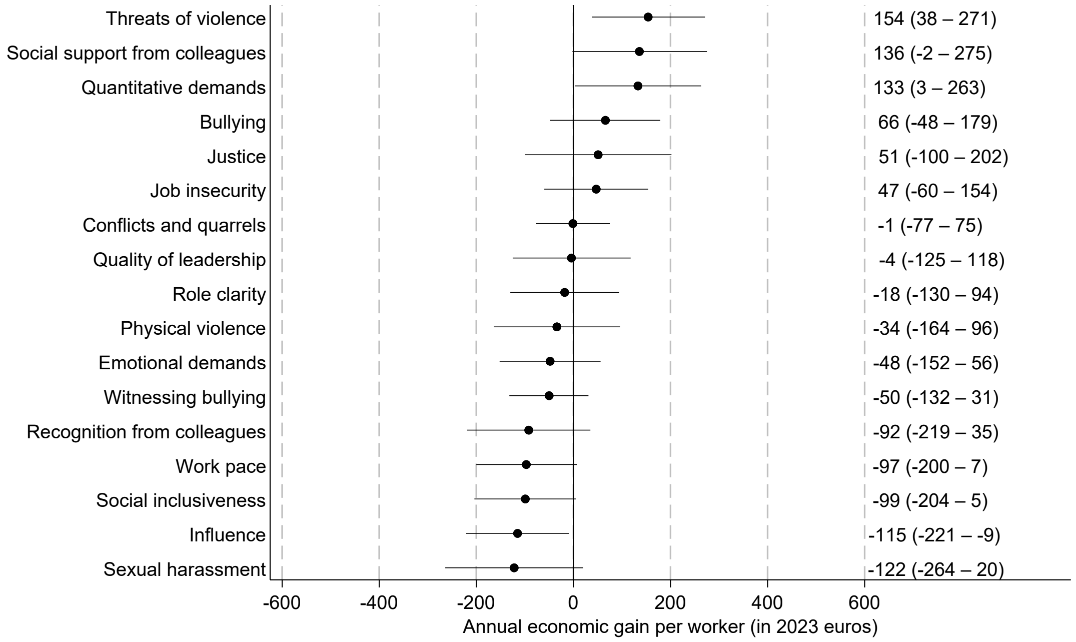

Figure S68. Estimates with 95% confidence intervals for reductions in annual costs of health care use per worker from hypothetical improvements (observed to most desirable) of specific psychosocial work environment factors. Parametric g-formula analyses with adjustment for sociodemographic characteristics, job characteristics, physical work environment characteristics, health status and health behaviors. Observations from the 2014 to 2018 waves only. Includes days of sickness absence within the last year in the set of covariates.

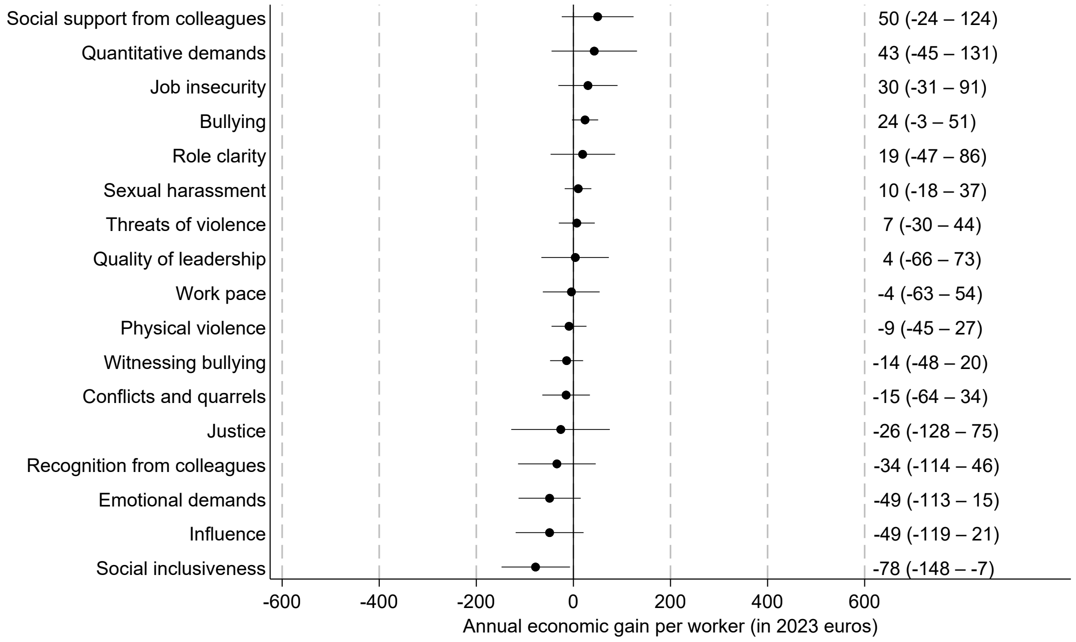

Figure S69. Estimates with 95% confidence intervals for annual economic gains per worker from two hypothetical improvements of the general psychosocial work environment. Parametric g-formula analyses with adjustment for sociodemographic characteristics, job characteristics, physical work environment characteristics, health status and health behaviors. Observations from the 2014 to 2018 waves only.

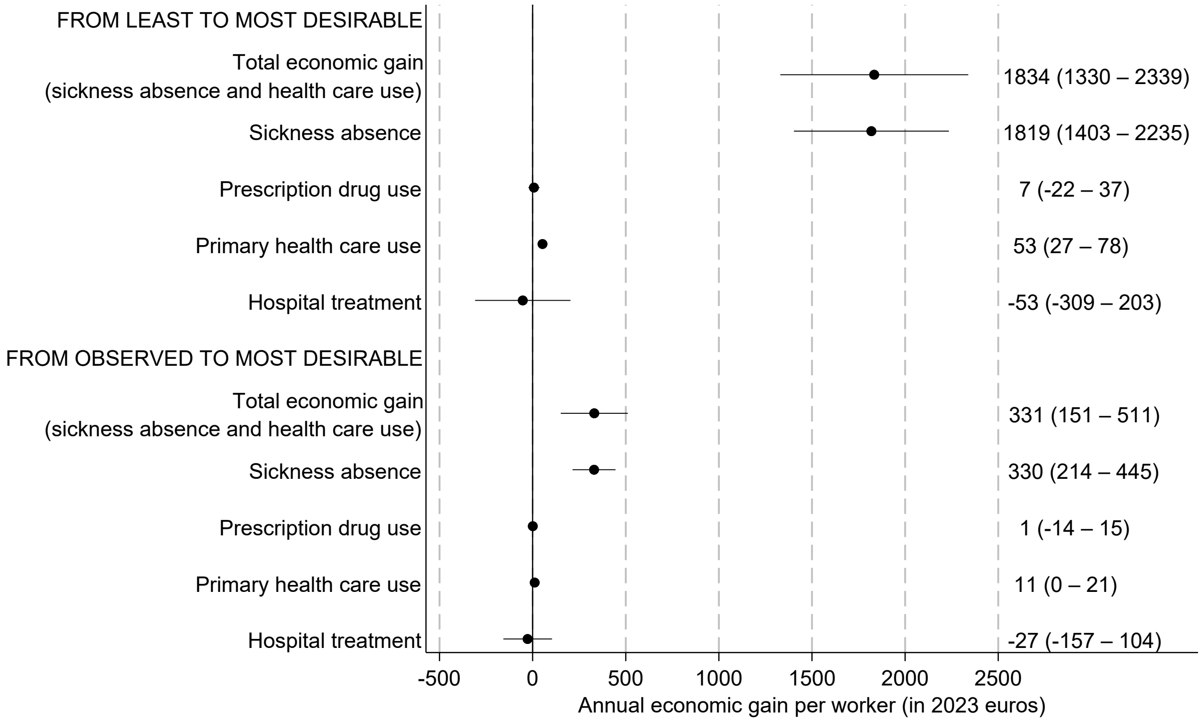

Figure S70. Estimates with 95% confidence intervals for annual economic gains per worker from hypothetical improvements (least to most desirable) of specific psychosocial work environment factors. Parametric g-formula analyses with adjustment for sociodemographic characteristics, job characteristics, physical work environment characteristics, health status and health behaviors. Observations from the 2014 to 2018 waves only.

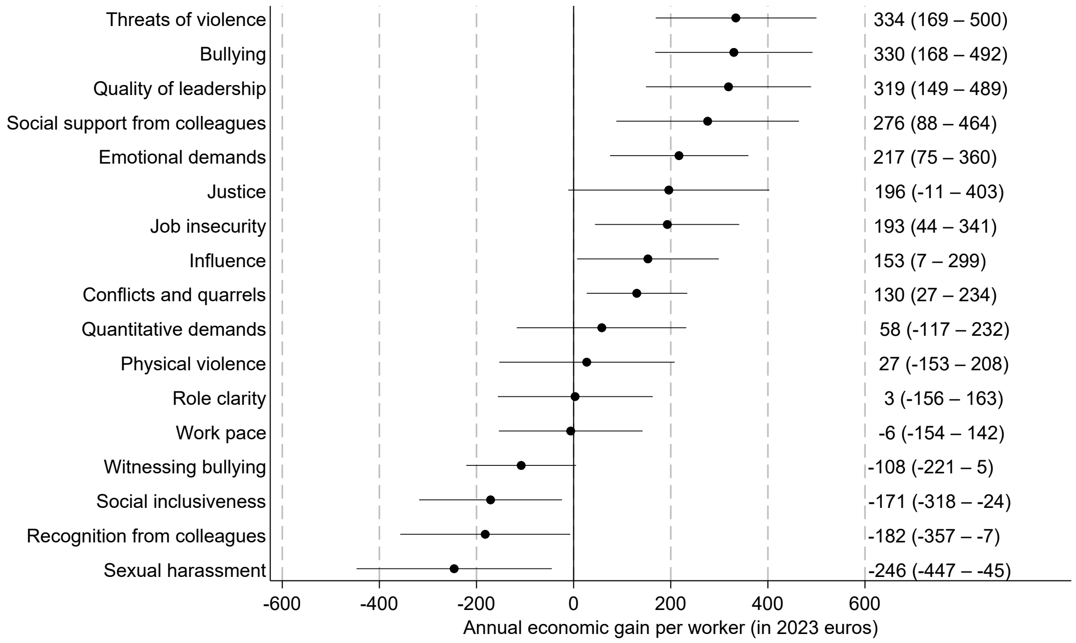

Figure S71. Estimates with 95% confidence intervals for annual economic gains per worker from hypothetical improvements (observed to most desirable) of specific psychosocial work environment factors. Parametric g-formula analyses with adjustment for sociodemographic characteristics, job characteristics, physical work environment characteristics, health status and health behaviors. Observations from the 2014 to 2018 waves only.

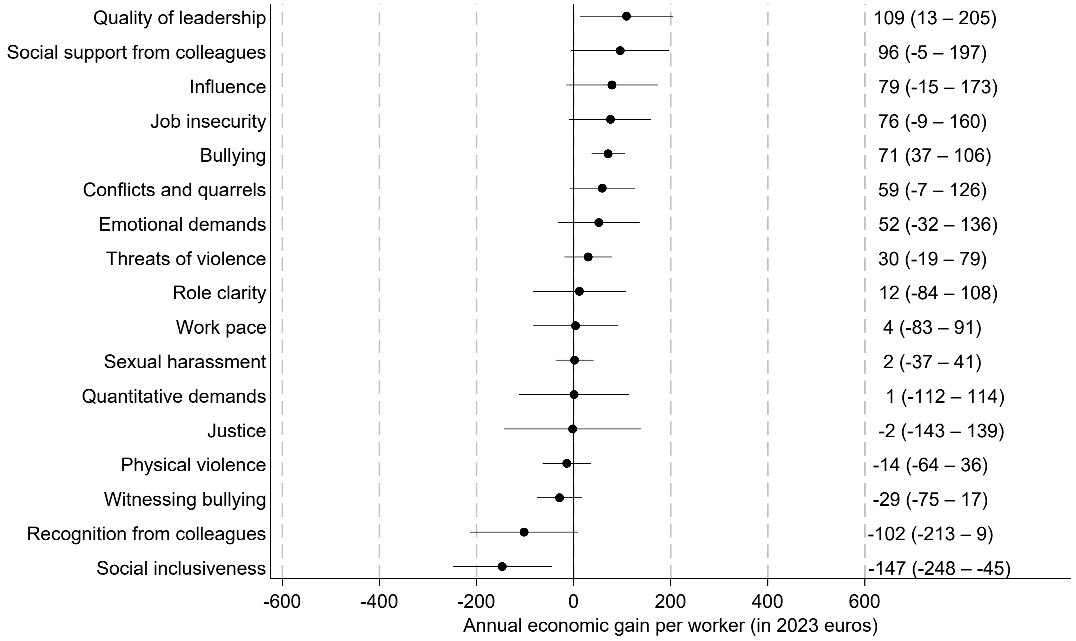

Figure S72. Estimates with 95% confidence intervals for reductions in annual costs of sickness absence per worker from hypothetical improvements (least to most desirable) of specific psychosocial work environment factors. Parametric g-formula analyses with adjustment for sociodemographic characteristics, job characteristics, physical work environment characteristics, health status and health behaviors. Observations from the 2014 to 2018 waves only.

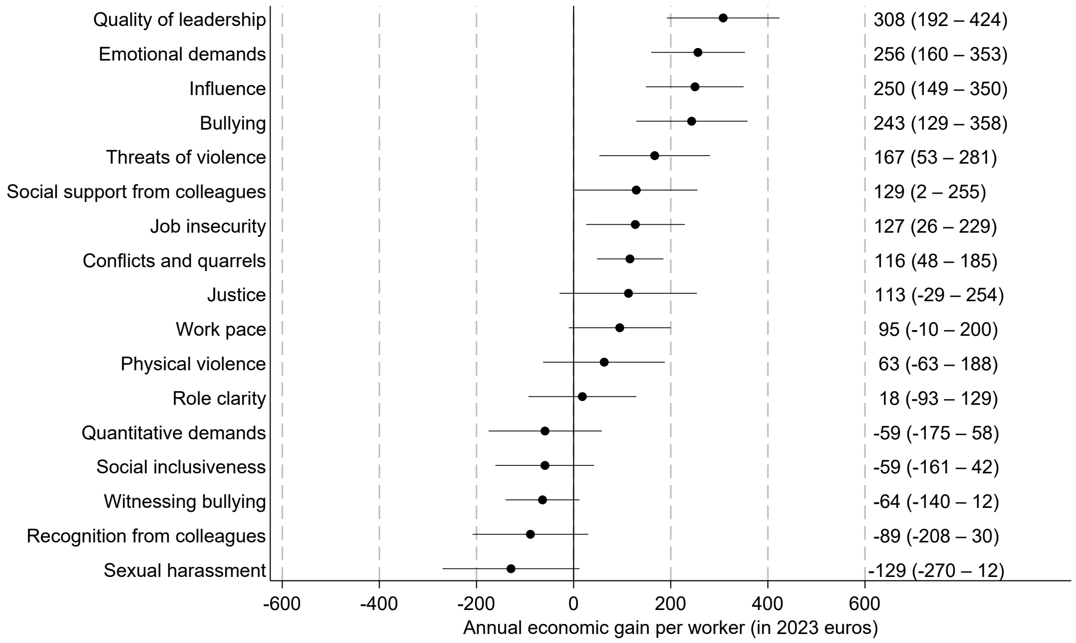

Figure S73. Estimates with 95% confidence intervals for reductions in annual costs of sickness absence per worker from hypothetical improvements (observed to most desirable) of specific psychosocial work environment factors. Parametric g-formula analyses with adjustment for sociodemographic characteristics, job characteristics, physical work environment characteristics, health status and health behaviors. Observations from the 2014 to 2018 waves only.

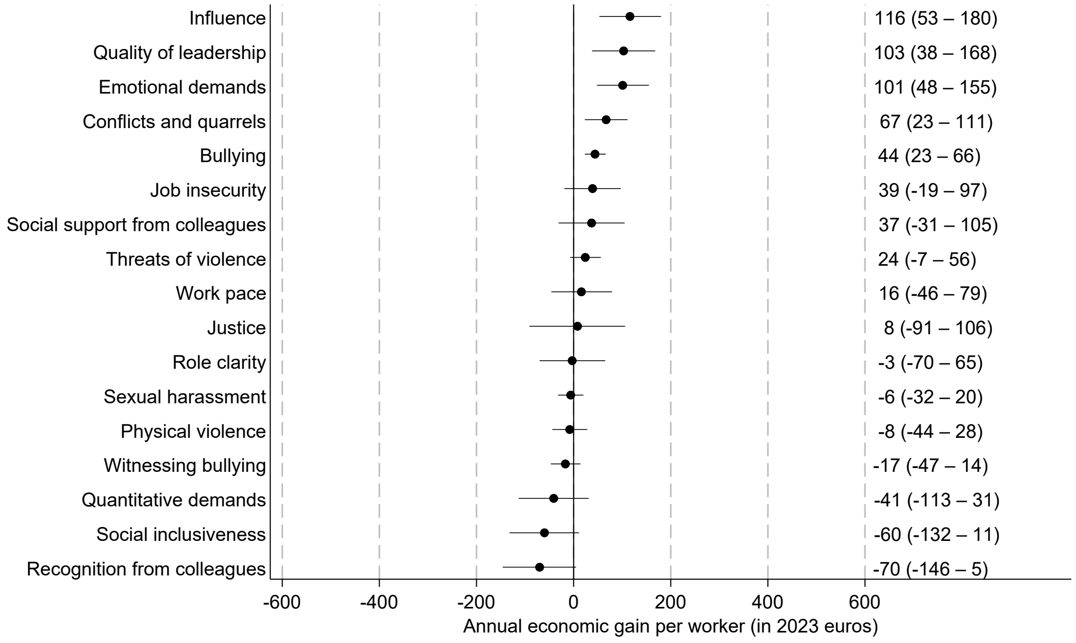

Figure S74. Estimates with 95% confidence intervals for reductions in annual costs of health care use per worker from hypothetical improvements (least to most desirable) of specific psychosocial work environment factors. Parametric g-formula analyses with adjustment for sociodemographic characteristics, job characteristics, physical work environment characteristics, health status and health behaviors. Observations from the 2014 to 2018 waves only.

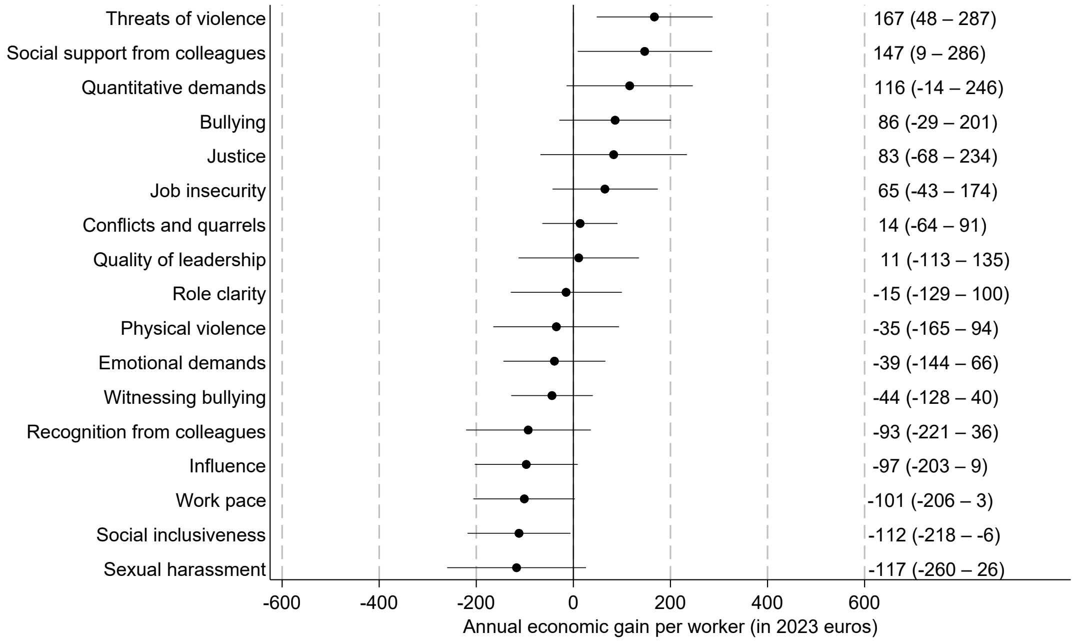

Figure S75. Estimates with 95% confidence intervals for reductions in annual costs of health care use per worker from hypothetical improvements (observed to most desirable) of specific psychosocial work environment factors. Parametric g-formula analyses with adjustment for sociodemographic characteristics, job characteristics, physical work environment characteristics, health status and health behaviors. Observations from the 2014 to 2018 waves only.

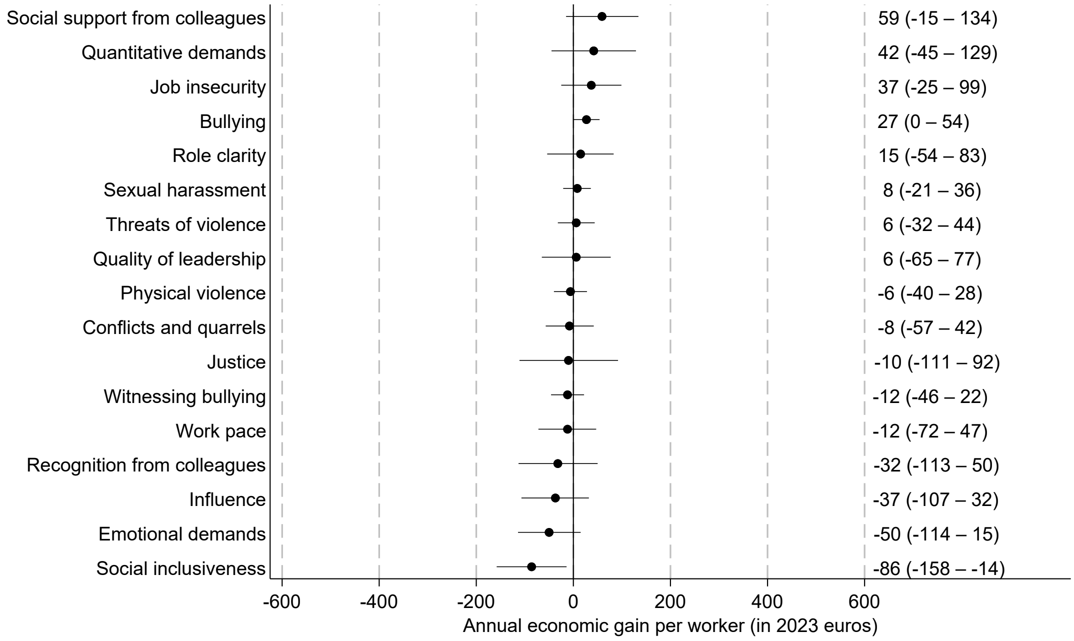

Appendix 7. Sensitivity analyses regarding observations with missing values

Table S9. Estimates with standard errors and 95% confidence intervals for annual economic gains per worker from two hypothetical improvements of the general psychosocial work environment (PSWE). Parametric g-formula analyses with adjustment for sociodemographic characteristics, job characteristics, physical work environment characteristics, health status and health behaviors.

	FROM LEAST TO MOST DESIRABLE				FROM OBSERVED TO MOST DESIRABLE			
	Estimate	Standard error	95% CI, lower bound	95% CI, upper bound	Estimate	Standard error	95% CI, lower bound	95% CI, upper bound
ALL WORKERS (67 780 obs.)								
Total economic gain	1685	230	1234	2135	305	87	134	476
Sickness absence	1705	185	1343	2067	301	53	196	406
Health care use	-21	137	-289	247	4	69	-131	139
WORKERS WITH NON-MISSING VALUES FOR ALL PSWE FACTORS (65 040 obs.)								
Total economic gain	1612	232	1157	2067	261	90	85	436
Sickness absence	1672	187	1305	2038	281	55	173	389
Health care use	-60	138	-329	210	-20	71	-159	118
WORKERS WITH NON-MISSING VALUES FOR ALL EXPLANATORY VARIABLES (59 445 obs)[a]								
Total economic gain	1673	246	1191	2156	258	94	74	442
Sickness absence	1694	198	1306	2081	275	58	161	388
Health care use	-20	147	-308	268	-17	74	-161	128

Notes: Numbers of observations in the table are numbers of observations used to estimate the regression models.

[a] Explanatory variables include all PSWE factors and covariates.

Figure S76. Workers with non-missing values for all psychosocial work environment (PSWE) factors. Estimates with 95% confidence intervals for annual economic gains per worker from hypothetical improvements (least to most desirable) of specific PSWE factors. Parametric g-formula analyses with adjustment for sociodemographic characteristics, job characteristics, physical work environment characteristics, health status and health behaviors.

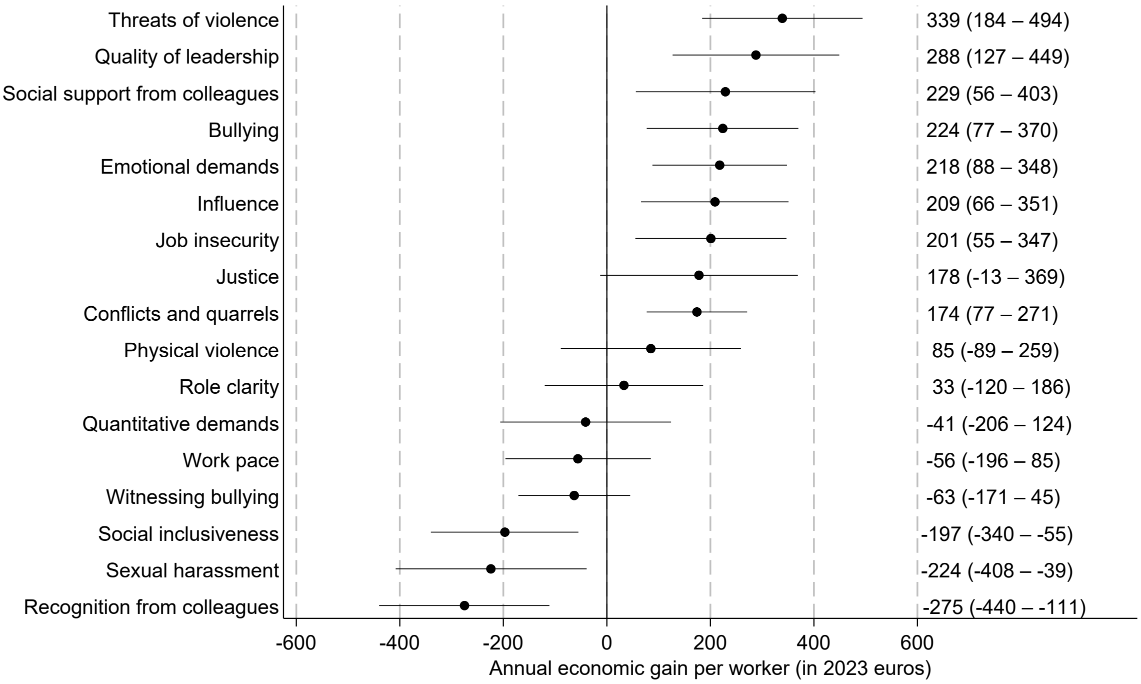

Figure S77. Workers with non-missing values for all psychosocial work environment (PSWE) factors. Estimates with 95% confidence intervals for annual economic gains per worker from hypothetical improvements (observed to most desirable) of specific PSWE factors. Parametric g-formula analyses with adjustment for sociodemographic characteristics, job characteristics, physical work environment characteristics, health status and health behaviors.

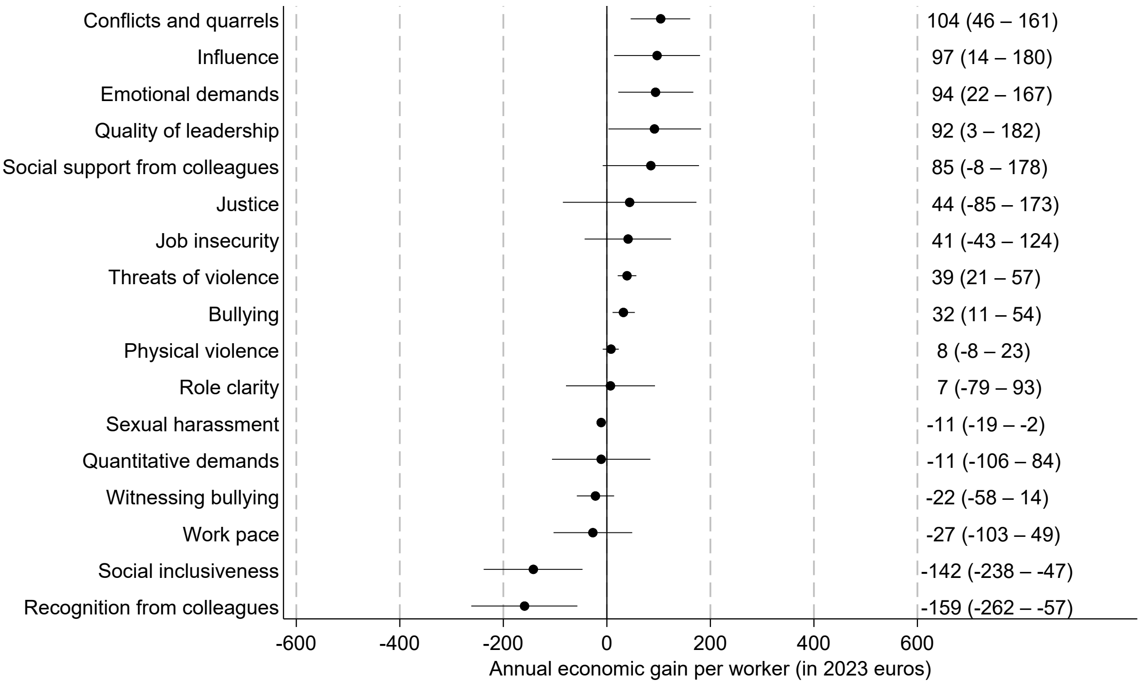

Figure S78. Workers with non-missing values for all psychosocial work environment (PSWE) factors. Estimates with 95% confidence intervals for reductions in annual costs of sickness absence per worker from hypothetical improvements (least to most desirable) of specific PSWE factors. Parametric g-formula analyses with adjustment for sociodemographic characteristics, job characteristics, physical work environment characteristics, health status and health behaviors.

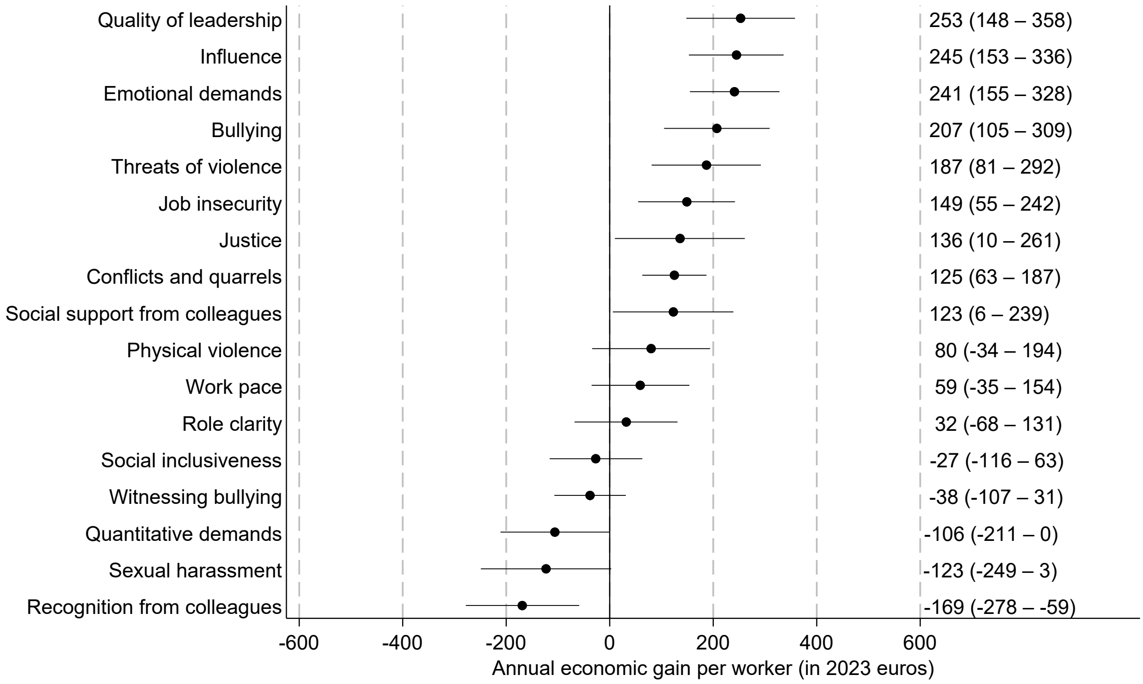

Figure S79. Workers with non-missing values for all psychosocial work environment (PSWE) factors. Estimates with 95% confidence intervals for reductions in annual costs of sickness absence per worker from hypothetical improvements (observed to most desirable) of specific PSWE factors. Parametric g-formula analyses with adjustment for sociodemographic characteristics, job characteristics, physical work environment characteristics, health status and health behaviors.

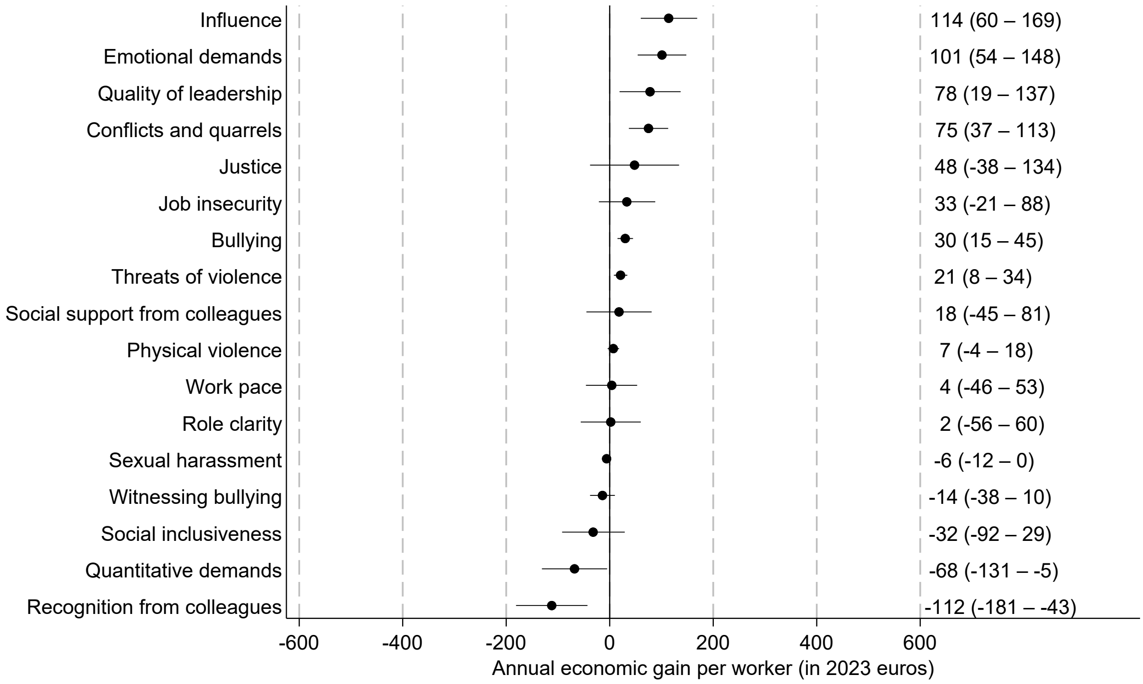

Figure S80. Workers with non-missing values for all psychosocial work environment (PSWE) factors. Estimates with 95% confidence intervals for reductions in annual costs of health care use per worker from hypothetical improvements (least to most desirable) of specific PSWE factors. Parametric g-formula analyses with adjustment for sociodemographic characteristics, job characteristics, physical work environment characteristics, health status and health behaviors.

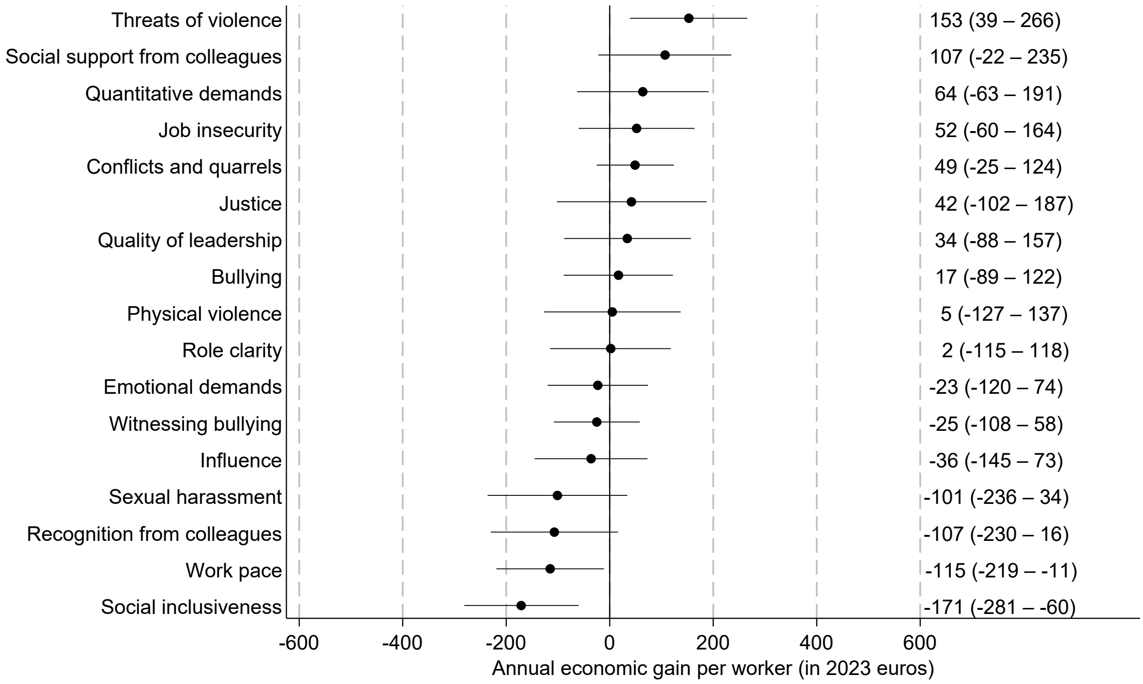

Figure S81. Workers with non-missing values for all psychosocial work environment (PSWE) factors. Estimates with 95% confidence intervals for reductions in annual costs of health care use per worker from hypothetical improvements (observed to most desirable) of specific PSWE factors. Parametric g-formula analyses with adjustment for sociodemographic characteristics, job characteristics, physical work environment characteristics, health status and health behaviors.

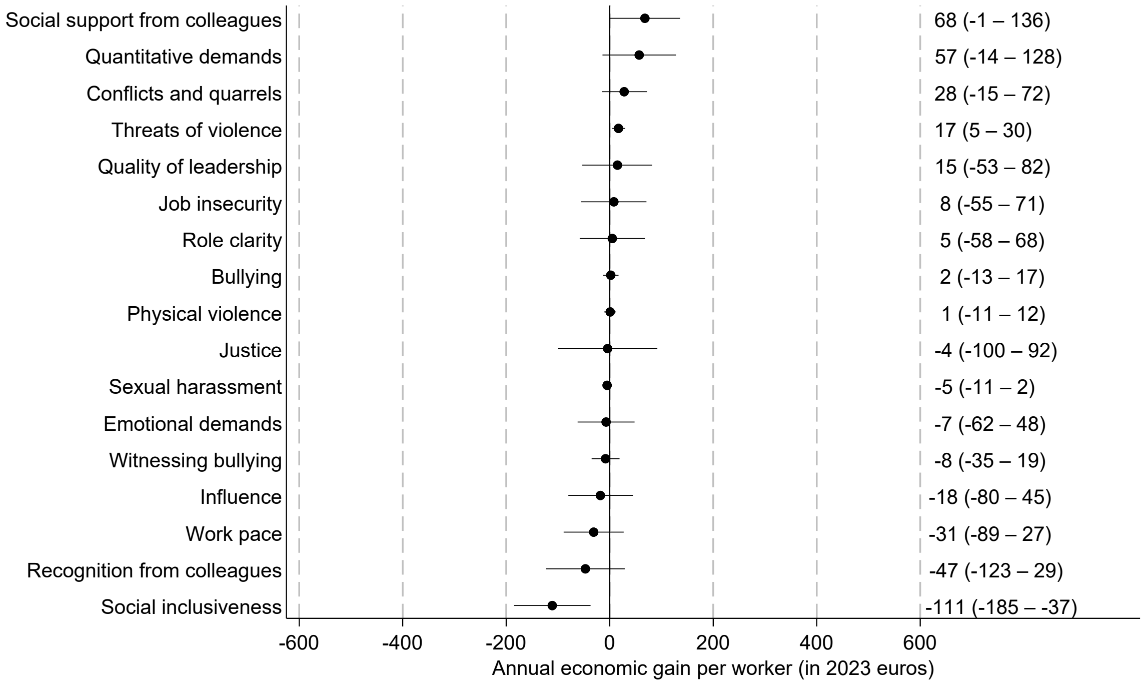

Figure S82. Workers with non-missing values for all explanatory variables. Estimates with 95% confidence intervals for annual economic gains per worker from hypothetical improvements (least to most desirable) of specific psychosocial work environment factors. Parametric g-formula analyses with adjustment for sociodemographic characteristics, job characteristics, physical work environment characteristics, health status and health behaviors.

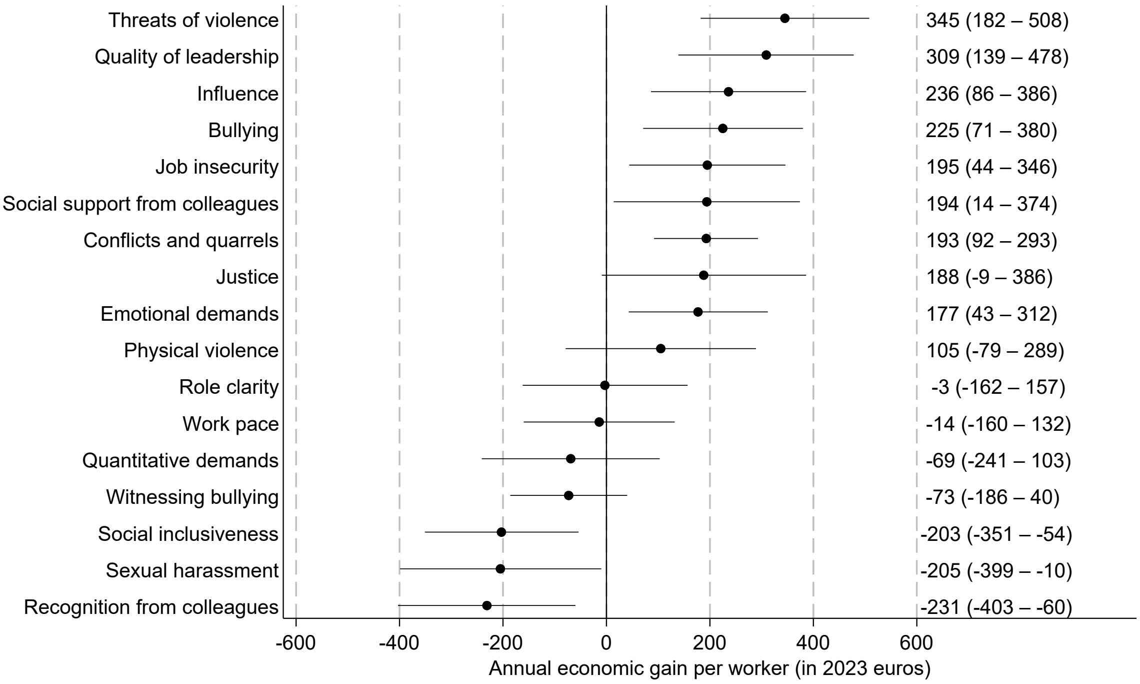

Figure S83. Workers with non-missing values for all explanatory variables. Estimates with 95% confidence intervals for annual economic gains per worker from hypothetical improvements (observed to most desirable) of specific psychosocial work environment factors. Parametric g-formula analyses with adjustment for sociodemographic characteristics, job characteristics, physical work environment characteristics, health status and health behaviors.

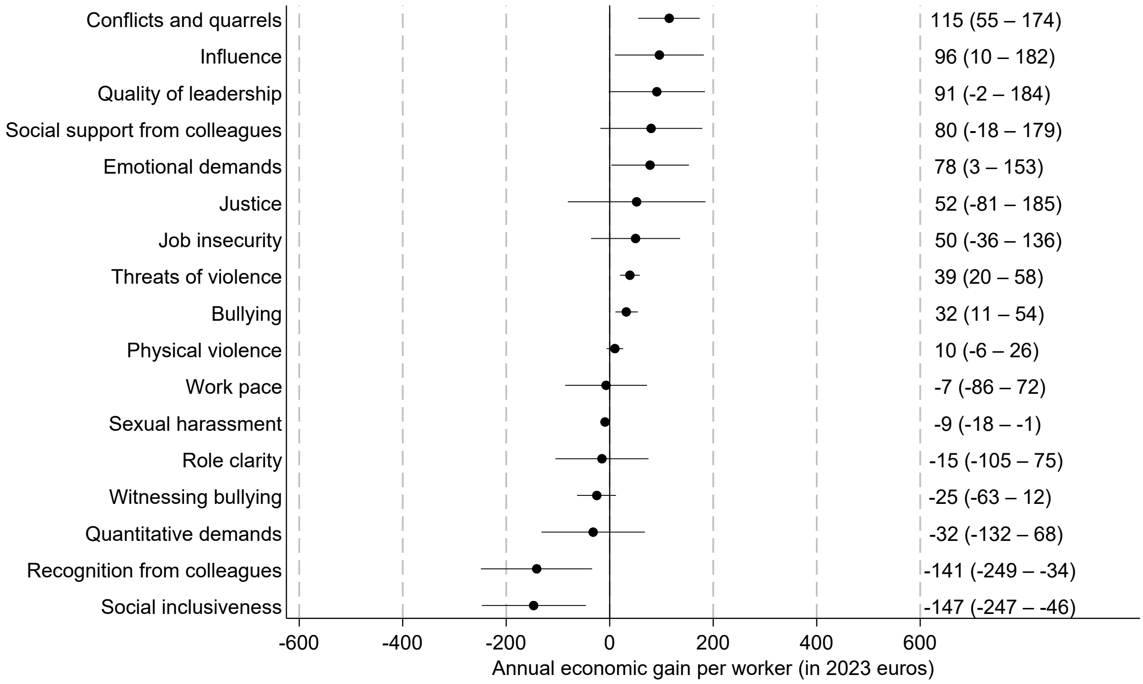

Figure S84. Workers with non-missing values for all explanatory variables. Estimates with 95% confidence intervals for reductions in annual costs of sickness absence per worker from hypothetical improvements (least to most desirable) of specific psychosocial work environment factors. Parametric g-formula analyses with adjustment for sociodemographic characteristics, job characteristics, physical work environment characteristics, health status and health behaviors.

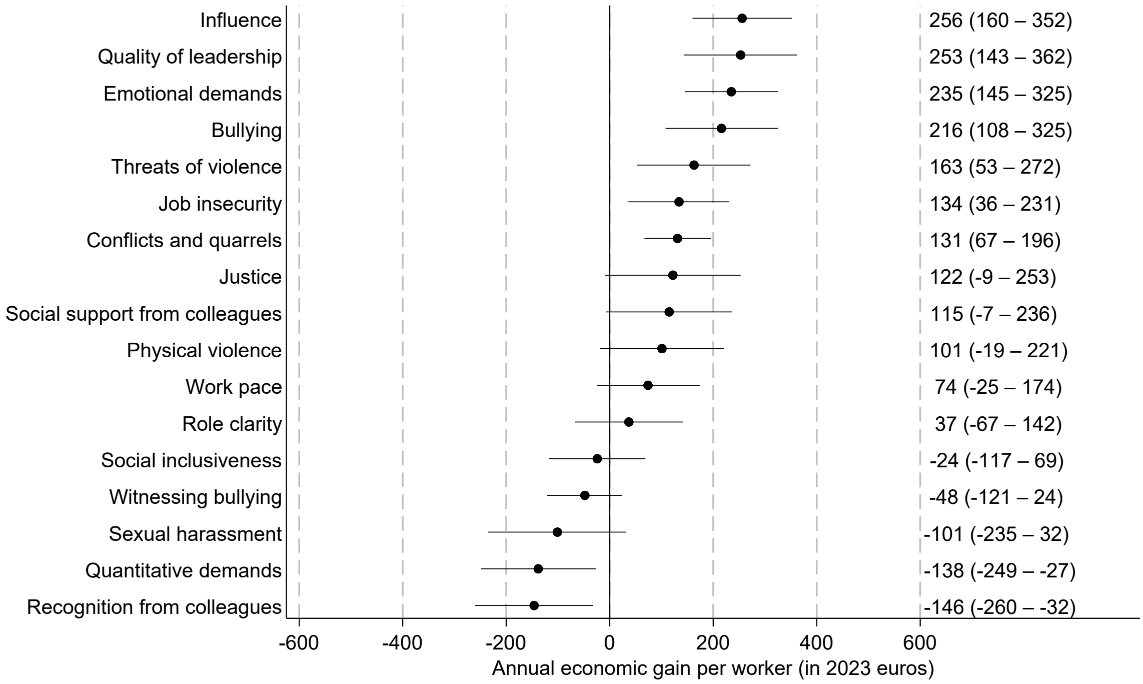

Figure S85. Workers with non-missing values for all explanatory variables. Estimates with 95% confidence intervals for reductions in annual costs of sickness absence per worker from hypothetical improvements (observed to most desirable) of specific psychosocial work environment factors. Parametric g-formula analyses with adjustment for sociodemographic characteristics, job characteristics, physical work environment characteristics, health status and health behaviors.

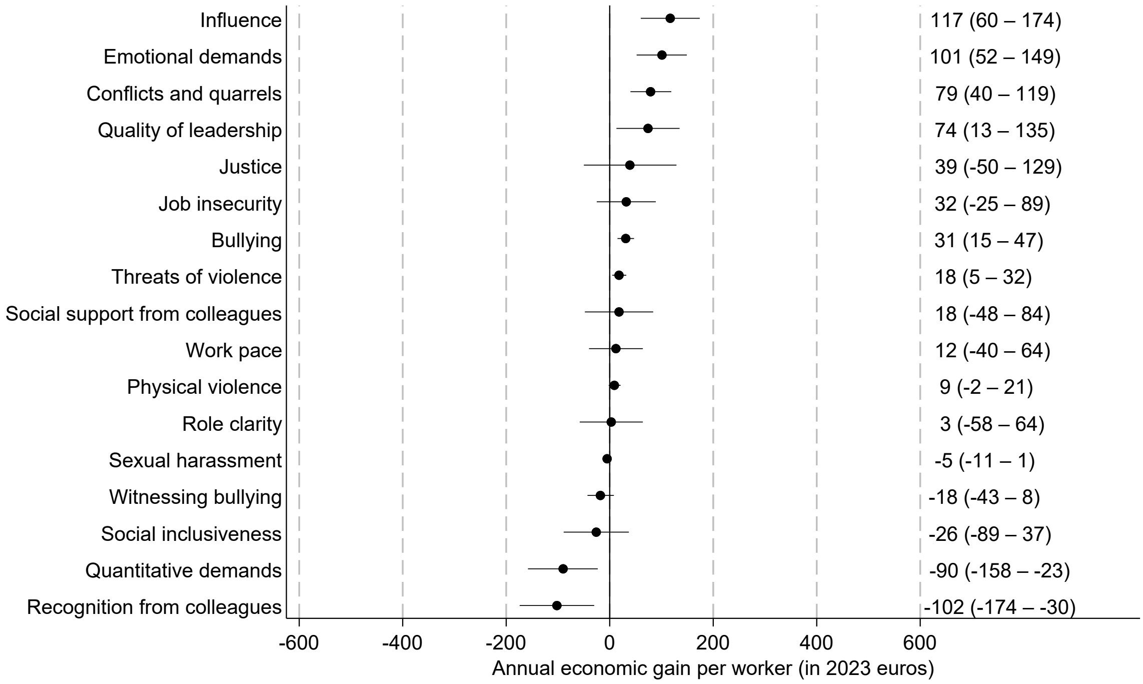

Figure S86. Workers with non-missing values for all explanatory variables. Estimates with 95% confidence intervals for reductions in annual costs of health care use per worker from hypothetical improvements (least to most desirable) of specific psychosocial work environment factors. Parametric g-formula analyses with adjustment for sociodemographic characteristics, job characteristics, physical work environment characteristics, health status and health behaviors.

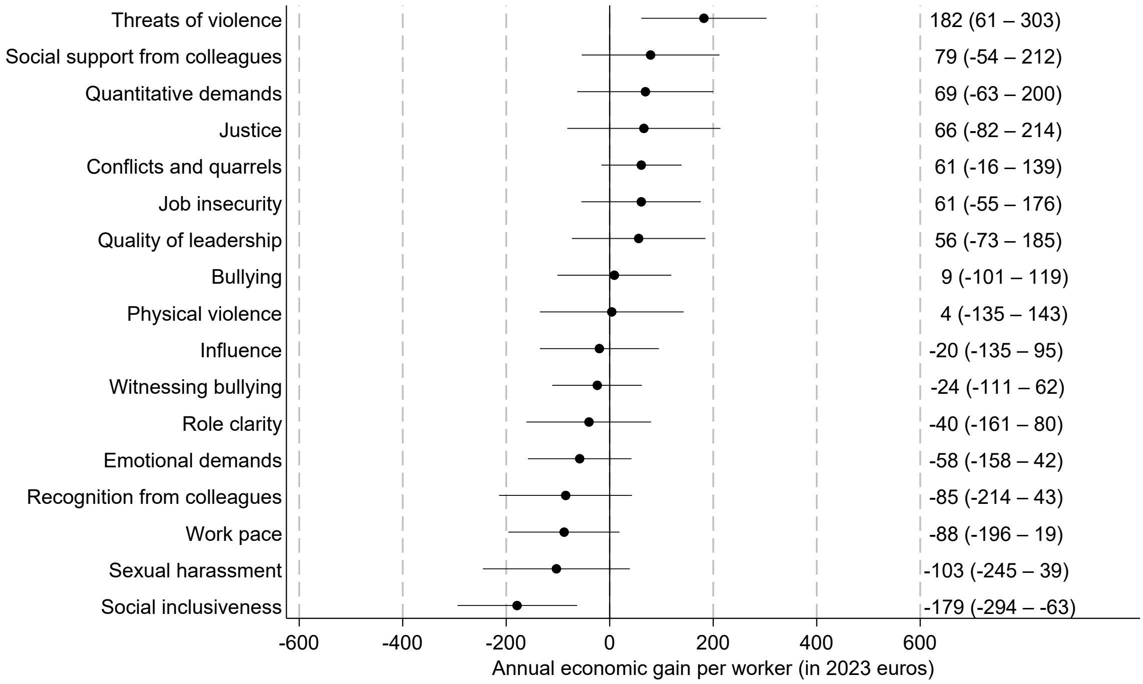

Figure S87. Workers with non-missing values for all explanatory variables. Estimates with 95% confidence intervals for reductions in annual costs of health care use per worker from hypothetical improvements (observed to most desirable) of specific psychosocial work environment factors. Parametric g-formula analyses with adjustment for sociodemographic characteristics, job characteristics, physical work environment characteristics, health status and health behaviors.

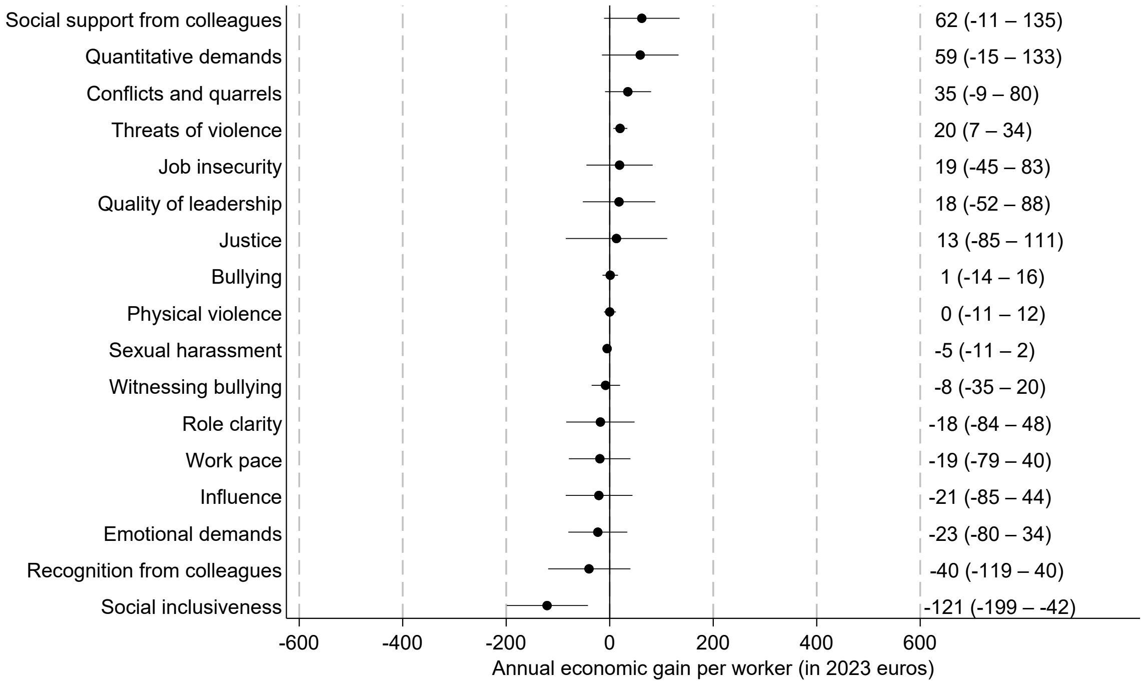

References

1. International Labour Office. International standard classification of occupations (ISCO-08), Volume 1: Structure, group definitions and correspondence tables. Geneve: International Labour Office; 2012.
2. Mullahy J. Specification and testing of some modified count data models. J Econom. 1986;33(3):341-65.
3. Greene WH. Econometric analysis, 8th edition. New York: Pearson; 2017.
4. Wooldridge JM. Econometric Analysis of Cross Section and Panel Data. Cambridge, Massachusetts: MIT Press; 2002.
